# From Phenotype to Genome: A Critical Evidence-Based Synthesis of Frosty Pod Rot (*Moniliophthora roreri*) Resistance in Cacao and a Proposed Evidence-Gated Framework to Support Genetic Gain

**DOI:** 10.3390/ijms27209294

**Published:** 2026-10-19

**Authors:** Yirlis Yadeth Pineda-Rodriguez, Juan Jaraba Navas, Alfredo Jarma-Orozco, Luis Eliecer Oviedo-Zumaque, Luis Alfonso Rodríguez-Páez

**Affiliations:** 1 Laboratorio de Biología Molecular Aplicada, Facultad de Ciencias Agrícolas, Universidad de Córdoba, Montería 230002, Colombia; yirlispinedar@correo.unicordoba.edu.co (Y.Y.P.-R.); jjaraba@correo.unicordoba.edu.co (J.J.N.); ajarma@correo.unicordoba.edu.co (A.J.-O.); 2 Facultad de Ciencias Básicas, Universidad de Córdoba, Montería 230002, Colombia; leoviedo@correo.unicordoba.edu.co

**Keywords:** *Moniliophthora roreri*, frosty pod rot, *Theobroma cacao*, cacao breeding, genomic selection, genome-wide association study (GWAS), quantitative trait loci (QTL), molecular breeding, functional validation, precision breeding

## Abstract

Frosty pod rot (FPR), caused by *Moniliophthora roreri*, severely constrains cacao production in the Neotropics, yet evidence supporting molecular breeding for resistance remains fragmented and is often conflated with findings from witches’ broom disease. We conducted a structured critical review, not a systematic review, and classified each FPR-related claim with an author-developed, multidimensional evidence classification system (E0–E5) separating directness, genetic replication, mechanistic inference and breeding utility. The evidence supports a quantitative, partial and context-dependent architecture of resistance. The strongest QTL have been re-detected only within the Pound 7 × UF273 pedigree (E2a), and no FPR locus replicated in genetically independent material (E2b) was identified in the literature retrieved and verified. Genomic predictive ability reached 0.60–0.67 under random cross-validation within one clonal panel but averaged approximately 0.16 when models were transferred among related populations; these values answer different questions and are not directly comparable. Host and pathogen transcriptomics provide correlative evidence (E3a), and recombinant *M. roreri* effectors show biological activity in heterologous or cacao tissues (E3b-i), but no stable genetic manipulation altering FPR in a pod-challenge assay (E4) and no documented breeding utility (E5) were identified. We propose a Phenotyping and Reporting Guideline for FPR (PRG-FPR) and MIGGA, a modular, evidence-gated decision framework illustrated with a retrospective worked example; its effect on realised genetic gain remains to be tested.

## 1. Problem and Scope: Why Frosty Pod Rot Needs Its Own Synthesis

Cacao (*Theobroma cacao* L.) sustains the livelihoods of millions of smallholder households across the tropical belt and underpins rural economies in West Africa, Southeast Asia and the Neotropics, the species’ centre of origin and genetic diversity [1,2]. The major phytosanitary constraints affecting the crop differ geographically. Black pod disease (*Phytophthora* spp.) and cacao swollen shoot virus disease are particularly consequential in West Africa, whereas frosty pod rot (FPR), witches’ broom disease (WBD) and *Phytophthora* pod rots collectively impose major constraints across the Americas. Among them, FPR, caused by the hemibiotrophic basidiomycete *Moniliophthora roreri* (Cif.) H.C. Evans, Stalpers, Samson & Benny is one of the most damaging diseases of cacao in the Neotropics [3,4]. Once restricted largely to northwestern South America, *M. roreri* has expanded progressively through Central America and parts of the Caribbean, and its continued invasive potential represents a persistent phytosanitary threat to currently unaffected cacao-growing regions [3,4].

Cultural and chemical interventions can substantially reduce FPR incidence when applied consistently, particularly through frequent removal of infected pods and integrated canopy and crop management. Their effectiveness, however, depends on recurrent labour and timely implementation and may be difficult to sustain economically in smallholder production systems [3,5]. Genetic improvement for reduced disease severity and stable productivity therefore remains a central component of long-term FPR management. Progress has nevertheless been slow. Cacao combines a multi-year juvenile phase with a complex late-acting self-incompatibility system [6], while FPR resistance itself is quantitative, environmentally sensitive and incompletely resolved genetically. Published heritability estimates vary substantially among populations and experimental designs, and the heterogeneous phenotyping procedures used across mapping and breeding studies further complicate comparison and replication of resistance signals.

Genomic resources have expanded rapidly. QTL mapping, genome-wide association analyses, genomic prediction, pangenomic resources, machine learning approaches, transcriptomics and gene-editing technologies now provide increasingly sophisticated routes for dissecting and potentially exploiting disease resistance. Yet technological availability should not be confused with evidentiary maturity. For FPR specifically, statistical associations substantially outnumber independently replicated loci; direct molecular evidence exists for both the cacao host and *M. roreri*, but stable causal validation of FPR resistance candidates remains absent, and no marker, candidate gene or genomic-prediction model has yet demonstrated documented breeding utility at the highest evidence level considered in this review. Evidence generated for WBD or other cacao pathosystems is also frequently invoked when discussing FPR, even when the underlying pathogen, phenotype or causal mechanism has not been tested directly.

This review therefore asks a deliberately narrower and more consequential question than whether modern molecular tools are available for cacao improvement: what genetic, genomic and functional evidence actually supports genetic gain for FPR resistance, and what evidence is still required before those findings can justify breeding decisions? We address this question by separating FPR-specific evidence from WBD-derived evidence and cross-pathosystem extrapolation; classifying genetic and mechanistic claims with an explicit, multidimensional evidence classification system; identifying phenotyping and validation bottlenecks; and translating the resulting synthesis into MIGGA, a proposed modular, decision-gated framework in which progression from established breeding tools to more speculative molecular interventions depends on prospectively specified evidentiary criteria rather than technological novelty.

Two types of content are distinguished throughout, and the distinction is signalled in the text. Section 2, Section 3, Section 4, Section 5, Section 6 and Section 7 synthesise and appraise published evidence. The evidence classification system (Table 1), the Phenotyping and Reporting Guideline for FPR (PRG-FPR; Table 2), the MIGGA tiers and decision gates (Section 9), the illustrative experimental designs (Section 6.3 and Section 8.3) and the research priorities (Section 11) are author-derived proposals. They are grounded in the reviewed evidence and in established quantitative-genetic and plant-pathological methodology, but none has been validated empirically, and terms such as “gate”, “GO/NO-GO”, “criterion” or “priority” denote proposed decision rules rather than established standards. Section 10 is contextual: it summarises deployment, regulatory, diversity and access-and-benefit-sharing considerations that condition the use of genetic gain, without forming part of the evidence synthesis.

## 2. Review Methodology and Evidence-Classification Framework

### 2.1. Review Type, Scope and What This Design Can and Cannot Support

This work was designed as a structured critical review and evidence synthesis [13] rather than as a formal systematic review. No protocol was preregistered, no PRISMA-based screening workflow was implemented, and formal independent duplicate screening was not conducted prospectively. Exact database-specific retrieval counts, duplicate-removal counts and the complete screening history were not retained in a form that would permit faithful retrospective reconstruction. We therefore do not report a PRISMA flow diagram, retrospectively generated screening counts or inter-reviewer agreement statistics. We did not retrospectively reconstruct exact record counts, because doing so after completion of the original search would risk introducing inaccuracies and would give the false impression of a prospectively documented systematic-review workflow. PRISMA remains a valuable transparency instrument, but it describes a study-selection workflow that this review did not implement, and invoking it retrospectively would misrepresent the design rather than strengthen it. Similarly, Cohen’s κ was not calculated, because the review did not employ prospectively planned independent duplicate screening or duplicate evidence grading; a retrospectively generated κ would not represent the review process actually conducted and could therefore be misleading.

Instead, we report the databases searched, the search concepts and Boolean framework, the eligibility logic, the citation-chaining strategy, the targeted primary-source verification procedure, the evidence-classification rules and the search cut-off dates. A complementary update search conducted during peer review was documented prospectively and is reported separately in Supplementary Table S2 (Section 2.2). The original literature search and evidence-verification process was completed on 30 August 2026. The manuscript was consolidated and formatted on 31 August 2026. Following peer review, a complementary update search was subsequently conducted using an expanded database and search-term framework; this update was completed on 1 October 2026 and is documented separately in Supplementary Table S2. Accordingly, statements of apparent evidence absence refer specifically to the literature retrieved and verified through the stated strategy and cut-off date and should not be interpreted as formal demonstrations that such evidence does not exist (Section 2.5).

### 2.2. Information Sources, Search Framework and Complementary Update Search

The initial search covered PubMed/MEDLINE, Web of Science, Scopus and Google Scholar, and the original search and verification process was completed on 30 August 2026. Google Scholar was used for supplementary discovery, citation chaining and identification of regional or otherwise difficult-to-retrieve literature, and not as a primary reproducible source, because its retrieval is neither fully transparent nor stable [14]. No lower publication-year limit and no language restriction were applied; Spanish- and Portuguese-language records were screened.

In response to the reviewers, a complementary update search was conducted during revision and was documented prospectively. It extended coverage to CAB Abstracts, AGRIS, SciELO and Redalyc, which index much of the Latin American agronomic and breeding literature, and it broadened the resistance concept block to capture breeding and clonal-selection studies that do not use molecular vocabulary. The added terms were breeding, clone selection, clonal evaluation, combining ability, diallel, progeny trial, heritability, genetic parameters, disease incidence, disease severity, tolerance, field resistance, artificial inoculation, productivity, yield, genotype × environment interaction, stability and disease–production indices. The generic form of the string was (“Moniliophthora roreri” OR “Monilia roreri” OR “frosty pod rot” OR moniliasis OR “moniliophthora pod rot”) AND (cacao OR cocoa OR “Theobroma cacao”) AND (resist* OR toleran* OR suscept* OR breed* OR clon* OR “combining ability” OR diallel OR progen* OR heritab* OR “genetic parameter*” OR incidence OR severity OR inoculat* OR yield OR productiv* OR “genotype by environment” OR stability OR “disease and production index” OR QTL OR GWAS OR “association mapping” OR “genomic selection” OR “genomic prediction” OR marker* OR transcriptom* OR proteom* OR metabolom* OR effector* OR “candidate gene*” OR CRISPR OR “gene editing”).

The complementary update search conducted during revision was prospectively documented in full, including the search date, the database-specific query, the retrieved record count, the number of records assessed as potentially relevant, the duplicates detected, the disposition of each newly identified record with its reason for exclusion, and the person who performed the verification. The complementary update search was completed on 1 October 2026, and its database-specific queries, retrieved-record counts, screening disposition and verification details are reported in Supplementary Table S2. Newly identified and verified studies were incorporated into the Evidence Atlas. Supplementary Table S2 therefore contains two clearly separated parts: a documented search framework for the original search, and a complete prospective record for the update search. The principal direct genetic and genomic evidence for FPR resistance in cacao is summarised in Table 3.

The complementary evidence from clone-evaluation and breeding trials identified through the broadened search is summarised in Table 4.

Parallel searches combining *Moniliophthora perniciosa* or witches’ broom disease with the resistance concept block were used only to identify WBD comparator studies, such as the earliest WBD QTL studies retained as comparators [30,31], that required separation from the FPR literature. Backward and forward citation chaining was applied to every included study and to recent reviews of the pathogen and disease [3,32,33].

### 2.3. Eligibility Logic

Records were retained when they contributed primary evidence relevant to at least one of the following domains: FPR resistance or tolerance phenotyping, including clone-evaluation, progeny and multi-environment breeding trials; genetic architecture, heritability, QTL or association mapping; genomic prediction; host- or pathogen-side molecular responses to *M. roreri*; functional testing of resistance- or susceptibility-related candidates; or enabling genomic and breeding resources directly relevant to interpretation or deployment. WBD studies were retained only where they provided an explicit comparator, shared germplasm or datasets with FPR studies, or supported a clearly labelled cross-pathosystem hypothesis. Evidence from other cacao diseases or other plant–pathogen systems was not treated as direct evidence for FPR and was used only when explicitly identified as biological or technological extrapolation.

Classification as FPR-specific, WBD-specific or cross-pathosystem evidence was determined by the pathogen and phenotype actually evaluated in the primary study, rather than by generalised terminology in titles, abstracts or secondary literature.

### 2.4. Targeted Primary-Source Verification

A final targeted verification pass was conducted to strengthen the traceability of the core evidence base. This step focused on recovering primary-source information for the principal FPR mapping and molecular studies, including population identity, sample size, marker or QTL identifiers, genomic position, effect estimates where available, treatment of population structure and multiple testing, phenotyping design, heritability estimates, and evidence for independent validation.

This verification materially changed the interpretation of several prominent findings. It established that the Brown et al. [15] and Gutiérrez et al. [16] FPR QTL studies derive from the same F1 pedigree and therefore represent cross-study confirmation within one pedigree rather than independent replication; that the GWAS panel and the linkage family analysed by Romero Navarro et al. [17] are separate datasets within one publication, and that the linkage family shares the Pound 7 parent with the Brown–Gutiérrez population; that Ahn et al. [22] re-analysed the Osorio-Guarín et al. [21] panel using machine learning variable importance rather than a conventional mixed-model GWAS; that the three candidate effectors functionally tested by Nascimento et al. [34] were applied as recombinant proteins to tomato (Micro-Tom) seedlings rather than to cacao; and that MrB1 activity was demonstrated by exogenous application of the recombinant protein to *Nicotiana benthamiana* cells and to cacao leaves and fruits, without genetic manipulation of *M. roreri* [35]. These corrections are incorporated directly into Table 3, Table 5 and Table 6 and Supplementary Tables S1A and S3 rather than treated as peripheral caveats.

This verification was bounded rather than exhaustive. The Evidence Atlas should therefore be interpreted as a critically curated synthesis of the evidence retrieved and verified by the stated cut-off date, not as a claim of complete enumeration of every publication ever produced on FPR biology.

### 2.5. Interpreting Absence of Evidence

Statements such as “no functional validation was identified” or “no independently replicated FPR locus was identified” refer specifically to the literature retrieved and verified through the documented search strategy, including the original search completed on 30 August 2026 and the complementary update search completed on 1 October 2026. They represent an absence of evidence within the retrieved and verified literature and should not be interpreted as evidence of absence. Where potentially relevant records could not be verified against a primary source to the standard required for inclusion, they were not used to support substantive claims; such leads are listed in Supplementary Table S4 with the reason for exclusion or incomplete verification. This phrasing is applied consistently to every negative conclusion in the review, including those concerning E2b, E4 and E5.

### 2.6. A Review-Specific, Multidimensional Evidence Classification System

To prevent findings with fundamentally different evidentiary statuses from being described interchangeably as “associated”, “validated” or “resistance genes”, we developed an evidence classification system for this review (Table 1). It is not an adaptation of an established evidence-grading instrument such as GRADE [43], not a risk-of-bias tool, and not a technology-readiness scale. Following the reviewers’ recommendation, it is explicitly presented as an evidence classification system rather than as a hierarchy: its codes (E0–E5) are retained as compact labels, but they are not points on a single continuous scale of evidentiary strength, because independent genetic replication, correlative molecular evidence, functional activity, causal manipulation and breeding utility answer fundamentally different questions. The system is therefore organised as a matrix of three evidence axes plus one directness qualifier, in the spirit of evidence maps [44], and each claim is classified on the axis to which it belongs. Ordering is meaningful only within an axis (E1 < E2a < E2b; E3a < E3b < E4); comparisons across axes, such as whether E2b is “stronger” than E3b, are not intended and are not made anywhere in this review.

Each class answers a specific question (Table 1). The directness qualifier (E0) asks whether the claim is supported by data from the cacao–*M. roreri* interaction. The genetic axis (E1, E2a, E2b) asks whether a locus–phenotype association has been reproduced, and in what material. The mechanistic axis (E3a, E3b, E4) asks how strongly a molecular determinant has been linked to FPR, from correlation through experimental perturbation to stable causal manipulation; following the reviewers’ suggestion, E3b is subdivided according to the type of perturbation and the biological context in which it was performed. The translational axis (E5) asks whether using the information has improved a breeding decision; predictive-validation scope is recorded as a descriptor on this axis rather than as a grade.

Two clarifications respond to specific ambiguities in the earlier version. First, E2a does not imply independent replication. It includes any confirmation in material sharing individuals, parents or a breeding lineage with the discovery population, because linkage disequilibrium, allele frequencies and phenotyping conventions are then partly shared; only E2b, defined by documented genetic independence, can support claims of transferability. Second, E3b establishes that a molecule is active, or that its perturbation modulates host responses; it does not establish that the corresponding gene is required for virulence, or that its host target determines resistance during natural infection. Biochemical or cellular activity (E3b-i), host-response modulation (E3b-ii) and an experimentally demonstrated contribution to disease (E3b-iii, and ultimately E4) are therefore distinguished explicitly [45].

Because FPR is a disease of developing pods, the operational definition of E4 is pathosystem-specific. Strong causal evidence does not require whole-plant phenotyping, as the earlier formulation implied; it requires stable genetic manipulation combined with a reproducible change in a defined disease component when cacao pods are challenged with *M. roreri*. For host manipulation, this implies stably transformed or edited trees, or their progeny, old enough to set pods [41]; for pathogen manipulation, it implies stable mutant and, ideally, complemented *M. roreri* strains inoculated onto pods of defined age.

The classification is applied claim by claim rather than paper by paper: a single publication may support different evidence levels for different statements. Likewise, evidence obtained against WBD, *Phytophthora spp.* or another pathosystem retains its own class for that system but is classified as E0 when invoked as evidence for FPR without direct *M. roreri* testing.

Classes were assigned at the claim level using the operational criteria in Table 1, and the robustness descriptors listed beneath it. Class assignment was performed iteratively by the authors, and prospectively planned independent duplicate grading was not implemented; for the reasons given in Section 2.1, a retrospectively generated agreement statistic is not reported, because it would measure a re-reading performed after the final decisions were known rather than the review process actually conducted. Ambiguous cases were re-examined against the primary source and classified conservatively, at the lowest class directly supported by the evidence. A quantitative meta-analysis was not attempted because the retained FPR studies differed substantially in host populations, pathogen material, inoculation systems, disease endpoints, temporal structure and reported effect metrics, precluding biologically defensible statistical pooling.

## 3. The Pathogen and the Phenotype

Understanding the genetic architecture of frosty pod rot (FPR) resistance requires simultaneous consideration of the pathogen and of how the host phenotype is measured. In *Moniliophthora roreri*, predominantly clonal but geographically structured pathogen populations, an extended hemibiotrophic infection cycle, marked isolate-level variation in aggressiveness, strong dependence of disease development on fruit age and environment, and heterogeneous phenotyping procedures across studies can all influence estimates of resistance and the reproducibility of genetic associations. These features are therefore not merely epidemiological background; they define the biological context within which QTL, GWAS and genomic-prediction results must be interpreted.

### 3.1. Taxonomy and Reproductive Biology of Moniliophthora Roreri

*Moniliophthora roreri* (Cif.) H.C. Evans, Stalpers, Samson & Benny is a basidiomycete belonging to the Agaricomycetes, Agaricales and Marasmiaceae. Multilocus phylogenetic analyses established *M. roreri* and *M. perniciosa*, the causal agents of witches’ broom disease (WBD), as closely related taxa forming a distinct lineage within the Marasmiaceae [46]. Their reproductive biology, however, differs substantially. Whereas *M. perniciosa* forms basidiocarps and produces basidiospores, no conventional sexual fruiting body has been demonstrated for *M. roreri*. Instead, infected cacao pods produce massive quantities of asexual propagules directly on the necrotic fruit surface. Cytological and germination analyses have established these propagules as thallic, rhexolytic conidia generated through fragmentation of conidiogenous hyphae; conidia and hyphal cells may contain one or two nuclei, rarely more, and binucleate conidia tend to be larger than mononucleate ones [47]. Accordingly, descriptions of FPR epidemiology and artificial-inoculation experiments should refer to conidia rather than basidiospores or meiospores.

The reproductive biology of *M. roreri* is nevertheless more complex than complete loss of ancestral sexual machinery. The species retains the canonical basidiomycete mating-type loci: the A locus contains homeodomain genes, whereas the B locus contains pheromone and pheromone-receptor genes. Population-genetic and mating-type analyses identified biallelic A and B loci and predominant mating-type combinations, while strong multilocus linkage disequilibrium-supported predominantly clonal reproduction and reconstruction of the ancestral state was consistent with a heterothallic, tetrapolar mating system [48]. More recent comparative genomics substantially expanded this picture, identifying five A-locus alleles and three B-locus alleles occurring in eight allelic combinations, with several of the newly recognised combinations concentrated in Colombian isolates [49]. Genomic patterns are compatible with recombination having occurred during the evolutionary history of the species, but they do not demonstrate a contemporary sexual cycle. No sexual fruiting structure has been observed, and population-genetic and genomic evidence continues to support *M. roreri* as an essentially asexual, haploid and clonally propagating pathogen [48,49,50,51]. The presence or diversity of mating-type alleles should therefore not be interpreted as evidence of ongoing sexual reproduction.

The infection cycle is hemibiotrophic and includes an unusually extended biotrophic phase. After conidial germination and penetration of susceptible young pods, fungal growth develops predominantly within host tissues while external symptoms may remain absent or be limited to malformations. This phase can persist for approximately 45 days or longer, depending on host genotype, fruit developmental stage and environmental conditions [38,49]. The subsequent transition to necrotrophy is associated with rapid tissue destruction, increasingly invasive fungal growth and, ultimately, massive conidial production on the pod surface [32,38]. Genome, secretome and comparative-genomic evidence further indicate that the two phases are biologically and molecularly distinct, with differential deployment of secreted proteins, candidate effectors and cell-wall-degrading enzymes [38,49].

This temporal separation between infection, symptom appearance, necrosis and sporulation has direct consequences for resistance phenotyping. Fruit age at inoculation, incubation period, time to external symptom development, internal tissue damage and time to sporulation represent biologically distinct components of the host–pathogen interaction. Collapsing them into a single endpoint score of “disease severity” can therefore obscure different underlying resistance mechanisms and reduce comparability among genetic studies.

### 3.2. Genetic Diversity, Dispersal and Aggressiveness: Lineage Is Not Pathotype

Population-genetic studies demonstrate a substantial geographic structure within *M. roreri*. SNP-, SSR- and sequence-based analyses have consistently identified particularly high genetic diversity in Colombia, including the Upper Magdalena Valley, whereas populations established towards the expanding margins of the pathogen’s range show stronger signatures of founder effects and clonal propagation [49,51]. Population-genomic data from Peru and neighbouring regions further indicate that the current distribution of diversity has been shaped by agricultural movement of infected material [52]. Comparative-genomic analyses further show that clonally propagating lineages can accumulate distinctive structural variants, chromosome rearrangements, duplications and differences in gene content [49]. The geographic range of FPR has expanded progressively from South America through Central America and Mexico, and reports from more recently colonised areas, including Jamaica and western Brazil, reinforce the continuing invasive potential of the pathogen [4,49].

Genetic differentiation, however, must not be equated automatically with differences in aggressiveness. Direct experimental evidence for this distinction is provided by Phillips-Mora et al. [8], who compared seven Colombian *M. roreri* isolates representing four major pathogen genetic groups against five diverse cacao genotypes under standardised artificial pod inoculation. Disease incidence, external severity and internal severity were evaluated, with internal severity proving particularly informative for discriminating host responses. Although the isolates differed phenotypically, the extensive genetic variation among them was not matched by an equivalent diversity in aggressiveness: five of the seven isolates were broadly highly aggressive across the host panel, whereas two produced lower internal and external severity. Conversely, the cacao clone ICS-95 expressed a comparatively resistant response against all seven isolates [8].

These results have two important implications. First, documented isolate-level variation in aggressiveness means that isolate identity matters when resistance phenotypes are compared across experiments. Second, the molecular genetic group or lineage does not by itself predict aggressiveness. We therefore use aggressiveness to denote quantitative differences in the amount or severity of disease caused by an isolate under defined conditions, and reserve pathotype or physiological race for a reproducible pattern of differential pathogenicity across a defined set of host genotypes. The available evidence does not justify assigning every molecular lineage of *M. roreri* to a distinct pathotype, nor does it establish a stable one-to-one correspondence between genomic lineage and aggressiveness phenotype.

This distinction is particularly important for breeding and genomic prediction. A resistance source that performs well against one isolate or one geographic population should not automatically be assumed to provide equivalent protection against the pathogen diversity represented across the Neotropics. Conversely, the presence of genetically divergent pathogen populations does not, without phenotypic testing, imply that each poses a functionally distinct resistance challenge. Future multi-environment resistance trials should therefore incorporate, where practicable and phytosanitary regulations permit, multiple well-characterised isolates or geographically representative inoculum sources and explicitly analyse host genotype × pathogen isolate × environment interactions.

Environmental and crop-structural factors further modify the expression of FPR. Studies in cacao agroforestry systems in Costa Rica and in major production areas of Santander, Colombia, have shown that the canopy structure, shade-tree spatial arrangement, tree spacing, fruit availability and local environmental conditions can alter disease incidence and epidemic development [53,54], and microclimatic variables modulate the onset of symptoms and sporulation [55]. Genetic resistance must consequently be interpreted as one component of integrated disease management rather than as a standalone property independent of the pathogen population and production environment.

### 3.3. Economic Significance and Implications for Breeding

The economic impact of FPR is highly context-dependent. Losses vary with host susceptibility, inoculum pressure, environment and management intensity, and losses exceeding 80–90% of pods have been reported under highly conducive or poorly managed conditions [33]; such figures are not universal expected loss rates. Because sanitation, pod removal and canopy management require recurrent labour [3,5], a genotype that reduces disease can create value both through marketable yield and through reduced dependence on intensive management, which is particularly relevant in smallholder systems.

Resistance, however, cannot be pursued independently of productivity and bean quality. Cacao value-chain studies demonstrate that the genotype, specialty-market identity, postharvest practices and quality attributes materially affect the price received for cacao [56]. A breeding strategy that improves the FPR response while substantially reducing the yield, bean quality or fine-flavour characteristics could therefore generate little or no net genetic or economic gain. The relevant breeding objective is consequently multi-trait: reduced disease impact must be combined with acceptable productivity, adaptation and market-valued quality. This resistance × productivity × quality tension is addressed through multi-trait selection in Section 7.4.

### 3.4. A Proposed Phenotyping and Reporting Guideline for FPR Genetic Studies (PRG-FPR)

The primary genetic studies synthesised in this review do not share a common FPR phenotyping protocol. They differ in fruit developmental stage, inoculum concentration and identity, artificial versus natural infection, experimental unit, disease-rating scale, assessment timing and the distinction between external and internal disease severity. As shown comparatively in Table 6, this heterogeneity limits direct comparison of effect estimates among studies and is likely to contribute to the difficulty of replicating QTL and GWAS signals across populations.

This limitation is especially important because the phenotype is the foundation of every subsequent genomic inference: a higher marker density or more sophisticated models cannot compensate for a resistance phenotype that is biologically inconsistent or poorly documented. We therefore propose a Phenotyping and Reporting Guideline for FPR genetic studies (PRG-FPR; Table 2). The designation “Minimum Phenotyping Standard” used in the earlier version overstated its status. The guideline has not been validated by inter-laboratory or multi-site testing and is offered as a reporting checklist and design aid, comparable in intent to minimum-information guidelines in other fields, rather than as a standard. Its purpose is to ensure that studies report enough information for another investigator to judge whether two FPR phenotypes are biologically and statistically comparable.

PRG-FPR is organised in three modules. Module A contains general reporting items that apply to every FPR genetic study, whatever the source of infection. Module B applies only to controlled-challenge experiments and covers inoculum, pod developmental stage, inoculation and incubation conditions. Module C applies only to field and natural-infection studies and covers the epidemic context, pod-counting protocol and productivity denominator. This separation corrects a limitation of the earlier version, in which controlled-inoculation parameters were presented alongside general reporting items although they cannot be transferred unchanged to natural-infection studies. Module A also makes explicit features that are frequently under-reported: the hierarchical structure of experimental units (pods nested within trees, within clones, within blocks, sites and years), the unit of replication used to test genotype effects, scorer reliability, the treatment of repeated measurements and the statistical model. Treating pods as independent replicates when genotypes are replicated at the level of trees or plots is pseudoreplication [9] and inflates the apparent precision of genotype estimates. Numerical values such as inoculum concentration, pod age or assessment time are deliberately not prescribed, because no inter-laboratory evidence identifies values that are optimal across host genotypes, isolates and environments.

**PRG-FPR quick-reference checklist.**

For rapid appraisal and transparent reporting of FPR phenotyping studies, the following core information should be stated explicitly:

**Host material:** genotype or clone identity, origin or pedigree where known, propagation method and plant age.

**Pathogen context:** *M. roreri* isolate or inoculum source, provenance and identity where available.

**Pod developmental stage:** physiological age or an equivalent reproducible descriptor at inoculation or assessment.

**Inoculum and challenge conditions:** inoculum concentration, viability, inoculation method and relevant incubation conditions for controlled-challenge studies.

**Phenotype definition:** disease component measured, including incidence, external severity, internal severity, incubation period, sporulation or disease progress; scoring scale and assessment timing.

**Experimental design:** unit of observation, unit of replication, hierarchical nesting, randomisation, blocking and number of biological units.

**Scoring reliability:** scorer training, blinding where feasible and inter- or intra-rater reliability when visual scales are used.

**Environmental and epidemic context:** temperature, relative humidity and challenge environment for controlled studies, or site, season, disease pressure and management context for field studies.

**Statistical analysis:** response distribution, link function, random effects, treatment of repeated measurements and the scale on which heritability or repeatability is reported.

**Data traceability:** pod-, tree- or plot-level data and associated metadata, where feasible.

This quick-reference checklist is a practical summary of Table 2 and does not replace the complete PRG-FPR modules or constitute a validated standard.

PRG-FPR is deliberately presented as a review-derived proposal, not as an externally validated consensus protocol. Its individual elements are nevertheless grounded in the biological features of the *M. roreri* infection cycle and in methodological differences documented across the primary genetic studies reviewed here. Its value should ultimately be tested empirically through multi-site experiments in which common cacao genotypes and, where quarantine regulations permit, well-characterised pathogen isolates are evaluated under harmonised protocols.

A particularly important priority is the establishment of shared reference materials. The controlled multi-isolate study of Phillips-Mora et al. [8] showed that some cacao genotypes can maintain comparatively consistent responses across genetically diverse isolates, but the current evidence is insufficient to nominate a single universal susceptible or resistant differential clone for all FPR research programmes. A coordinated inter-laboratory study using common host genotypes, common scoring scales and traceable pathogen isolates would therefore provide the experimental basis needed to move PRG-FPR from a proposed reporting guideline towards a validated, consensus protocol.

Until such harmonisation is achieved, apparent failure to replicate a resistance-associated locus across studies should not automatically be interpreted as evidence that the original locus was false. It may reflect a genuinely population-specific genetic effect, but it may also arise because the underlying phenotype differed in pathogen isolate, pod developmental stage, inoculum dose, disease component measured or environmental context. This phenotypic uncertainty is carried explicitly into the interpretation of the genetic Evidence Atlas in Section 4.

### 3.5. Statistical Treatment of Incidence, Severity, Ratio and Index Phenotypes

Most FPR phenotypes in the genetic literature are proportions, ordinal scores, times to an event or composite indices, and their statistical properties affect heritability estimates, genetic correlations and genomic predictions. Four issues deserve explicit attention.

First, incidence is a binomial outcome. The proportion of diseased pods per tree has a variance that depends on its mean and on the number of pods observed, and that number varies among genotypes, trees and years. Analysing arcsine- or logit-transformed proportions with linear models ignores this unequal information content; generalised linear mixed models with a binomial distribution (or a beta-binomial distribution or observation-level random effect when data are overdispersed), using the numbers of diseased and total pods per tree and period as the response, are generally preferable [57,58]. Genotype effects should be tested against variation among trees within genotypes, and repeated assessments of the same tree should be modelled with tree-level random effects or an appropriate temporal covariance structure.

Second, incidence is a ratio whose denominator is itself a productivity trait. Genotypes that produce more pods, or produce them when inoculum pressure is low, may show lower incidence for reasons unrelated to resistance, and correlations between incidence and yield partly reflect the shared denominator, a classical source of spurious correlation between ratio variables. Numerators (diseased pods) and denominators (total pods) should therefore be reported separately, and models that analyse diseased pods conditionally on total pods, with fruiting phenology as a covariate where available, help to separate resistance from escape and productivity effects. The same caution applies, more strongly, to composite indices. Disease and production indices that combine total pod production with the ratio of diseased to healthy pods [25,26] are useful for ranking clones against a combined objective, but they embed an implicit weighting of productivity and disease, are scale-dependent and cannot be decomposed into genetic components without the underlying data. For genetic analysis, the components should be modelled jointly, for example, with multivariate mixed models, and any index constructed afterwards from component predictions with explicit weights (Section 7.4).

Third, severity scales are ordinal. External and internal severity are usually scored in classes whose intervals are not biologically equal, and averaging class values treats them as interval data, which can bias comparisons [10]. Cumulative-link (ordinal) mixed models, or analysis of the proportion of pods exceeding biologically meaningful classes, represent such data more faithfully. The incubation period and time to sporulation are time-to-event variables in which pods that are harvested, lost or still asymptomatic at the last assessment are right-censored; survival models handle this censoring explicitly [59], whereas averaging observed days discards it. Disease progress over repeated assessments can be summarised by the area under the disease progress curve or, when assessments are sparse, by the area under the disease progress stairs [60].

Fourth, heritability is defined only relative to a scale, a unit of selection and a population. For binary and proportion traits, heritability differs between the observed and the latent (liability) scale [61]; clone-mean (entry-mean) heritability, which increases with the number of trees, years and sites per clone, is not comparable with individual-tree narrow-sense heritability [62]; and for unbalanced trials, a generalised definition based on prediction error variance is more appropriate than the classical variance ratio [63]. Estimates are also conditional on the health and management status of the material, which is rarely reported. In a cacao diallel evaluated for foliar resistance to *Phytophthora palmivora* and *P. megakarya*, infection of the plants by cacao swollen shoot virus altered the mean squares for general and specific combining ability and raised narrow-sense heritability from 0.31 to 0.36–0.43 depending on the virus strain and pathogen [7]. That study concerns another pathosystem and another organ, and is therefore E0 for FPR; it is cited here only as a documented demonstration that an unrelated biotic factor can silently change estimated genetic parameters, which is why Module A of PRG-FPR asks for the health and management status of the evaluated material to be reported. These distinctions matter for the FPR literature: the broad-sense clonal heritability of 0.73–0.79 reported for the proportion of diseased pods in a Costa Rican clonal panel [17] and the individual-tree narrow-sense heritability of approximately 0.36–0.46 estimated in Mexican full-sib progenies [12,24] measure different quantities, so they should be neither read as contradictory nor pooled as a single range. Scorer reliability should be quantified on a calibration subset of pods, with scorers blind to genotype where feasible. These recommendations, summarised in Module A of Table 2, are standard practice in quantitative genetics and plant epidemiology rather than innovations of this review; their value lies in making FPR phenotypes comparable and fit for genetic inference.

## 4. Genetic Architecture of FPR Resistance: The Evidence Atlas

### 4.1. Terminology: Resistance, Tolerance, and What a Phenotype Can and Cannot Distinguish

Cacao genetic diversity, structured into at least ten major ancestral genetic groups—Marañón, Curaray, Criollo, Iquitos, Nanay, Contamana, Amelonado, Purús, Nacional and Guiana—provides the broad germplasm base from which sources of reduced susceptibility to frosty pod rot (FPR) have been identified and incorporated into breeding populations [2]. Regional improvement programmes have subsequently combined diverse germplasm sources to generate clones and families differing markedly in their response to *Moniliophthora roreri* [4,17]. Among the genotypes repeatedly used to investigate the molecular basis of this response, UF-273, CATIE-R7 and CATIE-R4 have been consistently described in the primary FPR literature as tolerant, whereas Pound 7 and CATIE-1000 have served as susceptible comparators [36,37]. CATIE-R4 is a breeding-derived genotype—an offspring of UF-273 × PA-169—rather than a wild Amazonian accession [36,37].

Terminological precision is important because resistance and tolerance are not biologically interchangeable [64]. In this review, **resistance** denotes a host response that reduces infection, pathogen colonisation, reproduction or disease development; **partial (quantitative) resistance** denotes resistance that reduces but does not prevent disease and is typically polygenic [65,66]; **tolerance** denotes the capacity of an infected host to limit the yield penalty caused by a given pathogen burden, which can only be demonstrated by measuring productivity at a comparable level of infection; and **reduced susceptibility** is used as an umbrella term when the phenotype cannot discriminate among these mechanisms. The FPR literature does not always separate these components, and several studies classify clones as “tolerant” from symptoms or the comparative field response rather than from the yield maintained at an equivalent pathogen load [36,37]. We therefore report the primary study’s terminology in quotation marks when describing its results, and otherwise use partial resistance, reduced susceptibility or reduced disease severity. These conventions are applied consistently in Table 3, Table 4, Table 5 and Table 6.

This distinction directly constrains the interpretation of molecular candidates. A locus associated with lower external severity, for example, cannot automatically be described as a gene controlling pathogen recognition or colonisation; the phenotype could instead reflect delayed symptom development, restricted tissue damage, altered pathogen reproduction or greater host tolerance after infection. Similarly, incidence, external severity, internal severity, incubation period, sporulation and productivity under disease pressure represent related but biologically non-equivalent components of the host response. This is why the phenotypic definitions introduced in PRG-FPR (Table 2) are carried explicitly into the Evidence Atlas rather than treating all measurements simply as “FPR resistance”.

The mechanistic evidence base is also broader than a single reference genotype, although some germplasm is disproportionately represented. The matched host- and pathogen-side studies of Ali et al. [36] and Bailey et al. [37] compared multiple tolerant and susceptible clones, including UF-273, CATIE-R7, CATIE-R4, Pound 7 and CATIE-1000, whereas subsequent transcriptome-based modelling has incorporated additional contrasting cacao backgrounds [39]. CATIE-R4 therefore remains an important reference for studying partial resistance/tolerance, but mechanisms detected in this genotype should not be assumed to represent the entire genetic diversity of cacao without independent confirmation in additional backgrounds.

Across the FPR-specific linkage, association and quantitative-genetic studies retained in this review (Table 3; Supplementary Table S1A), no major qualitative R/Avr-type resistance gene has yet been demonstrated. Instead, the available evidence is dominated by multiple QTL or marker-associated signals of moderate or small effect, population-dependent heritability estimates and incomplete resistance phenotypes. Taken together, these findings support a quantitative and partial genetic architecture of FPR resistance, rather than a simple monogenic model, consistent with the general pattern of quantitative disease resistance in plants [65,66]. This distinction has direct consequences for breeding: individual QTL or candidate genes may contribute useful information, but the present evidence does not support treating any single locus as a universally predictive resistance determinant.

### 4.2. Direct Genetic and Genomic Evidence

The genetic evidence for frosty pod rot resistance is considerably smaller than the number of loci and candidate genes reported across the broader *Moniliophthora* literature might suggest. To preserve transparency without overwhelming the main text, the complete Evidence Atlas—including population identity, reference genome, marker or QTL identifiers, genomic coordinates, effect estimates, population-structure correction, validation status, evidence grade and DOI—is provided in Supplementary Table S1A, together with the relatedness among the populations studied. Table 3 distils the principal FPR-specific findings required to assess the current maturity of the field.

Three conclusions emerge from Table 3. First, the Brown–Gutiérrez series provides the strongest locus-level evidence currently available for FPR, but its ceiling is E2a because re-detection and refinement occurred within the same pedigree [15,16]. Second, broader germplasm panels expand the catalogue of potentially useful loci but remain discovery-stage evidence. Romero Navarro et al. [17] identified six Bonferroni-significant FPR-associated SNPs in 148 improved clones; in the same publication, a separate linkage analysis of a small Pound 7 × UF273 family detected a chromosome-5 QTL explaining 28.8% of phenotypic variation. Because that family shares the Pound 7 parent with the Brown–Gutiérrez population and contains only 68 individuals, its estimated effect is probably inflated [20], and it cannot provide independent confirmation of the Brown–Gutiérrez loci. The chromosome-1 signal reported by Osorio-Guarín et al. [21] requires greater caution because the nominal association did not survive false-discovery-rate correction. Third, computational re-analysis does not constitute biological replication: Ahn et al. [22] re-used the same 102-accession dataset and ranked genomic features by machine learning variable importance, which is useful for hypothesis generation but neither reproduces the original association nor provides a significance test.

The absence of E2b evidence is not explained solely by a lack of cross-population testing. McElroy et al. [23] evaluated three related Ecuadorian populations derived from overlapping parents; FPR-associated markers were largely population-specific, and when a GBLUP model trained in one population was applied to the other two, the mean correlation between predicted and observed monilia-pod incidence was approximately 0.16. Because these populations are related, this is external validation in partially dependent material, and the low transferability is therefore all the more informative: population-specific genetic architecture, differences in linkage disequilibrium and phenotypic non-equivalence remain major obstacles to the operational use of FPR markers and predictions.

The quantitative-genetic evidence nevertheless shows that the FPR response is heritable and therefore selectable. The values differ in definition as well as in magnitude: a broad-sense clonal heritability of 0.73–0.79 for the proportion of diseased pods in the Costa Rican clonal panel [17], and individual-tree narrow-sense heritability of approximately 0.36–0.46 in Mexican full-sib progenies [12,24] (Section 3.5). Taken together, the Atlas shows that the principal limitation of FPR genetics is no longer the absence of candidate loci, but the absence of independent replication under sufficiently comparable phenotyping conditions.

### 4.3. Clone-Evaluation and Breeding-Trial Evidence

The broadened search identified a body of clone-evaluation, progeny and multi-environment trials that do not report loci or molecular markers but are directly relevant to genetic gain (Table 4). Their principal contributions are threefold. First, they document exploitable genetic variation for the FPR response among clones under both artificial inoculation and natural infection [8,26,27]. Second, they show that the most productive clones are not necessarily the least diseased, so the disease response and productivity must be evaluated jointly [25,26]; several programmes have responded by adopting composite disease and production indices, whose statistical properties are discussed in Section 3.5. Regional surveys add a complementary, if weaker, line of evidence: in four farms in Norte de Santander, Colombia, FPR incidence ranged from 0.4 to 21.6%, with hybrid material most affected and the lowest incidence in CCN 51, ICS 95 and IMC 67 [29]. The low incidence of ICS 95 is notable because the same clone expressed a comparatively resistant response against all seven isolates tested under controlled inoculation by Phillips-Mora et al. [8], so two independent studies using different designs, pathogen materials and countries converge on this genotype. That convergence should nonetheless be read cautiously because, in the survey, biological material was confounded with farm; if no replication structure or genotype-level statistical test was reported; and clone designations were given inconsistently within the source—precisely the reporting gaps that Module A of PRG-FPR is intended to close. Third, they document that clone performance varies with the production system and environment [27,28], which supports the multi-environment validation emphasised in Section 7 and Section 9. Regional trial networks such as CacaoFIT, which assembles more than 25 field trials and over 150 genotypes across Latin America, provide infrastructure in which such validation could be coordinated [67].

Under the classification system, these studies support claims about phenotypic variation and clonal performance rather than locus- or mechanism-level claims, and they are therefore summarised without E-codes. They also show, importantly, that phenotypic clonal selection has already delivered deployable clones with reduced FPR, such as the CATIE-derived clones used as tolerant references in the molecular literature [17,36]. This is the operational baseline against which any molecular tool must demonstrate added value (Section 9).

### 4.4. Candidate and Mechanistic Evidence: From Positional Signals to Direct Molecular Support

The term “candidate gene” encompasses findings with very different evidentiary statuses in the FPR literature. Some candidates are genes located near a GWAS or QTL signal; others emerge from direct host- or pathogen-side transcriptomic studies; still others are mechanistically attractive genes validated against a different cacao pathogen but never tested against *M. roreri*. Treating these categories as equivalent would obscure the central distinction made by the evidence classification system in Table 1. We therefore summarise representative candidates and molecular evidence in Table 5, while the complete gene-level matrix—including gene identifiers, annotation, pathways, molecular evidence, functional evidence, pleiotropy considerations and claim-specific E0–E5 classification—is provided in Supplementary Table S3.

Four evidentiary strata emerge from Table 5. Positional and computational candidates derived from association analyses [17,21,22] remain E1, because statistical proximity or feature importance does not show that the nominated gene participates in the FPR response. In Romero Navarro et al. [17], for example, expression differences between UF273 and Pound 7 were attributable mainly to the cultivar rather than to inoculation, providing context around contrasting germplasms rather than evidence of infection-induced resistance mechanisms. Direct but correlative molecular evidence from the cacao–*M. roreri* interaction forms the second stratum (E3a): tolerance-dependent host transcriptional responses involving jasmonate–ethylene and salicylate signalling, brassinosteroid and abscisic-acid metabolism [36], and extensive host-background-dependent reprogramming of the pathogen transcriptome [37], complemented by genome/secretome and network analyses [38,39].

The third stratum comprises functional-activity evidence for individual pathogen effectors (E3b-i). The verification undertaken for this revision showed that this evidence is weaker, in FPR-specific terms, than described in the earlier version. The three candidate effectors characterised by Nascimento et al. [34] were produced as recombinant proteins and sprayed onto tomato seedlings; the necrosis and wilting observed therefore establish biological activity in a heterologous plant, not in cacao. MrB1 was tested more directly in the relevant host: the recombinant protein caused necrosis in cacao leaves, with differences among cacao genotypes, and localised necrosis in cacao fruits [35]. Both studies demonstrate biochemical or cellular activity; neither shows that the corresponding gene is required for virulence during infection, that natural variation in host response to the protein explains field resistance, or that the protein is delivered at active concentrations during natural infection [45]. The fourth stratum comprises cross-pathosystem candidates such as MLO and TcNPR3, which remain E0 for FPR because neither has been tested against *M. roreri* [21,41,42].

Taken together, the candidate and mechanistic literature reveals an asymmetric frontier. Host-side FPR evidence reaches E3a; pathogen-side evidence reaches E3b-i, in cacao tissues only for MrB1. No FPR-specific host or pathogen determinant identified in the literature retrieved has been perturbed during infection of cacao pods (E3b-iii) or stably manipulated with a resulting change in an FPR disease component (E4). The most informative next experiments are therefore those that test whether perturbing prioritised host determinants, or genetically disrupting candidate effectors, alters infection establishment, colonisation, symptom development, internal damage or sporulation during infection of pods, ideally in more than one host genotype and with more than one isolate.

### 4.5. Phenotyping Heterogeneity Across the Primary Genetic Literature

The genetic Evidence Atlas cannot be interpreted independently of the phenotypes on which it is built. The principal FPR studies differ not only in germplasm and analytical method but also in how infection was generated, which component of disease was measured, how frequently pods were assessed and over what period observations were integrated. Table 6 therefore compares the phenotyping designs underlying the genetic and genomic evidence summarised in Table 3. Epidemiological studies that did not generate genetic parameters or marker associations are not included here, although they remain informative for defining environmentally relevant components of PRG-FPR (Section 3.4).

The contrast between the controlled mapping studies and the field-based genomic studies is particularly informative. Gutiérrez et al. [16] evaluated attached pods of a defined developmental stage, used repeated artificial inoculation events and measured both external and internal severity nine weeks after challenge. By contrast, Osorio-Guarín et al. [21], Romero Navarro et al. [17], McElroy et al. [23] and Solís Bonilla et al. [12,24] relied on disease expression under field exposure, integrating naturally varying inocula, environmental conditions and host developmental states. Neither approach is intrinsically superior: controlled inoculation improves biological standardisation and attribution to the challenged pathogen, whereas repeated field phenotyping captures the environmental variability under which breeding material must ultimately perform.

The studies also measure different biological components of FPR response. Brown et al. [15] and Gutiérrez et al. [16] distinguish external and internal pod damage; Osorio-Guarín et al. [21] summarise repeated disease observations through AUDPC; Romero Navarro et al. [17] estimate the proportion of diseased pods over an eight-year trial; McElroy et al. [23] use the field-derived monilia pod incidence across three related Ecuadorian breeding populations; and Solís Bonilla et al. [12,24] evaluate the incidence relative to total pod production. These variables are correlated manifestations of disease but are not interchangeable phenotypes, and they differ in their statistical properties (Section 3.5). A locus affecting infection probability, for example, need not have the same effect on internal necrosis, the rate of symptom progression or productivity under chronic disease pressure.

An additional lesson emerges from the Mexican trials. Solís Bonilla et al. [12] demonstrated significant spatial autocorrelation in both FPR incidence and productivity and showed that autoregressive and Bayesian spatial models altered the estimation of genetic parameters relative to simpler approaches. This finding reinforces a broader point: in perennial field trials, phenotypic standardisation must encompass not only the disease-assessment scale but also the spatial structure of the experiment. Failure to model local environmental heterogeneity can transfer non-genetic spatial variation into estimated breeding values or heritability.

Taken together, Table 6 shows that phenotypic non-equivalence is a plausible contributor to the limited replication of FPR-associated loci across studies. It should not, however, be invoked as an automatic explanation for every failed replication. A genomic signal may genuinely be population-specific or false. The appropriate response is therefore prospective harmonisation: future independent-validation studies should retain the advantages of multi-year field evaluation while reporting, and where experimentally feasible standardising, the core variables defined by PRG-FPR (Table 2).

For this reason, the most informative next generation of FPR genetic studies would combine three features that have so far appeared largely separately in the literature: standardised pathogen challenge and disease-component scoring, genetically independent validation populations, and multi-environment or spatially explicit field evaluation. Such designs would allow failure to replicate a locus to be interpreted biologically rather than remaining confounded with differences in phenotyping methodology.

### 4.6. Evidence Landscape for FPR Resistance in Cacao

Table 3, Table 4 and Table 5 resolve the evidence at the level of individual studies, loci and molecular findings. Figure 1 summarises how that evidence is distributed across the axes of the classification system. Because the axes answer different questions, the figure should not be read as a pipeline through which every locus or candidate must progress: direct molecular evidence can reach E3a or E3b without any associated locus having reached E2b, and genomic prediction can progress towards E5 without a causal gene.

The landscape shows two distinct bottlenecks. On the genetic axis, evidence is concentrated at E1, the strongest locus-level support reaches E2a within one pedigree [15,16], and the only cross-population test available showed poor transferability [23]. On the mechanistic axis, host evidence is correlative (E3a), pathogen evidence reaches functional activity (E3b-i), and the step from activity to a demonstrated contribution to disease (E3b-iii or E4) has not been taken. On the translational axis, predictive validation is limited to random cross-validation within one panel and external validation among related populations, and no marker, model or intervention has reached E5.

## 5. Molecular Mechanisms: A Two-Genome, Temporally Staged Interaction

The molecular evidence for frosty pod rot should not be interpreted as a linear extension of the QTL and association evidence described in Section 4. Direct molecular studies can resolve host or pathogen processes without identifying the causal locus underlying a population-level resistance phenotype, and conversely, a statistically associated locus may remain mechanistically unresolved. This distinction is particularly important for *M. roreri*, a hemibiotrophic pathogen whose interaction with cacao changes markedly as infection progresses from symptomless colonisation towards tissue necrosis and sporulation [3,38]. We therefore organise the available evidence according to the biological stage and genome being interrogated, while retaining the claim-specific E0–E5 classification introduced in Table 1.

A second distinction is equally important. The available studies differ substantially in their temporal resolution: some directly compare host responses during successful infection, others profile fungal developmental stages or reanalyse infection-associated transcriptomes, and more recent studies test the activity of individual recombinant pathogen effectors outside the context of infection. Assignment of a molecular observation to a specific infection phase should therefore be regarded as biologically informed interpretation where the original experimental design did not itself resolve that phase temporally.

### 5.1. Host Defence Responses During Early Infection and the Biotrophic Phase

Direct host-side evidence is provided by Ali et al. [36], who compared gene expression in cacao clones differing in their level of tolerance following successful infection by *M. roreri*. The resulting transcriptional response implicated interconnected jasmonic-acid/ethylene- and salicylic-acid-associated defence programmes, together with changes involving brassinosteroid and abscisic-acid metabolism, phenolic accumulation and heat-shock-related responses. Because these molecular responses were measured directly in cacao tissues interacting with *M. roreri*, they constitute FPR-specific molecular evidence and are classified here as E3a rather than as cross-pathosystem extrapolation.

This distinction corrects a recurrent oversimplification in the broader Moniliophthora literature. Jasmonate- and salicylate-associated defence responses are therefore not merely inferred for FPR from witches’ broom disease; their involvement has been measured directly in the cacao–*M. roreri* interaction [36]. What remains unverified for FPR is the more specific claim that tolerant cacao activates the jasmonate pathway earlier and for longer than susceptible cacao. Earlier and more sustained activation of jasmonate-biosynthesis genes has been demonstrated in cacao challenged with *M. perniciosa* [68], but the Ali et al. [36] experiment does not provide an equivalent time-course comparison of pathway onset. The kinetic claim must consequently remain an E0 extrapolation for FPR even though involvement of the pathway itself is supported directly at E3a.

The available evidence should likewise not be interpreted as demonstrating that any individual host gene within these pathways controls resistance. Differential expression establishes molecular participation and provides candidate mechanisms, but it does not establish whether a transcript is causal, compensatory or simply downstream of differences in infection progression. No host gene emerging from these direct transcriptomic responses has yet been perturbationally demonstrated to alter the FPR outcome.

### 5.2. Pathogen Transcriptional Responses and Host-Genotype-Dependent Infection

The reciprocal pathogen-side component is unusually well represented by Bailey et al. [37], which was generated from the same broader field experiment as the host-side study of Ali et al. [36]. Bailey et al. identified extensive differential expression in *M. roreri* during infection of cacao clones differing in tolerance, including 873 fungal genes whose expression varied among host backgrounds, with selected transcripts independently examined by qRT-PCR [37]. The paired nature of these studies is important because it provides one of the few direct opportunities in the FPR literature to examine the same biological interaction from both genomes rather than interpreting host and pathogen datasets generated under unrelated conditions.

These observations provide E3a evidence that successful infection of contrasting cacao genotypes is accompanied by substantial reprogramming of the pathogen transcriptome. They do not, however, demonstrate that the differentially expressed fungal genes are individually required for virulence or for overcoming host tolerance. The distinction between an infection-associated transcriptional programme and a causal virulence mechanism therefore remains essential.

More recent network and machine learning analyses have extended this pathogen-side perspective. Lim et al. [39] analysed infection-associated *M. roreri* transcriptional data from contrasting cacao backgrounds and prioritised regulatory hubs including a methyltransferase and a small heat-shock-protein-related candidate. The host background influenced the behaviour and predictive performance of the resulting models, supporting the broader hypothesis that pathogen transcriptional regulation is conditioned by the cacao genotype [39]. Nevertheless, network centrality and machine learning importance remain computational prioritisation rather than functional perturbation. We therefore retain these findings as E3a, hypothesis-generating, rather than elevating them to causal evidence.

### 5.3. The Biotrophic-to-Necrotrophic Transition: Secreted Proteins and Effector Activity

Genome and secretome analyses provided the first broad molecular framework for the transition between the biotrophic and necrotrophic phases of *M. roreri*. Meinhardt et al. [38] identified a substantial repertoire of secreted proteins, candidate effectors and cell-wall-degrading enzymes, together with phase-associated expression patterns consistent with the progressive transition from symptomless colonisation to host-tissue degradation. This constitutes direct FPR molecular support at E3a at the functional-category level: the study establishes biologically relevant repertoires and phase-associated molecular programmes, but does not by itself demonstrate the activity of individual effector genes.

Nascimento et al. [34] profiled *M. roreri* spores and mycelium exposed to cacao fruit extract and identified 871 differentially expressed genes; among 1075 transcripts predicted to encode secreted proteins, 61 were differentially expressed and 23 were prioritised as putative effectors. Three candidates were produced as recombinant proteins in *Escherichia coli* and sprayed onto tomato (Micro-Tom) seedlings, where they caused leaf drying, wilting and, often, necrosis. This establishes the biological activity of individual FPR-derived proteins (E3b-i), but in a heterologous plant: it does not show activity in cacao, delivery during infection, or a requirement for virulence. The comparable necrosis-inducing activity of effector families in *M. perniciosa* [69] makes these candidates biologically plausible, but plausibility by analogy remains E0 for FPR.

MrB1, the *M. roreri* homologue of the *M. perniciosa* BASIDIN effector, provides more direct evidence in the relevant host [35]. Recombinant MrB1 caused swelling, plasma-membrane damage and electrolyte leakage in *N. benthamiana* cell suspensions and altered the abundance of proteins involved in cellular homeostasis; sprayed onto cacao leaves, it caused necrotic lesions whose extent differed among cacao genotypes, and it induced localised necrosis when applied to cacao fruits. These experiments provide E3b-i evidence for the activity of an individual *M. roreri* effector in cacao tissues. They do not establish a contribution to disease: MrB1 has not been genetically disrupted in *M. roreri* and followed through infection of pods, and no host determinant of MrB1 perception has been manipulated. MrB1 is therefore best described as a functionally characterised candidate effector and a potential probe for differential host responses, not yet as a causally validated virulence factor or breeding target.

### 5.4. Mechanistic Evidence Boundaries and Implications for Resistance Breeding

Taken together, the molecular literature now reveals a more mature but still discontinuous evidence landscape than earlier syntheses suggested. Direct host transcriptomics identifies hormone-, stress- and defence-associated responses during *M. roreri* infection [36]; matched pathogen transcriptomics demonstrates extensive host-background-dependent fungal transcriptional reprogramming [37]; genome, secretome and network analyses resolve candidate virulence processes and regulatory modules [38,39]; and recent effector studies demonstrate the biological activity of individual recombinant *M. roreri* proteins [34,35].

The crucial evidentiary gap therefore lies between molecular activity and disease. Pathogen-side evidence reaches E3b-i, but no host resistance or susceptibility gene and no pathogen effector has been perturbed during infection of cacao pods or stably manipulated with a resulting change in FPR outcome. An effector that causes necrosis when applied to cacao tissue is active, but this does not establish that natural allelic variation in the host response to that protein explains field resistance; similarly, a host transcript that is differentially expressed during infection is mechanistically relevant but not automatically a resistance gene. Bridging these levels requires experiments that connect pathogen perturbation, host genotype and infection phenotype in pods and, ultimately, independent genetic backgrounds.

Cross-pathosystem evidence should remain outside that direct chain. The MLO-like candidate identified for WBD [21], the earlier temporal behaviour of jasmonate biosynthesis observed during *M. perniciosa* infection [68], and the stable TcNPR3 evidence generated against *Phytophthora spp.* [41,42] remain biologically informative but do not substitute for FPR-specific validation. The resulting picture is therefore neither one of mechanistic ignorance nor one of deployment readiness: FPR now possesses direct molecular evidence on both interacting genomes and functional-activity evidence for individual pathogen effectors, but the transition from molecular activity to a demonstrated contribution to disease, and from there to breeding utility, remains unresolved.

## 6. From Association to Causality: Genomics, Multi-Omics and Evidence Integration

The preceding sections show that the central limitation in FPR research is no longer the absence of genomic resources or candidate hypotheses, but the difficulty of connecting those resources to reproducible phenotypes and ultimately to causal mechanisms. Reference genomes, pangenomes, genome-wide association analyses, machine learning approaches and multi-omic datasets can substantially expand the candidate space, but none of these technologies intrinsically converts association into causality. Their value for FPR therefore depends on how they are incorporated into the evidence chain defined in Table 1. In this section, we examine these enabling approaches specifically in terms of what they can contribute beyond the evidence already established in Section 4 and Section 5, while distinguishing genomic resolution from FPR-specific validation.

### 6.1. Structural Genomics, Reference Genomes and Pangenomics: Expanding the Searchable Variation Space

The availability of a high-quality *Theobroma cacao* reference genome, initially developed from the Criollo genotype B97-61/B2, fundamentally changed the resolution at which cacao genetic variation could be studied [1], and was followed by an improved Criollo assembly and by a reference for the widely cultivated Amelonado-type Matina 1–6 genotype [70]. Reference-based resources have subsequently supported higher-density linkage mapping, genome-wide association studies, candidate-gene localisation and genomic prediction. A single reference genome, however, necessarily represents only a subset of the structural and gene-content diversity present across the species. This limitation is particularly relevant to cacao because its genetic diversity is strongly structured geographically and substantial diversity persists in an Amazonian, wild, semi-domesticated and ex situ germplasm that is incompletely represented in many active breeding populations [2,71].

Pangenomics provides a potential route to capture variation that is poorly represented by a single linear reference. The cacao pangenome reported by Argout et al. was constructed from 216 accessions, including 185 de novo-sequenced genomes, and described 30,489 pan-genomic genes, including 1407 genes not previously annotated in the reference resource [72]. The study also reported domestication-associated changes in gene content, particularly in the Criollo germplasm. At the original search and verification cut-off of 30 August 2026, this study was available only as a bioRxiv preprint [72]. Its publication status was re-checked during the complementary update search completed on 1 October 2026, and no peer-reviewed version was identified. Its quantitative conclusions should therefore be interpreted with the corresponding evidentiary caution.

For FPR specifically, the significance of pangenomics lies not in the existence of a larger catalogue of genes but in the classes of genetic variation that can now be interrogated. These include presence/absence variation, copy number variation, larger structural variants and highly variable genomic regions that may be poorly tagged by SNPs represented in conventional reference-based analyses. Such variation could theoretically affect defence-related gene families, receptor-like kinases, nucleotide-binding leucine-rich-repeat proteins, signalling components or other loci relevant to host–pathogen interactions. Importantly, however, the cacao pangenome study did not establish that any of these classes of variation controls resistance to *M. roreri* [72]. Their relevance to FPR therefore remains a testable hypothesis rather than direct FPR evidence.

This distinction is important under the classification system used throughout this review. A structural variant, presence/absence variant or pangenomic gene becomes FPR-relevant evidence only when it is linked directly to a defined FPR phenotype. Identification of the variant in a pangenome is an enabling genomic observation; association with FPR in one population would constitute E1; replication in genetically independent material would be required for E2b; and molecular or perturbational experiments would be needed to support claims on the mechanistic axis (E3–E4). Pangenomics should therefore be viewed as a means of expanding the genomic search space and reducing reference bias, not as an independent validation mechanism.

The increasing availability of high-contiguity, haplotype-resolved assemblies strengthens this opportunity. The recent near-telomere-to-telomere assembly of the commercially important CCN51 genotype resolved two haplotypes of approximately 414.0 and 417.7 Mb and provides a substantially improved resource for characterising heterozygosity, repetitive regions and structural variation in an agronomically important cacao background [73]. Nevertheless, the genomic assembly itself does not demonstrate that any particular CCN51 allele or structural feature confers FPR resistance. The appropriate next step is therefore comparative rather than descriptive: resistant and susceptible germplasm representing genetically diverse cacao groups should be analysed jointly for SNP, indel, presence/absence, copy number and structural variation and then tested against phenotypes generated under the harmonised conditions proposed in PRG-FPR.

The strongest application of structural genomics to FPR would consequently be a design in which pangenome-aware variation is integrated with independent populations and standardised disease phenotypes, rather than simply re-analysing existing small panels against a more complete reference. Such an approach could determine whether previously unresolved structural variants explain part of the missing transferability of FPR associations observed across cacao populations. Until such evidence is generated, pangenomic and haplotype-resolved resources should be described as powerful enabling resources for FPR genetics, but not as evidence that specific structural variants or gene-content differences confer resistance.

### 6.2. GWAS and Machine Learning: Distinguishing Association, Prediction and Biological Evidence

Genome-wide association studies have expanded the catalogue of genomic regions potentially associated with FPR response, but their interpretation depends on statistical design, control of population structure and relatedness, multiple-testing control, phenotype quality and independent replication. The direct FPR literature (Section 4.2) illustrates that discovery within one population and transferability across populations are distinct evidentiary questions.

Machine learning analyses add a further distinction, and the two should be treated as different types of analytical evidence. A GWAS tests, marker by marker, a null hypothesis of no association under a model that accounts for structure and relatedness, and yields effect estimates and error-controlled significance. A machine learning model yields variable importance, a measure of how much a predictor contributes to the predictive performance of a particular algorithm on a particular dataset. Importance scores are not significance tests; they can be unstable across resampling and can be biased towards correlated predictors or predictors with many possible split points [74]; and a variable with high importance may tag the ancestry or trial structure rather than resistance biology. These features remain E1 when derived from a single dataset, as in the re-analysis by Ahn et al. [22] of the Osorio-Guarín panel [21], and the same logic applies to the pathogen-side network or ML prioritisation [39], which remains E3a and hypothesis-generating until perturbation demonstrates a phenotype.

For future FPR studies, machine learning should consequently be treated as a complementary analytical framework rather than as an evidentiary shortcut from genomic association to causality. As a minimum, claims of predictive or biological relevance should distinguish clearly among association significance, predictive importance and mechanistic evidence. Predictive models should be evaluated with resampling procedures that prevent information leakage between training and testing sets, and whenever the objective is breeding transferability rather than description of the discovery population, performance should be tested in genetically independent material. Population structure, relatedness and environmental heterogeneity should also be addressed explicitly because a model may obtain an apparently strong predictive performance by learning the ancestry or trial structure rather than disease-resistance biology.

External validation is particularly important in cacao. Random cross-validation within a highly related breeding population can yield substantially more optimistic predictive estimates than validation in an unrelated germplasm, a different breeding population or another environment [19]. The contrast between the relatively encouraging within-population predictive ability reported by Romero Navarro et al. [17] and the much lower cross-population performance observed by McElroy et al. [23] provides direct FPR-specific evidence for this problem. Future studies should therefore report clearly whether validation is random within the population, family-aware, population-independent, environment-independent or both genetically and environmentally independent.

Machine learning is likely to be most valuable when used for prioritisation across convergent evidence layers rather than as a replacement for those layers. A genomic feature that receives high predictive importance, overlaps a reproducible QTL or association interval, shows infection-responsive expression and subsequently produces a phenotype after perturbation would constitute a progressively stronger candidate than a feature supported by model importance alone. Conversely, increasingly sophisticated algorithms applied repeatedly to the same small genotype–phenotype dataset cannot by themselves move a claim beyond E1.

### 6.3. Multi-Omics: From an Aspirational List to a Testable Experimental Design

Multi-omics can contribute to FPR research only if the different molecular layers are generated from a biologically coherent experimental design. Simply comparing gene lists from one study with proteins or metabolites measured in unrelated germplasms, tissues, pathogen isolates or infection stages does not constitute meaningful multi-omic integration. For the cacao–*M. roreri* interaction, the central requirement is therefore experimental pairing: the same host genotypes, physiological fruit stages, characterised pathogen material and infection timepoints should be represented across molecular layers, with the different assays derived, whenever technically possible, from aliquots of the same biological sample.

A practical FPR-specific discovery design should begin with cacao genotypes that provide reproducible contrasts in the existing molecular literature. CATIE-R4 and CATIE-R7 can represent partial-resistant/tolerant backgrounds, whereas Pound 7 and CATIE-1000 provide susceptible comparators; UF-273 can be incorporated as an additional partial-resistant/tolerant reference because it is also prominent in the genetic literature [36,37]. These genotypes should not be interpreted as a universal differential set, but they provide a rational starting panel because direct host- and pathogen-side FPR transcriptomic evidence already exists in overlapping contrasting germplasms [36,37]. Subsequent validation should extend any molecular signature to additional, genetically diverse cacao backgrounds rather than assuming that a mechanism detected in these reference clones is species-wide.

Pathogen sampling requires equivalent discipline. Section 3.2 shows that the molecular lineage is not a reliable surrogate for aggressiveness and that host responses established against one isolate cannot automatically be generalised [8]. A discovery experiment that aims to generalise beyond one host–isolate combination should therefore include more than one phenotypically characterised isolate, chosen for documented differences in aggressiveness rather than genetic divergence alone. Where the cost of full multi-omic profiling makes a factorial multi-isolate experiment impractical, a staged design is a reasonable alternative: one fully characterised isolate for discovery, followed by targeted validation of the resulting signatures with additional isolates. The number of isolates is a design choice determined by the scope of the intended claim, not a validated minimum.

Temporal resolution is equally critical because symptomless colonisation, the transition to necrotrophy, tissue destruction and sporulation are biologically distinct [32,38,49]. For illustration only, a design might include a mock-inoculated baseline, an early post-penetration point, several points spanning the symptomless phase and a sample at the recorded onset of necrosis and sporulation. Specific days after inoculation are not proposed as validated biological boundaries, because the duration of the biotrophic phase varies with host genotype, pod age and environment [38,55]. Sampling should therefore be anchored to the observed infection stage as well as chronological time: each fruit should be assigned days to the first external symptom and time to visible sporulation, so that the chronological time and biological stage can be analysed separately, and pilot experiments with the chosen genotypes and isolates should be used to place the sampling points.

Sampling should be destructive, with independent fruits harvested at each timepoint rather than repeatedly removing tissue from the same pod. A standardised mesocarp region centred on, and immediately adjacent to, the inoculation site should be collected from each harvested fruit, homogenised under conditions compatible with the intended assays and divided into matched aliquots. This approach allows transcriptomic, proteomic and metabolomic measurements to refer to the same underlying host–pathogen interaction rather than to different fruits that happen to share a treatment label. Parallel tissue should be retained for histological assessment and pathogen-biomass quantification, thereby allowing molecular changes to be interpreted relative to the amount and developmental state of the fungus present in the tissue.

As a minimum, the molecular core should combine pangenome-aware host genotyping, dual host–pathogen RNA sequencing, quantitative proteomics and metabolomics. Host genomic information provides the stable genetic background against which inducible molecular responses can be interpreted, whereas dual RNA sequencing captures simultaneous transcriptional reprogramming of cacao and *M. roreri*. The direct transcriptomic studies already available demonstrate that both genomes respond strongly during successful infection [36,37], while genome/secretome and subsequent pathogen studies identify secreted proteins, candidate effectors and regulatory programmes that can be interrogated more directly in such an integrated experiment [34,35,38,39]. Proteomics should determine whether transcript-level changes are accompanied by corresponding changes in host defence proteins and pathogen-derived proteins, while metabolomics should quantify the biochemical consequences of those responses. Targeted measurement of defence-associated hormones and phenolic compounds can complement untargeted metabolite profiling because these pathways are already implicated directly in the FPR host response [36].

Experimental controls are not secondary to the omic technologies. For every genotype and time point, mock-inoculated fruits of the same physiological age should be maintained under the same conditions as inoculated fruits; fruits should be distributed across multiple trees, treatment allocation randomised or blocked, and tree identity retained in the statistical model. The number of independent biological replicates (fruits from different trees) per genotype × isolate × time combination should be determined prospectively by power analysis or simulation from pilot estimates of biological variance and the effect sizes of interest [75], rather than fixed in advance; the earlier suggestion of four replicates should be read only as an illustrative starting point for a pilot. Technical replicates cannot substitute for independent fruits or trees.

The critical analytical endpoint is not the number of differentially expressed genes, proteins or metabolites recovered from such an experiment, but convergence among molecular layers and disease phenotypes. Candidate mechanisms should therefore be prioritised when host genetic variation, host or pathogen transcription, protein abundance and metabolite profiles converge on the same biological process and when variation in that process is associated with defined FPR components such as infection incidence, incubation period, internal severity, pathogen biomass or time to sporulation. Integration should be conducted at the sample level using matched biological identifiers, rather than by post hoc comparison of independently generated lists.

Even such convergence would not by itself establish causality. A multi-omic signature observed directly during *M. roreri* infection would provide strong E3a molecular support and could substantially improve candidate prioritisation, but concordance among transcriptomic, proteomic and metabolomic layers does not constitute functional perturbation. Progression to E3b would still require experimental perturbation of a prioritised host or pathogen determinant, and E4 would require stable genetic manipulation followed by a change in FPR in a pod-challenge assay (Table 1). Multi-omics should therefore be positioned as a mechanism-discovery and evidence-convergence strategy that narrows the transition from association to causality, rather than as a substitute for causal experimentation.

### 6.4. Epigenetics and Regulatory Plasticity: Plausible Mechanisms, Not Established FPR Determinants

Epigenetic regulation represents a plausible additional layer through which cacao and *M. roreri* could modify their interaction without changes in underlying DNA sequence. DNA methylation, histone modifications, chromatin remodelling and small regulatory RNAs can influence the amplitude, timing and persistence of plant immune responses in other plant pathosystems [76]. However, evidence from those systems should not be transferred automatically to FPR. Within the claim-specific classification used here, general evidence that epigenetic mechanisms regulate plant immunity remains E0 when invoked specifically to explain resistance to *M. roreri* unless the corresponding mechanism is demonstrated directly in the cacao–FPR interaction.

Direct host transcriptomic studies show that cacao genotypes differing in FPR response activate different defence-associated programmes [36], and such differences could in principle be influenced by the chromatin state, DNA methylation or small RNAs. None of the FPR-specific studies retrieved, however, established that such mechanisms explain the contrasting responses. On the pathogen side, a methyltransferase-related candidate prioritised from infection-associated transcriptomes [39] is appropriately retained as E3a, hypothesis-generating evidence; its annotation does not demonstrate that it modifies DNA, histones or another substrate during infection, nor that such modification alters virulence. Epigenetic memory or transgenerational priming has not been demonstrated for FPR and should not currently be presented as a breeding mechanism.

A rigorous test would pair transcriptomic responses with methylome, chromatin-accessibility and small-RNA measurements from the same material within the design of Section 6.3, include mock-inoculated controls to separate genotype-specific baseline differences from infection-induced changes, and sample several infection stages. An infection-associated methylation change coupled with altered transcript abundance would constitute E3a; experimental alteration of the regulatory state followed by a disease-related phenotype could provide E3b; and stable manipulation demonstrating a reproducible change in FPR would be required for E4. Until then, epigenetics belongs to the discovery-oriented part of the research agenda.

## 7. From Prediction to Breeding: MAS, Genomic Selection and Multi-Trait Design

Discovering genetic variation associated with FPR and using that information to generate genetic gain are different problems. QTL, GWAS signals, prediction models and molecular candidates differ in evidentiary maturity, and none improves a breeding programme merely because it can be measured. Translation requires matching the selection strategy to the genetic architecture of the trait, to the intended breeding product and to the strength and transferability of the underlying evidence. For FPR, the evidence supports a predominantly quantitative and partial architecture (Section 4), which favours approaches that accumulate effects distributed across the genome. Resistance is also not the sole objective: productivity, adaptation and bean quality determine whether a selected genotype has agronomic and commercial value, so the breeding problem is intrinsically multi-trait.

### 7.1. Phenotypic Selection Remains the Biological Anchor

Conventional improvement for reduced FPR has relied on identifying contrasting parents, generating segregating progenies, evaluating the disease response and agronomic performance, and recurrently retaining superior individuals or clones. This remains indispensable because the phenotype defines the breeding objective, and because it has demonstrably worked: the clone-evaluation and progeny trials summarised in Table 3 document selectable variation, and the CATIE-derived clones that now serve as “tolerant” references in the molecular literature were obtained by phenotypic selection [8,17,26,27,36]. Phenotypic selection is therefore the operational baseline against which molecular tools must demonstrate added value, not a technology they are intended to replace.

Several features of cacao make this process demanding: a multi-year juvenile phase, a complex late-acting self-incompatibility system [6], and a quantitatively inherited, environmentally dependent disease response whose heritability varies with population, scale and design (Section 3.5 and Section 4.2). Selection based on a terminal external-severity score may favour a different biology from selection on internal damage, delayed progression or yield maintained under disease pressure, which is why PRG-FPR (Section 3.4) is a precondition for both conventional and genomic selection rather than an auxiliary recommendation. The distinction between selecting parents, for which transmissible additive merit matters, and selecting clones for direct deployment, for which the entire realised genotype matters, becomes central in Section 7.4.

### 7.2. Marker-Assisted Selection: Useful Only When the Marker–Phenotype Relationship Is Transferable

Marker-assisted selection (MAS) is most efficient when a small number of loci explain a large, reproducible proportion of phenotypic variation and marker–trait associations remain stable across the populations in which they are applied. The FPR evidence does not currently satisfy these conditions. We therefore use three terms consistently. **Marker discovery** is the detection of a marker–trait association in one population (E1). **Marker validation** is the demonstration that the same association, with the same direction of effect, holds in material not used for discovery; validation in dependent material is E2a, and validation in genetically independent material is E2b. **Marker-assisted selection** is the operational use of a validated marker to make selection decisions, whose benefit is a separate claim on the translational axis (E5).

The Brown–Gutiérrez series illustrates why these distinctions matter. Brown et al. [15] identified five FPR QTL on linkage groups 2, 7 and 8 in 256 F1 individuals; Gutiérrez et al. [16], analysing the same Pound 7 × UF273 pedigree with a denser SNP map and separating external from internal severity, reported eleven QTL, of which four re-detected earlier signals and seven were new. The re-detected loci reach E2a, the new ones remain E1, and none has reached E2b. Re-mapping within one pedigree can strengthen confidence that a signal is reproducible in that background and improve localisation, but allele frequency, linkage disequilibrium, recombination history, genetic background and epistatic context differ among cacao groups, so transferability is a separate experimental question. McElroy et al. [23] tested it directly and found that FPR-associated markers were not consistently reproduced even among related Ecuadorian populations.

Meta-QTL analysis offers one route to prioritising regions that recur across mapping experiments [77], but a meta-QTL is an analytical synthesis of mapping evidence; it does not demonstrate that a linked marker predicts FPR response in an independent breeding population. Cacao has an established marker-assisted breeding methodology [78], yet methodological availability, locus discovery and operational benefit are distinct, and no individual FPR marker identified in the literature retrieved and verified has reached E5. For a predominantly quantitative trait, whole-genome prediction is conceptually better positioned than classical MAS to capture the cumulative contribution of many small-effect loci, which motivates Section 7.4; MAS retains a targeted role in introgressing or retaining independently validated regions, in parent selection and in pedigree management.

### 7.3. Genomic Selection and Prediction: Within-Population Accuracy Is Not Transferability

Genomic prediction is attractive for FPR because it exploits genome-wide information rather than individually validated loci, which matches the quantitative architecture described in Section 4. The selection objective must be defined explicitly: for parents, the relevant prediction is the genomic estimated breeding value, targeting transmissible additive merit; for clonal deployment, total genotypic value, potentially including non-additive effects, is the more appropriate target [79,80]. That this distinction is material in cacao, and not only in theory, is supported by diallel evidence from another cacao pathosystem, in which both general and specific combining ability were significant for foliar resistance to *Phytophthora* species, indicating a contribution of additive and non-additive effects [7]; the corresponding quantitative-genetic analysis has not been carried out for FPR.

Two direct FPR studies are available, and the reviewers rightly caution against comparing their headline numbers as if they measured the same thing. They do not, and the differences are summarised in Table 7. Romero Navarro et al. [17] analysed 148 improved clones at a single Costa Rican site under natural infection, using the proportion of diseased pods derived from monthly counts over eight years, and fitted GBLUP with a VanRaden genomic relationship matrix [18] under five-fold cross-validation repeated 50 times; the reported statistic is predictive ability, the correlation between predicted and observed clone values, which reached 0.60 with 90 markers and 0.67 with all markers. McElroy et al. [23] analysed three related Ecuadorian populations (Las Tecas, n = 589; Malvinas, n = 385; Ganadería, n = 391), using annual monilia-pod counts expressed as a percentage of total pods and adjusted by a mixed model for tree age, year, block, replicate and tree; they trained GBLUP on one entire population without cross-validation and applied it to the other two, so their statistic is the correlation between predicted and adjusted phenotypes in populations not used for training, which averaged approximately 0.16, with individual training-population values of 0.620, 0.373 and 0.661 within the respective training sets.

These values therefore differ in population structure and relatedness, trait definition, training-population composition, marker set, validation design and the quantity being correlated. Random cross-validation among related improved clones asks whether a model can interpolate among relatives drawn from one population, and is known to yield optimistic estimates when relatedness between training and validation sets is high [19]; prediction into a population not used for training asks a different and more demanding question. The appropriate conclusion is not that one study contradicts the other, but that exploitable predictive signal exists within characterised cacao populations while transferability across populations is weak and must be demonstrated rather than assumed. Neither study used predictions prospectively to make selection decisions, so neither reaches E5. Reporting conventions follow: every predictive claim should state the statistic, the validation design, the relatedness between training and target material, and whether validation was random, structured, external or forward.

Three practical consequences follow. Training populations should represent the genetic diversity of the material in which predictions will be made, retain sufficient relatedness to the selection candidates to support prediction, and be renewed as recombination and selection erode the relationship with later cycles; allele frequencies and linkage phases change, so a prediction equation developed once does not remain valid indefinitely. Phenotype quality limits what any model can recover, so PRG-FPR applies to the construction and updating of training populations as well as to mapping studies. Finally, because FPR expression depends on environment and on a genetically structured pathogen population whose lineage does not predict aggressiveness (Section 3.2), training datasets should anticipate host genotype × pathogen × environment effects rather than treating resistance as a host-only trait.

### 7.4. Multi-Trait Selection: Resistance Alone Is Not the Breeding Objective

A genotype that develops less disease but has poor productivity, inadequate adaptation or an unacceptable bean quality provides little benefit, and the clone-evaluation literature confirms that the most productive clones are frequently not the least diseased [25,26]. We therefore propose that FPR genomic selection be formulated as a multi-trait problem combining, as a minimum, a biologically defined disease component, productivity under the relevant production conditions, and market-relevant quality attributes [56], with additional adaptation traits where they are explicit programme objectives.

Translating multi-trait predictions into a selection decision is a classical problem in quantitative genetics, and established selection-index theory should be used rather than reinvented. The Smith–Hazel index combines trait values using economic weights together with the genetic and phenotypic variance–covariance structure [81,82]; subsequent developments provide restricted and desired-gains indices for situations in which trade-offs must be constrained, and formulations using estimated breeding values or genomic predictions in place of phenotypes [83]. Independent culling levels, in which candidates must exceed a minimum for each essential trait before being ranked, are likewise an established alternative to index selection. The weighting of components in a composite disease-and-production index [25,26] is an implicit version of the same decision, which we recommend making explicit.

What MIGGA-Advanced proposes is therefore not a new index but a specific combination of established tools: a multi-trait index with programme-defined weights, applied after independent culling levels have removed candidates failing minimum requirements for essential traits, so that mathematically favourable compensation cannot conceal unacceptable susceptibility or quality loss. Genetic correlations among the disease response, productivity and quality should be estimated within the target population rather than assumed, and re-estimated as germplasm and environmental change. Because parents and clones are different products, the index used for parental selection should emphasise additive merit, whereas clonal ranking should reflect total genotypic value and multi-environment performance [79,80]; the same index should not serve both decisions unless explicitly designed to. The literature retrieved and verified does not document an operational cacao programme in which the FPR response, productivity and bean quality have been combined in a validated multi-trait index and used prospectively, so this remains a testable proposal rather than a validated technology.

### 7.5. Evidence, Prediction and Deployment Are Different Dimensions

Technologies for cacao improvement differ in biological purpose, evidentiary maturity and proximity to deployment, and these should not be collapsed into a single continuum in which more sophisticated methods are assumed to deliver more gain. The selection response depends on the selection accuracy, intensity, available genetic variance and generation interval [79]; molecular tools can improve one or more of these, but capability does not guarantee transferability or realised gain. Table 8 therefore compares approaches on four separately assessed dimensions rather than assigning a single readiness label, because the earlier version’s composite categories (High, Moderate, Low) conflated evidence maturity, biological plausibility, functional validation and deployment readiness into one qualitative ranking. The assessment basis for each dimension is stated in the table footnote; the classification of the evidence column follows Table 1 and applies to specific claims, not to technologies as intrinsic entities. E3b-i evidence for the activity of individual *M. roreri* effectors, for instance, does not mean that gene editing has reached E3b for FPR.

Table 8 is not a ranking from inferior to superior technologies. The approaches address different bottlenecks: phenotypic selection supplies the biological reference and has delivered deployable clones; MAS exploits specific regions once validated; GWAS and machine learning support discovery; genomic selection integrates genome-wide predictive information; multi-omics and functional assays resolve mechanisms; and gene editing offers targeted intervention once causal evidence exists. The practical implication is to use each approach for the question its current evidence can support.

## 8. Precision Intervention: Gene Editing After Causal Evidence

Genome editing could modify defined host loci without the repeated recombination required by introgression, which is attractive in a species with long generation intervals, high heterozygosity and complex reproductive biology. For FPR, however, the limitation is not whether cacao can be edited but whether sufficiently validated causal targets exist. No FPR-specific host or pathogen determinant identified in the literature retrieved and verified has reached stable causal validation, so editing is positioned here as a tool downstream of causal evidence rather than as a route from candidate lists to presumed resistance genes.

### 8.1. Association Is Not an Editing Target

Before a sequence is edited, it must be established that the sequence itself, rather than a linked marker, a neighbouring gene, a correlated expression pattern or a population-specific haplotype, contributes causally to the phenotype. Linkage disequilibrium can cause a significant marker to tag variation elsewhere in an interval containing many genes, so editing a conveniently annotated gene near an association signal tests a hypothesis generated by the mapping result rather than implementing a validated mechanism. The current FPR candidates illustrate the gap: the chromosome-1 signal did not survive FDR correction [21]; the six Bonferroni-significant sites of Romero Navarro et al. [17] are unreplicated; the machine learning rankings derive from the same dataset as the original GWAS [22]; and even the strongest loci reach only E2a within one pedigree [15,16]. Replication of an interval would establish reproducibility of a locus–phenotype relationship, not the identity of the causal gene; genetic replication and mechanistic resolution are complementary rather than interchangeable.

Direct molecular evidence narrows the candidate space without identifying an editing target: differential expression identifies genes whose activity changes during infection but does not distinguish necessity from redundancy, downstream consequence or correlation with a broader physiological state [36]. On the pathogen side, the activity of a recombinant effector is not evidence that deletion of the corresponding gene changes virulence [34,35]. An editing target should therefore be defined by the causal hypothesis being tested: for a host susceptibility hypothesis, that loss or alteration of host-gene function reduces FPR without unacceptable penalties; for a host regulator, that modified function reproducibly reduces disease; for a pathogen determinant, its manipulation alters a defined component of pathogenicity. Importantly, the classification system is claim-specific and does not require a candidate to pass through every preceding class: a candidate with strong convergent molecular and perturbational support can justify a causal experiment without prior population-level replication, provided the hypothesis is specific and falsifiable.

### 8.2. What Cacao CRISPR Has Demonstrated: The TcNPR3 Case

The TcNPR3 studies demonstrate that editing can serve both as a functional-genomics tool and as a potential route to resistance improvement in cacao, and they also show why pathosystem specificity matters. Fister et al. [42] delivered Cas9 and guide RNAs targeting TcNPR3, a negative regulator of defence, into cacao leaf and cotyledon tissue by transient *Agrobacterium*-mediated transformation; deletions were detected in about 27% of TcNPR3 copies, and challenge with *Phytophthora tropicalis* showed reduced susceptibility and increased expression of downstream defence genes, with no off-target mutations detected above the background. These were genetically mosaic tissues, so this represents perturbational rather than stable evidence.

Guiltinan et al. [41] subsequently grew two independently regenerated T0 edited lines to maturity and crossed them with non-transgenic cacao; whole-genome sequencing of the parents and 22 progeny identified single T-DNA insertions and demonstrated segregation of the npr3 mutations from the editing transgene, with 7 progeny retaining edited alleles without detectable T-DNA. Detached-leaf challenge with *Phytophthora palmivora* showed an approximately 42% smaller lesion area in edited plants, which were phenotypically comparable to the wild type under the conditions examined, and transcriptomic analysis showed altered basal expression of reactive-oxygen-species, defence-transcription-factor and pathogenesis-related genes. We treat this as E4 for stable causal host manipulation in the cacao–*Phytophthora* pathosystem, bounded by its experimental scope: disease response was assessed primarily in detached-leaf assays, and field performance and breeding utility were not demonstrated, so it does not reach E5. A published correction reassigned the line GM-249, originally identified as Scavina-6, to ICS-1 following whole-genome sequencing; the principal conclusions on allele inheritance and reduced *Phytophthora* susceptibility were not withdrawn or revised, and the reassignment indicates greater background divergence in the affected comparisons than originally reported [41].

For FPR, the interpretation is different, because neither study challenged cacao with *M. roreri*. Reduced susceptibility to *Phytophthora* species cannot establish that TcNPR3 disruption will reduce frosty pod rot, given the fundamental differences in taxonomy and infection biology. TcNPR3 therefore retains its strong evidence for the cacao–*Phytophthora* system and remains E0 for FPR; it should not be described as a validated FPR susceptibility or resistance gene. Its value for this framework is as a technological and causal-validation precedent demonstrating that stable editing, transgene segregation and recovery of phenotypically normal edited progeny are achievable in cacao.

### 8.3. An Illustrative Design for Causal Validation in the FPR Pathosystem

The mechanistic gap is now specific enough to define the kind of experiment that would close it. The following design is proposed by the authors as an illustration of what an E4-level result would require under the definition in Table 1; it is not a validated protocol, and the parameters are design choices to be set prospectively from pilot data rather than minimum requirements.

For a host candidate, the comparison is between independently generated stable lines carrying the intended alteration and matched non-edited controls in the same or a closely matched background. Independent transformation events should be compared, because a phenotype observed in a single regenerant cannot be distinguished from event-specific, somaclonal or unintended genomic effects; segregants lacking the edited allele provide an additional control for tissue-culture history. For a pathogen candidate, stable alteration of the *M. roreri* determinant is compared with the parental strain, and complementation or allelic rescue provides the strongest causal test by asking whether the altered phenotype is recovered when function is restored. For candidates such as MrB1, which currently have E3b-i activity evidence, this genetic bridge is precisely what is missing [35].

The disease assay should be designed around the biology of FPR rather than a single terminal score, following PRG-FPR Modules A and B. Because FPR is a pod disease, the biologically relevant assay is a challenge of pods of a defined developmental stage, attached where possible, or detached if detached-pod responses have been validated against attached pods; whole-plant phenotyping is not required. The incidence, external severity, internal severity, incubation period, time to sporulation and disease progression should be analysed separately when the causal hypothesis predicts effects on different stages, because a candidate that reduces external symptoms without altering internal colonisation supports a different mechanism from one that reduces infection establishment or sporulation. The number of pods, trees and independent experimental runs should be determined by prospective power analysis from pilot variance estimates.

Pathogen and host diversity determine the scope of the resulting claim, not its validity. A causal effect demonstrated against one isolate establishes an isolate-specific interaction; extending the claim requires additional phenotypically characterised isolates, chosen on aggressiveness rather than lineage [8]. Similarly, an effect in one cacao background does not demonstrate transferability across cacao diversity, which matters particularly for immune regulators and susceptibility genes whose developmental and quality consequences may be background-dependent. We therefore propose that the criteria for accepting a causal claim be specified before the experiment: confirmation of the intended genetic change; reproduction of the directional phenotype across independent events or strains; and a change in a defined FPR component in a direct pod-challenge assay under documented phenotyping. Results confined to a single event, lost in an independent line, dependent on an inadequately characterised inoculum, or inseparable from regeneration, background or fitness effects do not meet them, and a candidate that alters a molecular marker without changing any disease component remains below E4.

### 8.4. Target Choice, Pleiotropy and Durability

Causal efficacy and breeding suitability are separate claims. An E4 result shows that manipulating a determinant alters FPR under the conditions tested; deployment requires that the intervention not compromise the functions, agronomic performance or product quality that determine the value of a cacao genotype. This matters particularly for immune regulators and susceptibility genes, which may influence several processes simultaneously: a candidate producing a strong FPR phenotype but reducing growth, reproductive development, pod production, adaptation or bean quality would remain mechanistically informative yet unsuitable for deployment. The TcNPR3 case illustrates the boundary without providing FPR evidence: edited material was reported as phenotypically comparable to the wild type under the conditions examined [41], but that study was not designed to establish field performance, reproductive stability, yield, quality or multi-environment adaptation, so phenotypic normality under controlled conditions and breeding suitability remain distinct claims.

Where complete loss of function produces unacceptable pleiotropic effects, the causal result still informs biology but indicates that full knockout is inappropriate; naturally occurring alleles or regulatory variants that alter the disease-relevant function while retaining essential activity may be better hypotheses, since the aim of precision breeding is to reproduce a favourable biological state rather than to maximise molecular disruption. Durability is likewise a property requiring evidence rather than an attribute of a target: a modification effective against one isolate may be narrow with respect to pathogen diversity, whereas a directional response conserved across phenotypically distinct isolates provides stronger evidence of a broadly relevant mechanism. The current literature does not support ranking candidate genes by expected durability, and such a ranking would be premature. Accordingly, we recommend avoiding “durable”, “broad-spectrum” or “resistance-breaking proof” for any locus or intervention, in favour of formulations bounded by what was tested, such as a response conserved across the evaluated isolates or reduced susceptibility within the tested deployment domain.

### 8.5. Editing the Host Versus Editing the Pathogen

Host and pathogen manipulation answer different questions despite using similar technologies. Editing a cacao gene asks whether a heritable host modification alters FPR and could eventually enter a cultivar or parent; manipulating a pathogen determinant asks whether that determinant contributes causally to infection, colonisation, tissue damage or sporulation. A pathogen-side causal result would be E4 for the biological role of that determinant, but a manipulated pathogen is not a breeding product: its translational value lies in sharpening host-side hypotheses, identifying processes the plant must restrict, and prioritising host pathways that interact with validated pathogen functions. Only host manipulation has a direct route towards breeding use, and only after pleiotropy, background dependence, durability and agronomic performance have been evaluated.

This two-genome logic prevents a common category error: a pathogen gene required for virulence is not thereby a host resistance gene, and a cacao transcript induced during infection is not thereby a susceptibility determinant. The current asymmetry, with pathogen-side evidence at E3b-i and host-side evidence at E3a, reflects which type of claim has advanced further, not which side is closer to breeding. For a hemibiotrophic pathogen whose biology changes markedly between symptomless colonisation, the transition to necrotrophy, tissue destruction and sporulation [37,38], integrating causal evidence from both genomes helps determine which stage a candidate affects rather than collapsing these processes into a generic claim of resistance. Section 8 therefore reaches a deliberately conservative conclusion: editing is technically feasible in cacao, and candidates exist on both sides of the interaction, but what limits precision intervention for FPR is the maturity of the causal evidence, not the availability of the technology.

## 9. MIGGA: A Proposed Modular, Evidence-Gated Framework

The evidence synthesised above does not support a single pipeline in which increasingly sophisticated methods must be adopted sequentially before genetic gain against FPR can occur. Phenotypic selection, genomic prediction, multi-trait selection, pangenomic information, multi-omics, functional genetics and genome editing address different questions and occupy markedly different positions with respect to FPR-specific evidence. MIGGA (Marco Integrador de Ganancia Genética Acelerada) is therefore proposed as a modular, evidence-gated framework rather than a technological sequence.

Its status should be stated plainly at the outset. MIGGA is an author-derived proposal. The literature reviewed provides evidence for exploitable genetic variation, marker associations, candidate genes and a genomic prediction signal; it does not demonstrate that implementing MIGGA increases realised genetic gain, shortens breeding cycles, improves selection decisions or offers economic advantage relative to existing breeding strategies, and no such evaluation has been attempted. MIGGA is thus a framework designed to facilitate genetic gain, not a framework whose effectiveness has been demonstrated. Its components are drawn from established practice, including conventional selection, selection-index theory [81,82], independent culling levels, genomic selection methodology [70,80,84] and standard functional-genetics logic; what MIGGA contributes is their organisation around explicitly stated evidentiary criteria for FPR. Its value is therefore testable: Section 11 identifies the comparisons that would evaluate it, and Section 9.3 shows what its application looks like retrospectively, which is the strongest illustration the current literature permits.

### 9.1. Three Tiers, Operating in Parallel

MIGGA-Core is the operational breeding system: a defined target population and germplasm base, PRG-FPR-aligned phenotyping, genome-wide genotyping linked to a defined breeding question, selection, prospective validation of selected material and multiplication, with diversity tracked alongside gain (Section 10.2). Core does not depend on identifying causal genes or implementing multi-omics, and its irreducible component is the phenotype–selection loop; genomic prediction is a decision accelerator within Core, not a prerequisite for legitimate progress. A programme with high-quality phenotypes but insufficient prediction accuracy can select phenotypically, whereas a programme with extensive genotyping but unreliable phenotypes has not established a functioning Core.

MIGGA-Advanced adds multi-trait prediction and selection indices with independent culling levels (Section 7.4) and, where justified, higher-throughput phenotyping or pangenome-aware genomic information. Each component should be adopted only when it addresses a recognised Core limitation and its incremental value can be measured against the simpler alternative, because higher-resolution measurement increases the cost without necessarily changing a breeding decision. Digital phenotypes should be calibrated against biologically interpretable traits before entering selection, since a measure of external lesion development cannot substitute for internal severity or productivity under disease pressure if those are the traits of interest. Pangenomic resources [72,73] expand the searchable variation space through presence/absence, copy-number and structural variants, but the cacao pangenome did not demonstrate that any variant class controls FPR [72], so pangenomics enlarges the search space without raising the class of an FPR claim.

MIGGA-Frontier addresses which host or pathogen determinants influence the interaction and whether manipulating them changes FPR: evidence convergence and candidate reduction, functional testing, stable causal validation and, where justified, precision intervention (Section 6.3 and Section 8.3). Frontier is not characterised by a single class, because the classification is claim-specific: host molecular evidence is at E3a, and pathogen effector activity at E3b-i, with no FPR-specific E4 or E5 identified. Nor is Frontier a prerequisite for Core or Advanced progress; mechanistic research proceeds in parallel while populations continue to be phenotyped, genotyped and selected. The tiers are reciprocal: Core supplies the phenotypes, germplasm and populations the other tiers require; Advanced improves how Core candidates are ranked; and Frontier returns validated mechanisms or refined hypotheses, which may sharpen phenotyping priorities even when no molecular candidate is ever deployed.

### 9.2. Decision Criteria, and How They Would Be Specified Prospectively

The modular structure requires rules for deciding when evidence is sufficient for a particular next use. We propose five decision points covering phenotype qualification, predictive use, causal validation, breeding acceptability and prospective breeding utility. These are author-proposed decision rules, not established standards, and the labels GO and NO-GO denote the two possible outcomes of a pre-specified comparison, not an authoritative judgement.

The reviewers correctly observed that, in the earlier version, terms such as “meaningful advantage”, “acceptable”, “sufficiently reliable” and “documented improvement” were not operationalised, leaving the gates conceptual. A universal numerical threshold is neither possible nor desirable, because appropriate thresholds depend on the breeding objective, population size, cycle structure, cost and realistic alternative. What can be specified is the structure of the decision. We therefore propose that each gate be defined prospectively, before the result is known, by six elements: the claim being tested; the comparator, which must be the programme’s realistic current practice rather than a null alternative; the primary endpoint, stated as a single quantity; the decision rule, including the programme-specific threshold and the direction of the comparison; the treatment of uncertainty, including how the endpoint’s standard error or interval is estimated and what degree of uncertainty is tolerable; and the replication or validation structure over which the endpoint is estimated. Table 9 gives these elements for each gate, with the quantities a programme must fix shown as programme-defined parameters. A NO-GO restricts the scope of a claim and identifies where to return; it does not invalidate the preceding evidence, since a model may be statistically informative but insufficiently transferable, and a causal mechanism may be valid but agronomically unsuitable.

### 9.3. A Retrospective Worked Example

Because no programme has yet implemented MIGGA, its added value cannot be demonstrated here. What can be shown is how the framework classifies and routes evidence that already exists. The following worked example applies the decision structure of Table 9 retrospectively to the best-documented FPR resources: the Pound 7 × UF273 QTL series [15,16] that the CATIE clonal panel used for genomic prediction [17], and the three Ecuadorian populations used for cross-population prediction [23]. It is an illustration of the framework’s logic, not evidence that applying it would have improved these programmes.

**Phenotype qualification.** The Gutiérrez et al. protocol [16] is the most explicitly standardised FPR phenotype among the mapping studies: attached pods of defined developmental stage, repeated artificial inoculation, bagging to maintain humidity, and external and internal severity scored separately on defined 0–5 scales at a fixed interval after challenge, with minimum numbers of fruits and inoculation events per genotype. Against PRG-FPR Module A, this qualifies for QTL mapping, with the limitation that inoculum concentration, viability and isolate identity are not reported at the level Module B recommends, so the phenotype cannot be reproduced exactly elsewhere. The field phenotypes of [17,23] qualify for genomic prediction within their own programmes under Module C, with the caveat that incidence is a ratio whose denominator is a productivity trait (Section 3.5). The decision this supports is therefore to proceed with mapping and prediction within each programme, while recording the inoculum and denominator information that would make the phenotypes comparable across programmes.

**Genetic claims.** Four loci re-detected across [15,16] are classified as E2a. Under the structure of Table 9, the question for a breeding programme is whether to use them in MAS, which requires replication in material that is genetically independent of the Pound 7 × UF273 pedigree. The chromosome-5 QTL from the separate Type 2 family in [17] does not provide that replication, both because the family shares the Pound 7 parent and because its 68 individuals mean the 28.8% of variance explained is likely to be overestimated [20]. The relevant decision is therefore NO-GO for routine marker-assisted deployment and a specific, feasible next experiment: pre-specified testing of these four intervals in an unrelated population phenotyped for both external and internal severity.

**Predictive claims.** Within the CATIE panel, the predictive ability of 0.60–0.67 under random five-fold cross-validation [17] supports use within that population and that trait definition. When applying the structure of Table 9, the comparator would be the programme’s existing phenotypic selection, and the endpoint would be predictive ability in material not used to fit the model; the McElroy results [23] show what happens when this is attempted across populations, with a mean of approximately 0.16 in populations not used for training. The routing is therefore GO for within-population use with periodic recalibration, NO-GO for transfer to genetically distinct material without retraining, and a specific next step: structured cross-family or cross-cycle validation within the CATIE programme, followed by prospective prediction of unphenotyped candidates, which is the step that would generate the first E5 evidence for FPR.

**Mechanistic claims.** MrB1 has E3b-i evidence: the recombinant protein causes genotype-dependent necrosis in cacao leaves and localised necrosis in fruits [35]. According to Table 9, this is not a breeding decision at all; it routes to the causal-validation point, where the relevant experiment is stable disruption and complementation in *M. roreri* followed by pod challenge (Section 8.3). The example also shows the framework rejecting an apparently attractive shortcut: the genotype-dependent necrosis observed across cacao clones might suggest using MrB1 sensitivity as a screening assay, but until a contribution to disease is demonstrated, the correlation between protein sensitivity and field resistance is untested, and the assay would qualify only as a research tool.

Three features of this example are worth noting. First, the framework separates decisions that the literature currently conflates: the same body of work yields GO for within-population prediction, NO-GO for marker deployment and a mechanistic route with no breeding decision attached. Second, every NO-GO identifies a specific, feasible experiment rather than terminating a line of work. Third, the exercise is retrospective and therefore cannot show that these routings would have improved the programmes concerned; establishing that would require the prospective comparisons described in Section 11.

### 9.4. Reading the Framework

Two axes should not be confused. The tiers describe the type of activity underway; the decision points assess whether evidence supporting a given claim is adequate for its proposed next use. A tier is not an evidence class, and a decision point is not a chronological stage the whole programme must pass. The tiers operate simultaneously: the absence of a causal gene does not prevent phenotypic or genomic selection, and an advanced genomic platform does not justify advancing an insufficiently supported molecular candidate. Progress is also iterative, since phenotype qualification is revisited as environments change, prediction models are recalibrated as allele frequencies change, and acceptability is reassessed as objectives change; a previous GO licenses a defined use under the conditions tested rather than conferring a permanent status. Finally, a GO should expand the scope of permitted inference only as far as the evidence supports: a phenotype qualified in one experiment does not become universal, a model validated in one population does not become population-independent, and an acceptable experimental performance does not constitute E5 until prospective use produces a documented benefit.

Figure 2 represents this organisation. A programme achieving reproducible phenotyping and documented genetic gain through Core and Advanced has succeeded even without genome editing, whereas technically successful editing without reliable phenotyping or a prospective breeding benefit has not. The operational implications of this synthesis and of the MIGGA framework for cacao breeding programmes are summarised in Box 1.

Box 1 **Operational take-home messages for cacao breeding programmes.**

**Start with the phenotype.** Use the proposed PRG-FPR (Table 2) as a reporting and design checklist, recognising that it has not been validated by inter-laboratory testing. More markers or more complex models cannot compensate for an ill-defined FPR phenotype. **Prioritise validation over additional marker discovery.** The strongest FPR QTL evidence reaches E2a. Replicate promising locus–phenotype relationships in genetically independent cacao material before considering routine marker-assisted deployment. **Genomic prediction is population-dependent.** Train, recalibrate and validate models in material representative of the intended selection domain, and report the statistic and validation design; within-population predictive ability and cross-population transferability are not comparable quantities. **FPR-specific causal targets are not yet available.** Host molecular evidence reaches E3a and pathogen effector activity reaches E3b-i, but no FPR-specific determinant identified here has reached E4. Precision intervention should test causal hypotheses before being considered a breeding route. **Breed for the whole genotype.** Combine a reduced disease impact with an acceptable productivity, adaptation and bean quality, using an established selection-index and independent-culling methodology. **Judge tools by realised breeding benefit.** Marker significance, prediction accuracy and causal validation do not constitute E5, which requires prospective use in a breeding decision and documented improvement against a pre-specified comparator.

## 10. Translational Context: Deployment, Diversity, Regulation and Adoption

The considerations in this section condition whether genetic gain becomes usable in production. They are contextual rather than part of the evidence synthesis, and they are summarised briefly here; satisfying MIGGA’s evidentiary criteria confers no legal, regulatory or commercial permission to deploy, and regulatory or commercial clearance does not raise the class of the underlying scientific evidence.

### 10.1. Durability and Deployment

Durability is an empirical property of a deployment strategy, not a label attached to a gene, QTL or genotype, and it can only be established prospectively. Efficacy demonstrated in one host background, against one isolate or in one environment says nothing about how long an advantage will persist under widespread cultivation. For *M. roreri*, geographically structured diversity [50,51] and isolate differences in aggressiveness that are not predicted by lineage [8] mean that deployment decisions should be informed by phenotypically characterised pathogen material rather than lineage labels. Because cacao is clonally propagated, a genotype can be reproduced widely without recombination, so clonal fidelity and landscape-level uniformity should be managed separately: deployment portfolios retaining more than one acceptable resistance source avoid coupling regional production to a single host configuration, without any claim that mixtures have been shown to prevent adaptation of the pathogen. Post-deployment monitoring of FPR performance, productivity and quality should return to the breeding programme, and apparent loss of resistance should prompt characterisation of the pathogen before being attributed to resistance breakdown rather than to the environment or management.

### 10.2. Genetic Diversity as a Constraint and an Objective

Diversity is both the source of genetic gain and a constraint on how aggressively it can be exploited. Cacao diversity is geographically structured, with substantial variation in the Amazonian, wild, semi-domesticated and ex situ germplasm that is incompletely represented in breeding populations [2,71]; membership of a diverse group is not itself evidence of FPR resistance, so the germplasm should enter breeding on a measured phenotype or genetic contribution. Repeatedly concentrating contributions from a few superior parents improves the current cohort while narrowing the base for later gains, so parental selection should consider genomic or pedigree relationships and family contributions alongside FPR performance. We do not prescribe an effective-population-size threshold, a maximum coancestry or a compulsory optimal-contribution algorithm, since the appropriate balance depends on the programme size, mating design and germplasm; relationship-aware methods such as optimal contribution selection are well established [85] and should be justified by the programme rather than presented as an FPR-specific requirement. Terminology follows standard quantitative-genetic usage: coancestry (kinship) is the probability that alleles drawn at random from two individuals are identical by descent; relatedness is twice the coancestry for non-inbred individuals; and inbreeding of an individual equals the coancestry of its parents. Breeding-population diversity and deployment diversity are distinct, because extensive planting of a few clones can produce uniformity at the production scale even when the breeding population remains diverse.

### 10.3. Regulatory Classification

Regulatory pathways for a future FPR-oriented precision intervention cannot be inferred from the editing technology alone; across the principal Latin American cacao-producing countries, classification depends substantially on the characteristics of the resulting organism and on whether a new or foreign genetic combination remains in the final product. Because these frameworks change, the date of verification is reported explicitly and should be re-checked before any field release: the national instruments summarised below were verified against official primary sources on 30 August 2026, when the original search and verification process was completed, and this date applies to all statements in this subsection.

In Colombia, ICA Resolución No. 00022991 of 2022 establishes the procedure for determining whether a product obtained through innovation in genetic improvement corresponds to an Organismo Vivo Modificado (OVM) or to a conventional organism, applying where the final product does not contain foreign genetic material [86]. In Ecuador, Acuerdo Ministerial No. 063, published in Registro Oficial No. 479 on 17 January 2024, provides a technical guide for seeds or crops obtained by precision-breeding techniques and establishes a preliminary assessment considering whether the material contains a new combination of genetic material and whether material used transiently remains in the final organism [87]. In Peru, Resolución Ministerial No. D000068-2026-MINAM-DM of 6 March 2026 establishes guidelines for determining the OVM status for products developed using new biotechnological tools, within the framework of Law No. 29811 as extended by Law No. 31111 [88]. In Brazil, Resolução Normativa CTNBio No. 16 of 15 January 2018 establishes the consultation procedure for Técnicas Inovadoras de Melhoramento de Precisão, allowing products to be considered non-GMO when the applicable criteria are met, including the absence of recombinant DNA/RNA from the resulting product, with case-by-case technical evaluation by CTNBio [89]. In Costa Rica, Decreto Ejecutivo No. 44244-MAG of 6 September 2023, effective 10 November 2023, amended the Reglamento a la Ley de Protección Fitosanitaria to incorporate new genetic improvement techniques and establishes a case-by-case procedure before the Unidad de Organismos Genéticamente Modificados; where the authority can determine that no new combination of genetic material has been generated or remains, the organism is not subject to the framework applicable to agricultural OVMs [90].

These instruments do not support a binary statement that gene-edited crops are either regulated or exempt; they illustrate a case-specific logic in which the molecular characteristics of the resulting organism are central, and a determination in one jurisdiction does not transfer automatically to another. Nor does a determination that a product is not regulated as an OVM remove obligations relating to varietal registration, phytosanitary requirements, movement of propagative material, intellectual property or access and benefit sharing.

### 10.4. Germplasm Provenance and Benefit Sharing

Cacao improvement depends on the germplasm distributed among breeding populations, ex situ collections and wild or semi-domesticated populations across the Neotropics [2,71], which makes provenance and benefit-sharing relevant to FPR breeding. The Evidence Atlas is not an accession-by-accession legal provenance audit, and no assumption is made that resistance-associated alleles are uniformly Amazonian in origin or subject to a single access regime. The defensible principle is traceable provenance: for a germplasm entering a breeding programme, the accession identity, source collection, country and institution of origin, acquisition history, transfer documentation and conditions on subsequent use should be recorded as far as records permit. The international framework is provided principally by the Convention on Biological Diversity and the Nagoya Protocol [91], under which access may be subject to prior informed consent and mutually agreed terms depending on the provider country’s legislation, and benefits may be monetary or non-monetary; for collaborative FPR research, non-monetary benefits may include reciprocal access to data, participation in research design, training, technology transfer and strengthening of partner infrastructure. Benefit-sharing discussions now extend beyond the physical germplasm to digital sequence information, for which COP16 adopted modalities including the Cali Fund [92], which is directly relevant because genomic selection, pangenomics and candidate prioritisation can proceed from sequence data without repeated physical transfer. Applicable obligations depend on the material, provenance, associated traditional knowledge and jurisdiction, and are therefore best assessed before access and utilisation rather than once a candidate acquires value.

### 10.5. Adoption and the Target Product Profile

Attributes that determine whether improved material can be used—productivity, adaptation, bean quality, propagation feasibility and market compatibility—are components of the breeding objective rather than considerations that begin after selection. Defining a target product profile at the outset makes trade-offs visible and testable: a clone for intensive clonal deployment faces different requirements from a recurrent-selection parent or a clone for diversified smallholder agroforestry, and because cultural FPR control requires recurrent labour [3,5], the relative value of genetic disease reduction differs among production systems. Multiplication capacity is a practical constraint, since a superior clone cannot generate an impact without genetically faithful, phytosanitarily sound planting material, and traceability through multiplication supports both breeding evaluation and post-deployment monitoring. Adoption should be distinguished from E5: E5 concerns whether using information improved a breeding decision, whereas farmer uptake and commercial acceptance are broader outcomes that may occur after, alongside or independently of that comparison. Adoption is not an additional evidence class or decision point.

## 11. A Research Agenda for FPR Genetics

### 11.1. Critical Evidence Gaps and Decisive Experiments

The gaps identified above do not have equal consequences for breeding progress. We define an experiment as decisive when both a confirming and a refuting result would change a decision: successful replication can move a locus from E2a to E2b, whereas a well-designed failed replication should lower its priority for marker-assisted use; stable manipulation that changes FPR can establish E4, whereas a well-controlled null result should narrow or reject the causal hypothesis; and prospective use of genomic information can establish E5 only if the resulting decision produces a documented benefit, while failure identifies a technology whose statistical promise has not translated into operational value. Table 10 summarises six such experiments addressing phenotype comparability, independent genetic replication, prediction transferability, stable causal validation, biological breadth and prospective breeding utility.

### 11.2. Provisional Ordering of Research Priorities

We also propose a provisional ordering of these experiments, and its basis should be stated explicitly. The ordering is author-derived and reflects our own judgement of four considerations: how many downstream decisions each gap conditions; whether the required germplasm, populations and infrastructure already exist; how directly a result could change breeding practice; and whether another gap must be addressed first. No formal weighting procedure, expert elicitation or consensus process was applied, and the criteria were not weighted numerically; a different programme weighting feasibility or mechanistic insight differently would reasonably reach a different order. The ordering is therefore provisional and revisable, and is offered to inform deliberation rather than to prescribe a sequence. These evidence gaps and their relationships across phenotyping, genetic replication, mechanistic validation and breeding utility are integrated conceptually in Figure 3. The diagram highlights where the current FPR evidence chain is interrupted and distinguishes transitions already supported by FPR-specific evidence from those that still require prospective validation.

On that basis, we would place phenotype comparability first, because phenotype definition is the most pervasive dependency in the evidence chain and a ring trial would simultaneously improve the foundation for QTL replication, prediction and causal validation. Prediction transferability follows, because genomic prediction is the approach closest to practice and its unresolved problem is portability rather than existence of signal. Prospective testing of genomic selection comes next, since no model has reached E5 and a prospective comparison would generate more translational information than further retrospective optimisation of accuracy; for a programme already operating within a well-connected population, this need not wait for universal cross-population transferability. Independent replication of the strongest loci follows, concentrating on a small number of clearly defined claims rather than accumulating further associations. Defining biological breadth through connected host × pathogen × environment validation becomes important as clones or interventions approach deployment decisions. Closing the gap from molecular evidence to stable causal validation is placed last, not because causal biology is unimportant, but because stable transformation, pathogen manipulation and complete pod-challenge validation are technically demanding and are not prerequisites for genetic gain through Core or Advanced breeding, which can proceed in parallel. Resource availability will legitimately alter this order locally: programmes with established phenotyped populations are best placed for prospective genomic-selection testing, while laboratories with transformation infrastructure can contribute to causal validation.

## 12. Conclusions

Frosty pod rot resistance in cacao is best understood as a quantitative, partial and context-dependent breeding trait rather than a phenotype governed by a single major determinant. The literature synthesised here demonstrates exploitable genetic variation and real opportunities for improvement, but the principal limitations to faster gain are no longer primarily technological: phenotype heterogeneity, incomplete transferability of genetic signals and prediction models, absence of stable FPR-specific causal validation and absence of prospective breeding-utility evidence now define the boundaries of the field.

At the genetic level, the strongest FPR QTL evidence has progressed beyond isolated discovery but remains short of independent replication: re-detection within the Pound 7 × UF273 pedigree supports E2a, and no locus replicated in genetically independent material was identified in the literature retrieved and verified. Genome-wide prediction offers one of the more credible near-term routes, but the two available direct studies answer different questions, and their reported values of 0.60–0.67 and approximately 0.16 differ in population structure, phenotype definition, validation design and the quantity correlated, so they should not be read as a single measure of performance. The molecular evidence has advanced, with host transcriptomics at E3a and recombinant pathogen effectors showing activity at E3b-i, including genotype-dependent necrosis in cacao tissues for MrB1; but no determinant has been stably manipulated and shown to change FPR in a pod-challenge assay, and the functional evidence for three further candidate effectors was obtained in tomato rather than cacao. Genome editing is technically feasible in cacao, as the TcNPR3 work shows for the *Phytophthora* pathosystem, but that evidence remains E0 for FPR.

A central contribution of this review is therefore to separate what can be measured, what can be predicted, what is causal and what is demonstrably useful for breeding. The classification system provides a claim-specific language for these distinctions without assigning artificial maturity levels to technologies, and its multidimensional structure makes explicit that genetic replication, mechanistic inference and breeding utility are different questions rather than steps on one ladder. The unresolved transitions are correspondingly clear: independent genetic replication, stable FPR-specific causal validation and prospective breeding utility.

The accompanying proposals should be read for what they are. PRG-FPR is a reporting and design guideline, not a validated standard; its elements are grounded in the biology of the infection cycle and in the methodological differences documented across the primary studies, but its value must be tested through coordinated multi-site work. MIGGA organises existing breeding, quantitative-genetic and functional-genetics methodology around explicitly stated evidentiary criteria for FPR; the literature reviewed supports the evidence classification on which it rests, but it does not demonstrate that implementing MIGGA increases realised genetic gain, shortens cycles or improves selection decisions relative to current practice. That remains an open question, and Section 11 identifies the comparisons that would answer it.

Progress against FPR does not need to wait for complete mechanistic resolution or genome editing. Reliable phenotyping, quantitative selection, appropriately validated genomic prediction and multi-trait breeding can generate progress now, as the clone-evaluation literature already demonstrates, while causal research advances in parallel. Conversely, increasingly sophisticated molecular technologies do not justify extrapolating beyond the evidence they currently support. The success of molecular breeding for FPR should ultimately be judged not by the number of markers discovered, omics layers generated or genes edited, but by whether those tools produce better, reproducible and prospectively validated breeding decisions that increase realised genetic gain while preserving productivity, adaptation, quality and future genetic capacity.

## Figures and Tables

**Figure 1:**
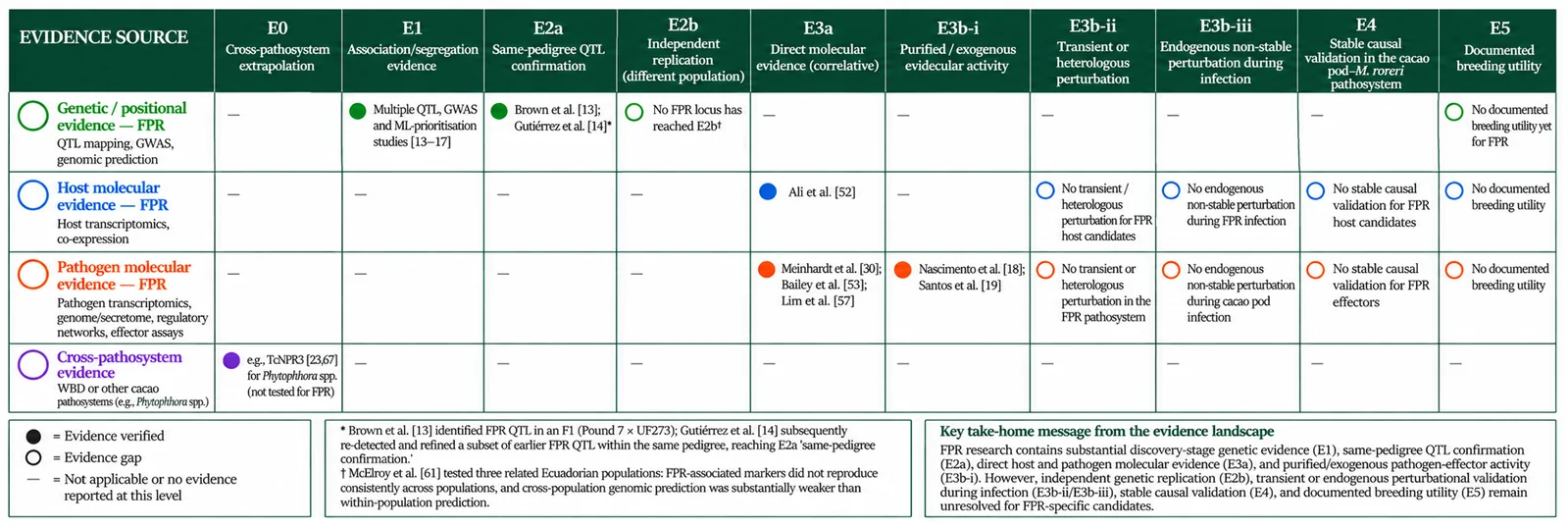
Evidence landscape for frosty pod rot (FPR) resistance in cacao, mapped onto the author-developed classification system of Table 1. The classification is the authors’ own and is not an established external evidence standard. Columns are grouped by axis (directness qualifier, genetic, mechanistic, translational); ordering is meaningful only within an axis. Filled circles indicate classes supported by verified evidence, open circles indicate unresolved evidence gaps, and dashes indicate classes that are not applicable or for which no evidence was identified at that level. Symbols are qualitative and do not represent publication counts, effect sizes or statistical strength. Numbers refer to the reference list. GWAS, genome-wide association study; QTL, quantitative trait locus; WBD, witches’ broom disease.

**Figure 2:**
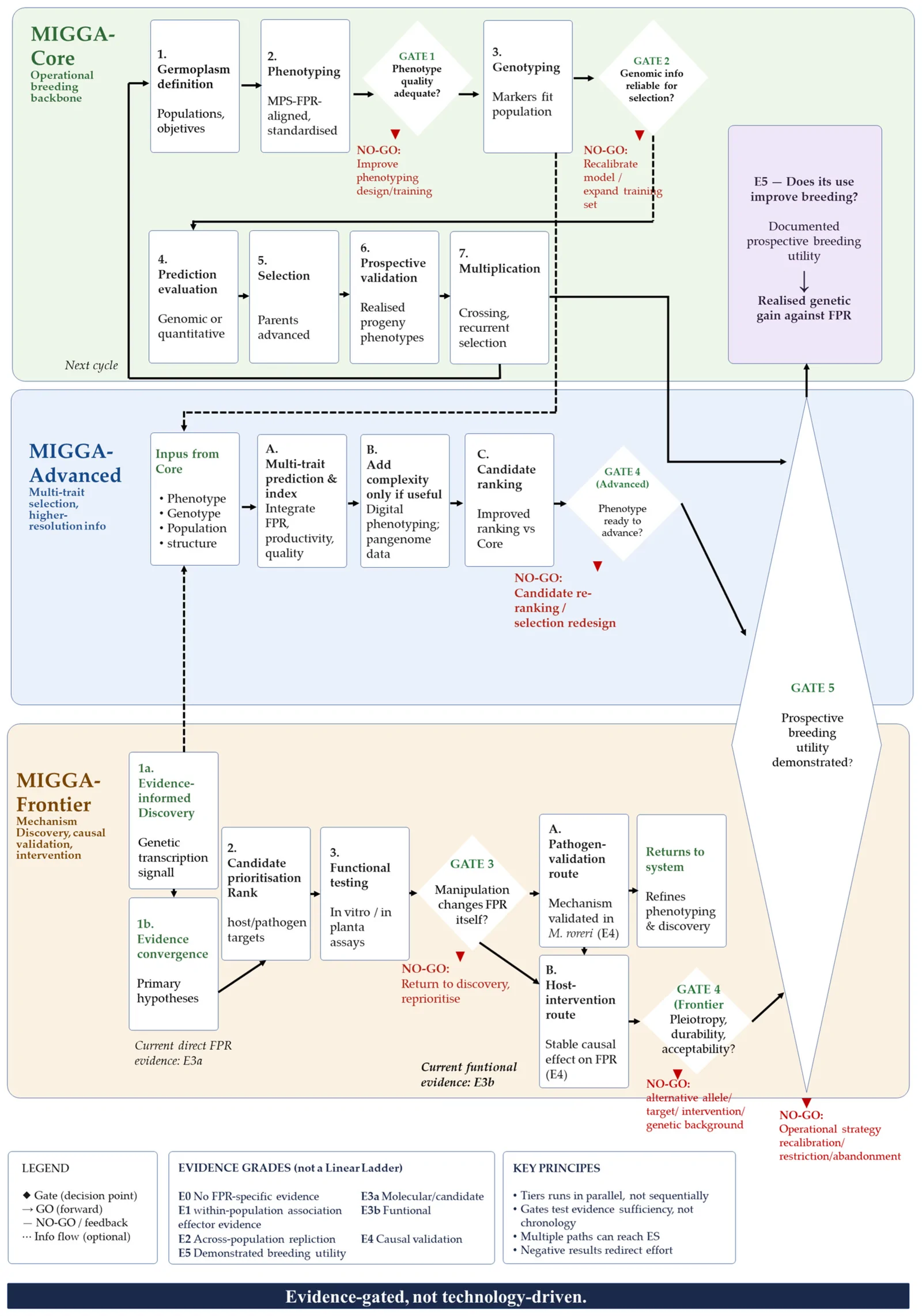
MIGGA represented as a decision map. The framework and its decision points are author-derived proposals that have not been evaluated empirically. Three parallel tiers are shown: MIGGA-Core, the operational breeding backbone; MIGGA-Advanced, multi-trait prediction and higher-resolution information adopted where they add decision value; and MIGGA-Frontier, mechanism discovery, functional testing and causal validation. Five decision points assess phenotype qualification, predictive utility, causal validation, breeding acceptability and prospective breeding utility; their prospective specification is given in Table 9. The pathogen-validation and host-intervention routes are shown separately because only the latter has a direct pathway towards breeding deployment. Evidence classes E0–E5 classify claims (Table 1) and are not equivalent to the decision points. Dashed arrows indicate NO-GO feedback or information return; solid arrows indicate forward progression.

**Figure 3:**
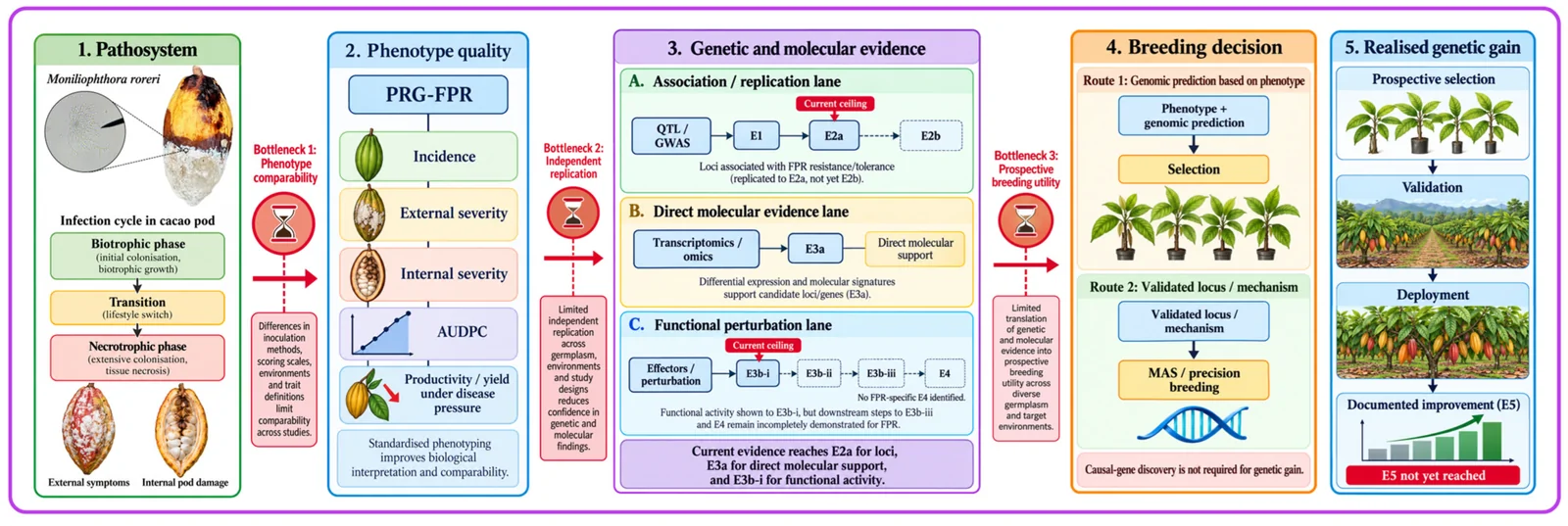
Translational bottlenecks linking frosty pod rot biology, phenotyping, genetic and mechanistic evidence, and realised genetic gain in cacao. The figure integrates the cacao–*M. roreri* pathosystem with PRG-FPR-aligned phenotyping, genetic and molecular evidence, breeding decisions and prospective genetic gain. Solid arrows denote evidence-supported transitions, whereas dashed arrows indicate transitions that remain incomplete or undemonstrated. Current evidence reaches E2a for locus replication and E3b-i for functional activity, while FPR-specific E4 causal validation and E5 breeding utility were not identified in the retrieved and verified literature. This is an author-derived conceptual synthesis and not a validated breeding pipeline.

**Table 1:** Author-developed, multidimensional evidence classification system for FPR resistance claims. The classification is specific to this review and is not an established external evidence standard. Codes label claim types on separate axes and do not form a single ordinal scale of evidentiary strength.

Axis	Class	Question Answered	Operational Criterion Applied to FPR Claims
Directness qualifier	E0	Is the claim supported by data from the cacao–*M. roreri* interaction?	Claim invoked for FPR solely on evidence from another pathosystem, species or disease (e.g., WBD, *Phytophthora* spp., model plants). The underlying evidence keeps its own class for its own system: TcNPR3 is E4 for *Phytophthora* but E0 for FPR.
Genetic (association and replication)	E1	Is there a statistical or positional association in one population or dataset?	QTL, GWAS signal, marker association or positional candidate detected in one population. Re-analysis of the same or an overlapping dataset with another method remains E1.
	E2a	Has the association been re-detected in genetically dependent material?	Re-detection, refinement or fine-mapping in material sharing individuals, parents or the same breeding lineage with the discovery population (same F1 family, half-sib families, overlapping panels). E2a indicates reproducibility within a genetic background, not independent confirmation.
	E2b	Has the association been replicated in genetically independent material?	Same locus or interval, same direction of effect and a comparable phenotype in a population with no shared individuals and no shared parents with the discovery material, with relatedness documented by pedigree or genomic relationship. Related populations derived from overlapping parents are classified as partially dependent and reported as such.
Mechanistic (molecular inference)	E3a	Is a gene, protein, metabolite or pathway associated with the FPR interaction?	Correlative molecular evidence measured in the cacao–*M. roreri* interaction: differential expression, co-expression, network centrality, proteomic or metabolomic change, or machine learning prioritisation of measured transcripts.
	E3b-i	Does the purified molecule have biological activity?	Exogenous application of a recombinant or purified product, e.g., protein spraying or infiltration. Demonstrates biochemical or cellular activity; the context is recorded as heterologous plant, cacao non-target tissue, or cacao pod.
	E3b-ii	Does expression or transient perturbation modulate host responses?	Transient or heterologous in planta expression, or transient perturbation such as agroinfiltration, transient silencing or transient editing, producing a molecular, cellular or tissue phenotype. Demonstrates host-response modulation, not contribution to disease.
	E3b-iii	Does perturbing the endogenous determinant alter infection?	Non-stable perturbation of the endogenous host or pathogen determinant during infection of cacao pods by *M. roreri* (transient knock-down, host-induced gene silencing, specific chemical inhibition) that changes a defined FPR component. No FPR example identified in the literature retrieved.
	E4	Does stable genetic manipulation alter FPR?	Stable, heritable manipulation of a host determinant (stably transformed or edited cacao lines or their progeny) or of a pathogen determinant (stable knockout, knock-in or complementation in *M. roreri*) producing a reproducible directional change in at least one defined FPR component in a cacao pod–*M. roreri* challenge assay, supported by independent transformation events or pathogen strains and, where applicable, genetic complementation. Pods may be attached, or detached if detached-pod responses are validated against attached pods; whole-plant phenotyping is not required. No FPR example identified in the literature retrieved.
Translational (breeding utility)	Validation descriptor	How far has a predictive claim been validated?	Recorded, not graded: random cross-validation within a population; family-, cycle- or environment-structured validation; external validation in another population, with its relatedness stated; forward (prospective) prediction of unphenotyped candidates. The statistic used (predictive ability, accuracy, rank correlation) is always specified.
	E5	Has using the information improved a breeding decision?	Prospective use of a marker, model, index or intervention to make an operational selection or deployment decision, evaluated against a pre-specified comparator and outcome, with documented improvement. No FPR example identified in the literature retrieved.

Robustness descriptors reported for every claim, independently of its class: population size and structure; relatedness between discovery and validation material; phenotype definition (Table 2); statistical model; multiple-testing control; effect size and its uncertainty; and the analytical evidence type, distinguishing a genome-wide significance test from machine learning variable importance. WBD, witches’ broom disease.

**Table 2:** Proposed Phenotyping and Reporting Guideline for frosty pod rot genetic studies (PRG-FPR), organised in modules.

Module	Item	What to Report	Rationale
A. General (all studies)	A1. Host material	Genotype/clone/accession identity, source and, where known, pedigree or genetic group; propagation method and tree age; health and management status of the evaluated material, including known virus or other infections	Unambiguous germplasm identification; enables independent replication and relatedness assessment. Unrelated infections can alter estimated genetic parameters [7]
	A2. Pathogen context	Isolate identity and provenance (controlled studies) or description of natural inoculum (field studies); molecular genetic group where available; molecular confirmation of *M. roreri* on a subsample of diseased pods	Links host rankings to the pathogen material; lineage is recorded without assuming that it predicts aggressiveness [8]
	A3. Phenotype definition	Each disease component measured (incidence; external severity; internal severity; incubation period; time to sporulation; disease progress), the scale used and its reference, and the timing of assessment; productivity under disease pressure when tolerance is claimed	Components are biologically non-equivalent and must not be pooled under a generic “resistance” label
	A4. Design hierarchy	Unit of observation (pod) and unit of replication (tree, plot); nesting of pods within trees, clones, blocks, sites and years; number of units at each level; randomisation and blocking	Prevents pseudoreplication [9]; determines the precision of genotype estimates
	A5. Reference genotypes	Susceptible and partially resistant check genotypes and their observed responses	Internal calibration; no universal differential clone has been validated
	A6. Scoring reliability	Number and training of scorers; blinding to genotype; inter- and intra-rater reliability on a calibration subset, e.g., intraclass correlation or concordance correlation	Quantifies measurement error in visual severity scores [10]
	A7. Statistical model	Distribution and link (binomial or beta-binomial GLMM, cumulative-link model, time-to-event model, linear mixed model), random-effect structure, treatment of repeated measures and spatial variation; scale (observed or latent) and basis (individual or entry mean) of heritability or repeatability	Makes estimates comparable and interpretable (Section 3.5)
	A8. Data availability	Pod-, tree- and plot-level data with metadata in a public repository	Enables re-analysis and meta-analysis [11]
B. Controlled challenge	B1. Inoculum	Conidial concentration (conidia mL^−1^); viability (% germination on the day of inoculation); source and age of conidia; number of isolates	Separates host response from inoculum dose and quality
	B2. Pods and inoculation	Physiological pod age (weeks after pollination or length); attached or detached pods; method, site and volume of inoculation; post-inoculation humidity treatment	Pod age and inoculation method strongly affect infection
	B3. Environment and schedule	Temperature and relative humidity during incubation; assessment dates relative to inoculation; mock-inoculated controls	Conditions germination, colonisation and symptom timing
C. Field and natural infection	C1. Context	Site, season and year, plant age, shade and sanitation regime	Defines the epidemic and management context of the phenotype
	C2. Disease pressure	Incidence in check or border trees; relevant weather variables	Distinguishes resistance from escape under low pressure
	C3. Pod-counting protocol	Frequency of harvest and removal; definition of diseased and healthy pods; treatment of pods lost, removed early or still asymptomatic (censoring)	Determines what incidence actually measures
	C4. Denominator and layout	Total pods and yield per tree reported separately from incidence; tree coordinates for spatial modelling	Avoids ratio artefacts; enables spatial adjustment [12]

PRG-FPR is an author-derived proposal informed by the reviewed studies and by minimum-information initiatives in other fields; it has not been validated by inter-laboratory testing. Module A applies to all studies, Module B only to controlled-challenge experiments and Module C only to field studies. Numerical values are deliberately not prescribed. GLMM, generalised linear mixed model.

**Table 3:** Summary of the principal direct genetic and genomic evidence for frosty pod rot resistance in cacao.

Study	Population/Design	Phenotype; Analysis and Statistic	Principal FPR Finding	Class	Key Limitation
Brown et al. 2007 [15]	256 F1, Pound 7 × UF273, CATIE	Pod inoculation; external and internal pod resistance; interval mapping	Five FPR QTL on linkage groups 2, 7 and 8	E1 → E2a for re-detected loci *	Confirmation derives from later work on the same pedigree
Gutiérrez et al. 2021 [16]	179 F1 genotyped, 169 phenotyped; same Pound 7 × UF273 pedigree; dense SNP map	Attached pods sprayed with conidia; external (ES) and internal (IS) severity on 0–5 scales nine weeks after inoculation	Eleven QTL (five ES, six IS); four earlier signals re-detected, seven newly reported	E2a */E1 †	Re-detected loci remain within one pedigree; inoculum metadata incomplete
Romero Navarro et al. 2017, GWAS [17]	148 improved clones, CATIE (Costa Rica); eight years of natural infection	Proportion of diseased pods from monthly counts; mixed-model GWAS; Bonferroni threshold (α = 0.05)	Six significant SNPs on chromosomes 4, 5, 6 and 10	E1	No locus-level replication
Romero Navarro et al. 2017, genomic prediction [17]	Same 148-clone panel	GBLUP with VanRaden kinship [18]; five-fold cross-validation repeated 50 times; predictive ability r(GEBV, observed)	Predictive ability 0.60 (90 markers) to 0.67 (all markers)	Random CV within population; no E5	Not externally validated; related improved clones may inflate CV estimates [19]
Romero Navarro et al. 2017, linkage [17]	Pound 7 × UF273 “Type 2” family (n = 68), a dataset separate from the GWAS panel; “Type 1” family (n = 180) showed no significant FPR QTL	Same field phenotype; single-family linkage analysis	One chromosome-5 QTL explaining 28.8% of phenotypic variation	E1	Small family, so the effect is probably overestimated [20]; shares the Pound 7 parent with the Brown–Gutiérrez pedigree
Osorio-Guarín et al. 2020 [21]	102 accessions, Colombia	AUDPC from weekly field records; mixed-model GWAS	Chromosome-1 signal near an S-locus lectin-kinase-family gene	E1	Nominal association did not survive FDR correction
Ahn et al. 2025 [22]	Re-analysis of the 102-accession dataset of [21]	Machine learning variable importance (bootstrap forest, boosted tree)	Ranking of FPR-associated genomic features and candidate genes	E1	Variable importance is not a significance test; same dataset; limited out-of-sample performance
McElroy et al. 2018 [23]	Three related Ecuadorian populations derived from overlapping parents: Las Tecas (n = 589), Malvinas (n = 385), Ganadería (n = 391)	Monilia-infected pods as % of total pods (annual counts), adjusted means; GWAS; GBLUP trained on one population and applied to the other two; predictive ability r(prediction, adjusted mean)	Population-specific GWAS signals; mean across-population predictive ability ≈ 0.16	E1; external validation in partially dependent populations	Markers did not replicate across populations; low transferability
Solís Bonilla et al. 2021 [24]; 2024 [12]	Two independent Mexican full-sib progeny trials	Incidence per tree over four years; individual-tree mixed models with spatial terms	Narrow-sense heritability ≈ 0.36–0.46, depending on model	Not graded ‡	Population- and model-specific; no locus-level claims

* E2a applies only to loci re-detected across Brown et al. [15] and Gutiérrez et al. [16] within the same Pound 7 × UF273 pedigree. † Newly identified Gutiérrez et al. [16] QTL remain E1 until confirmed in independent material. ‡ Heritability estimates show that the phenotype is selectable but are not locus-, candidate- or mechanism-level claims and are therefore not classified. Predictive ability is the correlation between predicted and observed (or adjusted) phenotypes; it is not accuracy, which refers to the correlation with true genetic values. AUDPC, area under the disease progress curve; CV, cross-validation; FDR, false discovery rate; GBLUP, genomic best linear unbiased prediction; GEBV, genomic estimated breeding value.

**Table 4:** Representative clone-evaluation and breeding-trial evidence relevant to frosty pod rot (FPR) resistance, identified by the broadened search.

Study	Material and Environment	FPR Phenotype	Finding Relevant to Genetic Gain	Principal Limitation
Phillips-Mora et al. 2005 [8]	Five clones × seven Colombian isolates from four pathogen genetic groups; artificial pod inoculation	Incidence; external and internal severity	ICS-95 comparatively resistant to all isolates; five isolates highly aggressive across hosts; genetic group did not predict aggressiveness	Few host genotypes; one environment
Jaimez et al. 2020 [25]	Cacao clones evaluated under natural pod-rot pressure in Ecuador	Healthy and diseased pod counts combined into a disease and production index (DPI)	Productivity and disease tolerance were not aligned; the DPI ranked clones on a combined objective	Composite index conflates components; natural inoculum
López Torres et al. 2025 [26]	40 clones (20 CATIE, 20 FHIA); randomised complete blocks, four replicates, Honduras, 2013–2017	Percentage of diseased pods (weekly); yield; DPI	Highest-yielding clones were not always the most resistant; combined criteria identified clones combining yield with reduced FPR and black pod	Single site; natural inoculum; index-based ranking
Vanegas et al. 2025 [27]	18 clones; artificial inoculation and field evaluation under agroforestry and full sun, Colombian Amazon	External and internal symptoms; incidence; DPI	Clone FEC-2 showed no external or internal symptoms and the lowest incidence in both production systems	One region; isolate metadata limited
Avendaño-Arrazate et al. 2025 [28]	INIFAP and introduced clones selected partly for FPR tolerance, multiple Mexican environments	Productivity and agronomic traits	Significant genotype × environment interaction for clone performance	FPR not the primary measured phenotype
Villamizar-Gallardo et al. 2019 [29]	Six biological materials (two hybrids and the clones ICS 95, IMC 67, CCN 51 and a TSH selection) surveyed on four farms in Norte de Santander, Colombia	Incidence as diseased pods/(diseased + healthy) pods, sampling 10% of the plants per hectare	FPR incidence 0.4–21.6% across sites; hybrids and the TSH selection most affected, lowest incidence in CCN 51, ICS 95 and IMC 67; nine *M. roreri* isolates recovered, differing morphologically	Survey design: material confounded with farm, no replication structure or genotype-level statistical test, and clone designations reported inconsistently within the source

These studies provide phenotypic and quantitative evidence of selectable variation and genotype × environment interaction; they make no locus- or mechanism-level claims and are therefore not assigned E-codes. Terminology (“tolerant”, “resistant”) follows the primary studies. CATIE, Centro Agronómico Tropical de Investigación y Enseñanza; FHIA, Fundación Hondureña de Investigación Agrícola; INIFAP, Instituto Nacional de Investigaciones Forestales, Agrícolas y Pecuarias.

**Table 5:** Representative candidate and mechanistic evidence relevant to frosty pod rot resistance in cacao.

Candidate or Evidence Category	Genome; Direct Pathosystem	Evidence Supporting the Claim	FPR Class	Principal Limitation
S-locus lectin-kinase-family candidate [21]	Host; FPR	Positional candidate near the chromosome-1 FPR GWAS signal	E1	Association nominal and not significant after FDR correction; no replication or functional evidence
GWAS-associated candidate set [17]	Host; FPR	Six significant FPR-associated SNPs, with nearby genes annotated as trithorax-like, phosphate transporter, γ-tocopherol methyltransferase and kinases	E1	Unreplicated; early expression differences in the same study were driven mainly by cultivar rather than inoculation
ML-prioritised candidate set [22]	Host; FPR	Genomic features ranked by bootstrap-forest/boosted-tree importance in the dataset of [21]	E1	Same 102-accession dataset; importance is not a significance test
JA/ET/SA-associated defence programme [36]	Host; FPR	RNA-Seq of “tolerant” and susceptible clones during *M. roreri* infection, with qRT-PCR of selected genes	E3a	Pathway- and expression-level; no individual host gene perturbed
Pathogen expression programme during infection of “tolerant” cacao [37]	Pathogen; FPR	873 *M. roreri* genes differentially expressed among host backgrounds; 43 validated by qRT-PCR	E3a	Correlative; no gene perturbed
Secreted-protein, effector and CWDE repertoire [38]	Pathogen; FPR	Genome/secretome analysis and phase-associated expression across the biotrophic–necrotrophic transition	E3a (category level)	Resolves functional classes, not individual virulence genes
ML-prioritised fungal regulatory hubs (methyltransferase, Hsp20) [39]	Pathogen; FPR	Network and ML analysis of infection-associated transcripts from contrasting hosts	E3a (hypothesis-generating)	Computational ranking; no perturbation
Three candidate effectors [34]	Pathogen; FPR-derived candidates	Transcriptomic prioritisation; recombinant proteins sprayed on tomato (Micro-Tom) seedlings caused leaf drying, wilting and necrosis	E3b-i (heterologous plant)	Activity shown in a non-host species; no test in cacao and no genetic perturbation in *M. roreri*
MrB1/BASIDIN1 effector [35]	Pathogen; FPR	Recombinant MrB1 disrupted *N. benthamiana* cells and caused genotype-dependent necrosis on cacao leaves; localised necrosis in cacao fruits	E3b-i (cacao leaf and fruit)	Exogenous protein only; no knockout, complementation or host manipulation; contribution to disease not tested
MLO-like protein [21]	Host; WBD directly	WBD-associated candidate; susceptibility-gene analogy from other pathosystems [40]	E0 for FPR	No FPR association, expression or functional test
TcNPR3 [41,42]	Host; *Phytophthora* spp. directly	Transient perturbation followed by stable, heritable CRISPR–Cas9 mutagenesis reducing *Phytophthora* lesion size	E0 for FPR	*M. roreri* not tested

Classes follow Table 1; E3b subclasses indicate the type of perturbation and, in parentheses, its biological context. Quotation marks indicate terminology of the primary study. CWDE, cell-wall-degrading enzyme; ET, ethylene; FDR, false discovery rate; JA, jasmonic acid; ML, machine learning; SA, salicylic acid; WBD, witches’ broom disease.

**Table 6:** Comparison of phenotyping approaches underlying the principal FPR genetic and genomic studies in cacao.

Study	Phenotyping Design	FPR Phenotype	Temporal Structure	Principal Strength	Principal Limitation for Cross-Study Comparison
Brown et al. 2007 [15]	Pound 7 × UF273 F1 population; controlled pod-inoculation phenotype used for QTL mapping	External and internal pod resistance	Repeated pod-level evaluation underlying genotype means	Direct experimental challenge of a controlled segregating population	Full inoculation and scoring metadata are less completely recoverable from the accessible primary report than in the later study of the same pedigree
Gutiérrez et al. 2021 [16]	Young attached pods, 2–3 months old, sprayed with *M. roreri* spore suspension; bagged for 2 days to maintain humidity; repeated inoculations over four years	External severity (ES) and internal severity (IS), each scored on defined 0–5 scales at 9 weeks after inoculation	Each genotype inoculated on 3–17 occasions depending on fruit availability; ≥5 fruits and ≥3 inoculation events required for analysis	Most explicitly standardised controlled FPR phenotype among the mapping studies; separates external from internal disease	Number of pods/inoculation events varied among genotypes; inoculum concentration and isolate-level metadata are not reported with the level recommended by the proposed PRG-FPR
Osorio-Guarín et al. 2020 [21]	Field evaluation of 102 accessions; healthy and diseased pods recorded weekly using 2–6 clonal trees per accession over four harvest periods	FPR disease progress summarised using AUDPC	September 2016–May 2018; repeated weekly field observations	Integrates disease expression over time and across genetically diverse accessions	Infection dose, timing and pathogen isolate are not experimentally standardised; field exposure may incorporate spatial and environmental heterogeneity
Romero Navarro et al. 2017 [17]	148 improved clones; replicated single-tree field evaluation; approximately 25 plants per clone; natural endemic FPR pressure	Proportion of pods showing FPR symptoms, derived from healthy and diseased pod counts	Monthly pod counts over eight years (2007–2015)	Exceptional longitudinal depth and large number of pod observations; captures interannual disease variation	Natural disease pressure is not experimentally controlled, and the phenotype integrates environmental and pathogen-population variation with host response
Ahn et al. 2025 [22]	No new phenotyping; re-analysis of the Osorio-Guarín et al. [21] dataset	Same FPR AUDPC phenotype inherited from [21]	Same historical observations as [21]	Enables comparison of computational approaches on an identical genotype–phenotype dataset	Provides no independent phenotypic replication and inherits all limitations of the original field phenotype
Solís Bonilla et al. 2021 [24]	Two full-sib progeny trials at INIFAP, Chiapas; individual trees evaluated under field disease pressure	FPR incidence calculated as diseased pods/total harvested pods per tree	Trees assessed weekly for mature pods from April to November for four consecutive years	Independent breeding population; repeated multi-year incidence and yield data allow joint genetic evaluation	Incidence does not distinguish infection establishment, internal severity, disease progression or sporulation; natural inoculum is not standardised
McElroy et al. 2018 [23]	Three related Ecuadorian breeding populations evaluated under field conditions	Monilia pod incidence expressed relative to total pod production	Multi-year field data; genotype means adjusted for site, year and plant age	Enables both cross-population marker comparison and training-population→test-population genomic prediction	Natural infection and heterogeneous field exposure; populations related rather than fully independent genetic backgrounds
Solís Bonilla et al. 2024 [12]	Spatial analysis of FPR incidence in two INIFAP full-sib progeny trials	Percentage/incidence of FPR together with productivity traits	Four-year field phenotype analysed with B-spline, autoregressive and Bayesian ICAR spatial models	Explicitly quantifies and models within-trial spatial autocorrelation, reducing environmental confounding in genetic-parameter estimation	Improves statistical treatment of the field phenotype but does not standardise pathogen genotype, inoculum dose or developmental stage at infection

**Table 7:** Sources of heterogeneity between the two direct FPR genomic-prediction studies, showing why their reported values are not directly comparable.

Feature	Romero Navarro et al. 2017 [17]	McElroy et al. 2018 [23]
Material	148 improved clones, one Costa Rican site	Three Ecuadorian populations derived from overlapping parents: Las Tecas (n = 589), Malvinas (n = 385), Ganadería (n = 391)
Relatedness, training vs. target	Same population; training and validation individuals drawn at random from one related panel	Target populations not used for training, but related to it through shared parents (partially dependent)
Phenotype	Proportion of diseased pods, monthly counts over eight years	Monilia-infected pods as a percentage of total pods, annual counts; adjusted means from a mixed model
Model and relationship matrix	GBLUP, VanRaden genomic relationship matrix [18]	GBLUP, identity-by-state matrix
Validation design	Five-fold cross-validation repeated 50 times	Whole-population training, applied to two other populations; no cross-validation
Statistic reported	Predictive ability: r(predicted, observed clone value)	r(predicted, adjusted phenotype) in populations not used for training
Value for FPR	0.60 (90 markers) to 0.67 (all markers)	≈0.16 mean across populations not used for training
Question answered	Can the model interpolate among relatives within one characterised population?	Does the model transfer to a different, partially related breeding population?

Neither study used predictions prospectively to make selection decisions, so neither reaches E5. GBLUP, genomic best linear unbiased prediction.

**Table 8:** Approaches relevant to frosty pod rot resistance in cacao, assessed on four separate dimensions.

Approach	Primary Role	Highest Direct FPR Evidence for the Corresponding Claim	Infrastructure and Operational Requirements Currently Met in Cacao Programmes	Demonstrated Effect on a Breeding Decision for FPR	Principal Constraint on Translation
Phenotypic selection	Direct selection on realised phenotype	Not classified *	Yes: established in multiple programmes; clones with reduced FPR released [8,26,27]	Yes, by conventional selection history	Long generation interval; environmentally dependent phenotype
Marker-assisted selection	Selection for specific validated loci	E2a [15,16]	Partly: genotyping available; no validated FPR marker	None identified	No E2b replication; transferability not demonstrated
GWAS/machine learning	Discovery and candidate prioritisation	E1 [17,21,22]	Yes, for discovery purposes	None identified	Association or variable importance is not validation or causality
Genomic selection	Genome-wide prediction of breeding or genotypic value	Within-population predictive ability 0.60–0.67; cross-population ≈ 0.16 [17,23]; no E5	Partly: requires training populations with PRG-FPR-aligned phenotypes and periodic recalibration	None identified; not yet used prospectively	Within-population accuracy does not imply transferability
Integrated multi-omics	Mechanism discovery and evidence convergence	E3a at component level [36,37,38,39]	No integrated host × pathogen × time design implemented for FPR	None identified	Discovery tool, not a selection technology
Effector functional assays	Mechanistic testing	E3b-i [34,35]	Available in specialised laboratories	None identified	Activity of a recombinant protein is not a contribution to disease
Gene editing	Targeted causal intervention and causal validation	E0 for FPR [41,42]	Stable transformation and editing feasible in cacao, in few laboratories	None identified	No validated FPR causal target; long pathway to a deployable product

Assessment basis. Column 3: the highest class, under Table 1, of any claim directly concerning FPR for which this approach has provided evidence. Column 4: whether the germplasm, phenotyping, laboratory and computational infrastructure required for routine use are documented in the reviewed cacao literature. Column 5: whether the reviewed literature documents use of this approach to make an FPR selection or deployment decision. Column 6: the single limitation that most constrains translation at present. The columns are assessed independently and are not combined into an overall score. * Conventional phenotypic selection is not classified because the system in Table 1 grades genetic, molecular and deployment claims rather than direct phenotype-based selection.

**Table 9:** Proposed structure for prospectively specifying each MIGGA decision point. The elements are author-derived; the parameters marked as programme-defined must be fixed before the result is known, and no universal numerical threshold is proposed.

Decision Point	Claim Tested	Comparator	Primary Endpoint	Decision Rule and Uncertainty	Replication /Validation Structure
1. Phenotype qualification	The phenotype is reliable enough for the intended genetic inference	The programme’s current phenotyping protocol	Repeatability or entry-mean heritability of the defined disease component, on a stated scale (Section 3.5)	Advance if the estimate exceeds the programme-defined minimum for the intended use, with its standard error or credible interval reported; the minimum differs for ranking parents, estimating variance components and training a prediction model	Independent experimental runs, seasons or sites; scorer reliability estimated on a calibration subset
2. Predictive qualification	Genomic information improves the selection decision for the target population	Phenotypic, pedigree-based or existing genomic selection as currently practised	Predictive ability in material not used to fit the model, with the validation design stated	Advance if the gain over the comparator exceeds the programme-defined margin, judged against the interval across validation replicates rather than a point estimate; cost and time are part of the comparison	Validation structured to match the intended use: across families, cycles, environments or populations, with relatedness documented
3. Causal validation	Manipulating the determinant changes FPR	Matched non-manipulated control of the same background or parental strain	Change in a pre-specified FPR component in a pod-challenge assay (Table 1, Section 8.3)	Accept the causal claim if the directional change is reproduced and its interval excludes no effect; the biologically relevant magnitude is defined before the experiment	Supported by independent transformation events or pathogen strains and, where applicable, genetic complementation; replication across pods, trees and experimental runs should be determined prospectively by power analysis
4. Breeding acceptability	The favourable phenotype is usable within the whole breeding objective	The programme’s current best material for the same target environment	Performance in each essential trait relative to programme-defined minimum requirements (independent culling levels)	Advance only if every essential trait meets its minimum; the scope of the claim is limited to the backgrounds, isolates and environments actually tested	Multi-environment and, where relevant, multi-isolate and multi-background evaluation matched to the intended deployment claim
5. Prospective breeding utility (E5)	Using the information improved the breeding outcome	The strategy the programme would otherwise have used, specified in advance	A pre-specified breeding outcome: selection accuracy, realised multi-trait performance, cycle time, phenotyping cost or genetic gain per unit time	Adopt if the observed benefit exceeds the pre-specified margin, accounting for the cost and complexity of implementation; outcome and comparator fixed before results are known	Decisions made before the realised phenotypes of validation candidates are known, then evaluated against them

A NO-GO at any point restricts the scope of the claim and identifies the stage to return to; it does not reverse the evidence already obtained. The five points are decision-specific filters, not a compulsory sequence: genomic selection may proceed from point 1 to point 2 and then to point 5 without a causal gene, whereas pathogen-side functional genetics may culminate at point 3 without approaching a breeding product.

**Table 10:** Priority evidence gaps and decisive experiments for FPR resistance in cacao.

Evidence Gap	Current Status	Decisive Experiment	Consequence of the Result
Phenotype comparability	Studies remain heterogeneous in host stage, pathogen material, inoculation and endpoints; PRG-FPR is proposed but not validated across programmes	Inter-laboratory or multi-site ring trial using shared reference genotypes and harmonised disease definitions	Reproducible contrasts support harmonised phenotyping; discordance identifies the design components responsible
Independent genetic replication (E2b)	Strongest FPR QTL evidence reaches E2a within one pedigree [15,16]; no E2b locus identified in the literature retrieved and verified	Pre-specified replication of the leading intervals in genetically independent cacao material, phenotyped for external and internal severity	Replication supports marker evaluation; failure restricts the claim and avoids further investment in non-transferable loci
Prediction transferability	Within-population predictive ability 0.60–0.67 [17] contrasts with ≈0.16 across related populations [23]	Structured cross-family, cross-cycle or cross-population prediction outside the original training domain, with relatedness documented	Supports broader use, or restricts the prediction domain and redirects training-population design
Stable causal validation (E4)	Host evidence mainly E3a; pathogen effector activity E3b-i; no FPR-specific E4 identified in the literature retrieved and verified	Stable manipulation of a prioritised host or pathogen determinant, followed by pod challenge under PRG-FPR (Section 8.3)	Reproducible change establishes causality within the tested context; a null result narrows or rejects the hypothesis
Host × pathogen × environment breadth	Isolate-dependent responses documented; breadth cannot be inferred from one combination	Connected multi-host × multi-isolate × multi-environment validation of selected genotypes or validated interventions	Broadens or restricts the deployment claim
Prospective breeding utility (E5)	No FPR marker, model or intervention identified in the literature retrieved and verified has demonstrated E5	Prospective selection experiment comparing the evidence-informed strategy with the programme’s realistic current practice	Documented benefit establishes E5 for that context; absence of benefit withholds adoption while retaining the underlying evidence

The experiments address distinct claims and are not a compulsory sequence.

## Data Availability

No new primary experimental data were generated in this study. All evidence synthesised in this review was derived from the published sources cited in the manuscript. The curated evidence products supporting the review, including the detailed Evidence Atlas, search framework, candidate and mechanistic evidence matrix, due-diligence records and experimental research-agenda specifications, are provided in the Supplementary Materials (Tables S1A–S5). Additional information supporting individual evidence classifications can be traced to the original publications cited in the manuscript.

## Supplementary Materials

The following supporting information can be downloaded at https://www.mdpi.com/article/10.3390/ijms27209294/s1.

## Abbreviations

The following abbreviations are used in this manuscript:

FPR	Frosty pod rot
WBD	Witches’ broom disease
QTL	Quantitative trait locus
GWAS	Genome-wide association study
GS	Genomic selection
MAS	Marker-assisted selection
SNP	Single-nucleotide polymorphism
AUDPC	Area under the disease progress curve
PRG-FPR	Phenotyping and Reporting Guideline for frosty pod rot
MIGGA	Marco Integrador de Ganancia Genética Acelerada (Integrated Framework for Accelerated Genetic Gain)
OVM/LMO	Organismo Vivo Modificado/Living Modified Organism
