# Supplementary material for: From Phenotype to Genome: A Critical Evidence-Based Synthesis of Frosty Pod Rot (Moniliophthora roreri) Resistance in Cacao and a Proposed Evidence-Gated Framework to Support Genetic Gain
Source: Int J Mol Sci. 2026 Oct 19;27(20):9294. doi: 10.3390/ijms27209294 (PMC13705398; doi:10.3390/ijms27209294)
Supplement: Supplementary file 1 [file ijms-27-09294-s001.zip › ijms-4574815-supplementary.pdf]

Supplementary Materials

From Phenotype to Genome: A Critical Evidence-Based Synthesis of Frosty Pod Rot (*Moniliophthora roreri*) Resistance in Cacao and a Proposed Evidence-Gated Framework to Support Genetic Gain

Original search and evidence-verification cut-off: 30 August 2026. Manuscript consolidation and formatting: 31 August 2026. Complementary update search during peer review: completed 1 October 2026.

Scope. This supplementary package contains the evidence products supporting the revised manuscript: the detailed Evidence Atlas (Tables S1A–S1C), the documented original search framework and prospectively documented complementary update-search record (Table S2), the detailed candidate and mechanistic evidence matrix (Table S3), due-diligence leads not incorporated as settled evidence (Table S4), and experimental specifications for the revised FPR research agenda (Table S5). The evidence-classification data dictionary follows Table S5. The original search was completed on 30 August 2026; the complementary update search was completed on 1 October 2026.

Citation-control note. Citation numbers in this supplementary package have been synchronised with the reference list of the current revised main manuscript. Records in Table S4 remain due-diligence leads and are not promoted as settled evidence unless primary-source verification supports their inclusion.

Supplementary Table S1A. Detailed Evidence Atlas—direct FPR genetic/genomic and quantitative-genetic evidence.

Study / citation	Country / population or design	n	Reference genome	Marker/QTL / chromosome	Genomic position / interval	FPR phenotype	Effect / statistical support	Model / correction	Validation / evidence level	DOI
Brown et al. 2007 [13]	Costa Rica (CATIE); F1 Pound 7 × UF273	256 F1	Pre-genome linkage map	Five FPR QTL; linkage groups 2, 7 and 8	Linkage-map positions; exact interval/effect metadata not fully recoverable in the accessible source used for this synthesis	External and internal pod resistance under controlled challenge	Five FPR QTL reported	Biparental linkage design; population-structure correction not applicable	E1 at discovery; selected loci reach E2a through re-detection/refinement by Gutiérrez et al. 2021 [14] in the same pedigree. Not E2b.	10.2135/cropsci2006.11.0753
Gutiérrez et al. 2021 [14]	Costa Rica (CATIE); same Pound 7 × UF273 F1 pedigree; denser SNP map	179 genotyped; 169 retained for FPR phenotyping	Matina 1-6 v1.1	FPR-ES-1…5 and FPR-IS-1…6; chromosomes 2, 4, 7, 8, 9 and 10	Example: FPR-ES-1 on Chr4, ~9.3 cM; peak SNP Tcm004s01757744	External severity (ES) and internal severity (IS), 0–5 scales, 9 weeks after inoculation	R^2 ≈4.96–11.19%; LOD thresholds ≈3.87–4.50; 11 FPR QTL total; seven newly reported	Biparental linkage design; population-structure correction not applicable	Re-detected loci = E2a within the same pedigree; newly reported loci = E1 until independent replication. No E2b.	10.1007/s11295-021-01507-w
Osorio-Guarín et al. 2020 [16]	Colombia; disease GWAS subset of 102 accessions	102	Criollo B97-61/B2 v2 and Matina 1-6 v1.1	S1_18354976 (Criollo) / S1_27025499 (Matina); Chr1	~11.4 kb upstream of an S-locus lectin-kinase-family gene	FPR disease progress summarised by AUDPC under field exposure	Nominal p ≈$2×10^{-4}$ / $4×10^{-4}$; FPR signal did not survive FDR correction	MLM (GAPIT); STRUCTURE Q-matrix + Loiselle kinship; FDR applied	No independent replication; E1, with reduced confidence because the signal did not survive FDR	10.1534/g3.120.401153
Romero Navarro et al. 2017 [15] — GWAS/GP	Costa Rica (CATIE); 148 improved clones with parentage from Peru, Colombia and Ecuador	148	Matina 1.6	TCM_020057, TCM_020262 (Chr4); TCM_025566, TCM_025986 (Chr5); TCM_027463 (Chr6); TCM_045155 (Chr10)	Gene-model level; no harmonised bp/cM interval reported for all associated sites in the source used here	Proportion of pods showing FPR symptoms during an 8-year field trial	Six significant FPR-associated sites; within-population genomic predictive ability ≈0.61–0.67	GAPIT with VanRaden kinship and admixture coefficients; five-fold cross-validation for prediction	No locus-level independent replication; prediction validated only within the study population; E1	10.3389/fpls.2017.01905
Romero Navarro et al. 2017 [15] — Type 2 linkage family	Pound 7 × UF273 Type 2 linkage family	Small family; n not reproduced here because the current verified synthesis treats this as a distinct sub-analysis	Matina 1.6	Chromosome-5 linkage QTL	Chr5 linkage interval; exact harmonised bp interval not reported in the current synthesis	FPR field resistance phenotype	QTL explained 28.8% of phenotypic variation	Biparental linkage analysis	No independent replication of the chromosome-5 signal; E1	10.3389/fpls.2017.01905
Ahn et al. 2025 [17]	Colombia; confirmed re-analysis of the same 102-accession Osorio-Guarín et al. 2020 panel	102	Criollo B97-61/B2 v2 and Matina 1-6 v1.1	FPR top gene models: Tc03v2_g009640 / Thecc1EG014511 (Chr3); Tc09v2_g017680 and Thecc1EG027776 (Chr9)	Gene-model level	Inherited FPR AUDPC phenotype from Osorio-Guarín et al. [16]	Bootstrap Forest / Boosted Tree 'Portion' variable importance; training R^2 >0.80–0.90, but 5-fold CV R^2 mostly ≤0.20	No mixed-model kinship/Q-matrix correction or FDR/Bonferroni significance threshold; feature importance is not a formal GWAS test	Same dataset as [16], therefore no biological replication; E1	10.1002/tpg2.70069
McElroy et al. 2018 [61]	Ecuador; three related breeding populations (Las Tecas, Ganaderia and Malvinas)	≈1,345 accessions total	Cacao SNP-array framework (15K SNP array)	Population-specific disease-associated SNPs; no FPR marker reproduced consistently across populations	Population-specific marker positions; no transferable FPR locus identified across all populations	Monilia pod incidence relative to total pod production; multi-year field data	Cross-population genomic prediction mean accuracy ≈0.15 for monilia pod	GWAS within populations plus training-population → test-population genomic prediction	Explicit cross-population testing did not yield consistent marker replication; E1 and not E2b	10.3389/fpls.2018.00343
Solís Bonilla et al. 2021 [51]	Mexico (INIFAP, Chiapas); two full-sib progeny trials	Independent full-sib trials	Not locus-level	Not applicable — quantitative-genetic study	Not applicable	FPR incidence = diseased pods / total harvested pods per tree; weekly observations across four years	Narrow-sense heritability ≈0.46	Quantitative-genetic field analysis	Not locus-level graded; supports selectability but not a specific E0–E5 locus/candidate claim	10.1007/s11295-021-01498-8
Solís Bonilla et al. 2024 [41]	Mexico (INIFAP, Chiapas); spatial re-analysis of two full-sib progeny trials	Independent full-sib trials	Not locus-level	Not applicable — quantitative-genetic/spatial study	Not applicable	FPR incidence together with productivity traits; four-year field phenotype	Narrow-sense heritability ≈0.36–0.42 depending on spatial model	B-spline, autoregressive and Bayesian ICAR spatial models	Not locus-level graded; demonstrates the importance of spatial	10.1007/s11295-024-01677-3

Study / citation	Country / population or design	n	Reference genome	Marker/QTL / chromosome	Genomic position / interval	FPR phenotype	Effect / statistical support	Model / correction	Validation / evidence level	DOI
									modelling for genetic-parameter estimation	

Notes: Evidence classes are claim-specific and multidimensional. Brown et al. [13] is E1 at discovery, with selected loci reaching E2a only through later same-pedigree re-detection/refinement in Gutiérrez et al. [14]. Newly reported Gutiérrez et al. [14] loci remain E1. The Solís Bonilla studies [51,41] provide quantitative-genetic evidence for selectability but are not assigned a locus-level genetic evidence class unless a specific locus claim is being evaluated.

Supplementary Table S1B. Direct witches' broom disease (WBD) comparator evidence retained separately from FPR.

Study / citation	Country / population	n	Reference genome / map	Marker/QTL / chromosome	Candidate / category	Evidence type	Validation status	Evidence level	DOI
Osorio-Guarín et al. 2020 [16] — WBD DMI1-type ion channel	Colombia; same disease-resistance panel	102	Criollo / Matina	Multiple SNP; Chr2	DMI1-type ion channel	GWAS association	No independent replication identified	E1 for WBD; E0 if extrapolated to FPR	10.1534/g3.120.401153
Osorio-Guarín et al. 2020 [16] — WBD xyloglucan galactosyltransferase	Colombia; same disease-resistance panel	102	Criollo / Matina	Multiple SNP; Chr2	Xyloglucan galactosyltransferase	GWAS association	No independent replication identified	E1 for WBD	10.1534/g3.120.401153
Osorio-Guarín et al. 2020 [16] — WBD MLO-like protein	Colombia; same disease-resistance panel	102	Criollo / Matina	Multiple SNP; Chr2	MLO-like protein	GWAS association	No independent replication identified	E1 for WBD; E0 when invoked for FPR	10.1534/g3.120.401153
Osorio-Guarín et al. 2020 [16] — WBD IWS1	Colombia; same disease-resistance panel	102	Criollo / Matina	Multiple SNP; Chr3	IWS1 protein	GWAS association	No independent replication identified	E1 for WBD	10.1534/g3.120.401153
Queiroz et al. 2003 [9]	Brazil (Bahia); F2 Scavina-6 × ICS-1	n not reported in the accessible source used here	Pre-genome RAPD/AFLP linkage map	AV14.940 (AFLP); linkage group 11	No positional candidate resolved	Linkage QTL; R^2 ≈35%	Independent replication status ambiguous; related later reports do not map cleanly to the same locus	E1	10.1046/j.1439-0523.2003.00809.x
Chia Wong et al. 2022 [10]	F2 progeny	Not reproduced in current synthesis	High-density SNP linkage map	High-density SNP QTL; chromosome/interval not reproduced in the current synthesis	No positional candidate resolved in the Evidence Atlas used here	Linkage QTL	No independent replication identified	E1	10.1007/s11295-022-01562-x

Supplementary Table S1C. Cross-pathosystem extrapolations relevant to FPR.

Proposed FPR extrapolation	Direct evidence it actually rests on	FPR evidence level	Why it remains E0 for FPR	Source citation(s) / DOI
MLO as an FPR susceptibility-gene candidate	MLO is a validated susceptibility-gene concept in cereal powdery mildew; in cacao, an MLO-like protein was associated with WBD, not FPR [16].	E0	No direct FPR association, expression or functional test against M. roreri has been verified.	[16] 10.1534/g3.120.401153
Earlier-and-more-sustained jasmonate-pathway activation as an FPR kinetic signature	Earlier and more sustained jasmonate-biosynthesis activation was reported in cacao genotypes contrasting for WBD response [68]. Direct FPR host transcriptomics [52] supports JA/ET/SA pathway activation, but not this specific temporal-kinetic claim.	E0 for the kinetic extrapolation only	The pathway itself has direct FPR support (E3a via [52]); the untested element is the earlier-and-more-sustained temporal signature.	[52] 10.1111/mpp.12126; [68] 10.1007/s00299-015-1821-x
TcNPR3 editing as a template for FPR susceptibility-gene editing	Transient and stable manipulation of TcNPR3 alters susceptibility to Phytophthora spp. in cacao [67,23].	E0	Strong functional evidence exists in another cacao pathosystem, but no M. roreri-specific challenge of TcNPR3-edited cacao was identified.	[67] 10.3389/fpls.2018.00268; [23] 10.1111/pbi.70365

Supplementary Table S2. Search framework, Boolean search strings and prospectively documented complementary update search.

ID	Search domain	Core search string / approach	Databases / use	Purpose and methodological note
S1	Pathogen / host / disease	("Moniliophthora roreri" OR "frosty pod rot" OR "frosty pod") AND ("Theobroma cacao" OR cacao OR cocoa)	PubMed/MEDLINE; Web of Science Core Collection; Scopus; Google Scholar	Core discovery string for FPR literature.
S2	Genetics / genomics	S1 AND (QTL OR "quantitative trait loci" OR GWAS OR "genome-wide association" OR "genomic selection" OR "marker-assisted selection" OR heritability OR "candidate gene")	PubMed/MEDLINE; Web of Science; Scopus; Google Scholar	Identify linkage, GWAS, genomic prediction, MAS and quantitative-genetic studies.
S3	Precision breeding	S1 AND (CRISPR OR "gene editing" OR "genome editing" OR "susceptibility gene" OR transgenic)	PubMed/MEDLINE; Web of Science; Scopus; Google Scholar	Identify functional validation, susceptibility-gene and gene-editing evidence; non-FPR pathosystems retained only as explicitly labelled extrapolation.
S4	Phenotyping / methodology	S1 AND (phenotyping OR "inoculation method" OR "disease severity" OR incidence OR AUDPC OR "incubation period")	PubMed/MEDLINE; Web of Science; Scopus; Google Scholar	Recover phenotyping design, disease components and methodological comparability evidence.
S5	Pathogen biology / omics	("Moniliophthora roreri" OR "Moniliophthora perniciosa") AND (genome OR secretome OR transcriptome OR proteome OR effector OR pangenome OR "structural variation")	PubMed/MEDLINE; Web of Science; Scopus; Google Scholar	Recover host/pathogen molecular evidence and WBD comparator studies.
S6	WBD comparator	"Moniliophthora perniciosa" AND ("witches' broom" OR "witches broom") AND cacao	PubMed/MEDLINE; Web of Science; Scopus; Google Scholar	Used only to identify comparator studies, shared datasets or cross-pathosystem hypotheses; WBD evidence was not treated as direct FPR evidence.
Language	Spanish-language discovery	Spanish-language equivalents of S1–S4	Google Scholar and, where supported, Scopus	Supplementary discovery of regional literature; not a separate evidence class.
Method	Citation chaining	Backward/forward reference-list screening of retrieved primary studies and reviews	All retrieved sources	Used to recover primary studies missed by database keywords.
Cut-off	Search date	Original literature search and evidence-verification completed on 30 August 2026; manuscript consolidated/formatted on 31 August 2026; no lower publication-year filter was applied at the original search stage.	All sources	Negative conclusions from the original review are bounded to literature retrieved and verified by 30 August 2026; conclusions in the revised manuscript also incorporate the prospectively documented update completed on 1 October 2026.
Transparency	Design / PRISMA / agreement	No retrospective reconstruction of original per-database hit counts, duplicate-removal counts, PRISMA flow or inter-reviewer κ.	Methodological limitation	Structured critical review, not a formal systematic review. Formal independent duplicate screening/grading was not prospectively implemented.
S2B	Complementary update search during peer review	Expanded generic concept block: ("Moniliophthora roreri" OR "Monilia roreri" OR "frosty pod rot" OR moniliasis OR "moniliophthora pod rot") AND (cacao OR cocoa OR "Theobroma cacao") AND (resist* OR toleran* OR suscept* OR breed* OR clon* OR "combining ability" OR diallel OR progen* OR heritab* OR "genetic parameter*" OR incidence OR severity OR inoculat* OR yield OR productiv* OR "genotype by environment" OR stability OR "disease and production index" OR QTL OR GWAS OR "association mapping" OR "genomic selection" OR "genomic prediction" OR marker* OR transcriptom* OR proteom* OR metabolom* OR effector* OR "candidate gene*" OR CRISPR OR "gene editing").	CAB Abstracts; AGRIS; SciELO; Redalyc; plus re-checking of the original databases where applicable	Prospectively documented update-search block conducted in response to peer review and completed on 1 October 2026. Platform-adapted exact search strings and record counts were transcribed directly from the prospective update-search log; no values were reconstructed or inferred retrospectively.
U1	CAB Abstracts	("Moniliophthora roreri" OR "Monilia roreri" OR "frosty pod rot" OR moniliasis) AND (cacao OR cocoa OR "Theobroma cacao") AND (resist* OR toleran* OR suscept* OR breed* OR QTL OR GWAS OR "genomic selection" OR CRISPR)	Date: 1 October 2026; retrieved: 47; potentially relevant: 12; duplicates: 5; included: 3; excluded: 4	Verifier: YYP-R. Exclusions: 2 records on black pod (Phytophthora spp.) only; 1 review without primary FPR data; 1 record without a defined FPR phenotype.
U2	AGRIS	("Moniliophthora roreri" OR "frosty pod rot" OR moniliasis) AND (cacao OR cocoa) AND (resist* OR progen* OR heritab* OR incidence OR severity OR yield OR "genotype by environment")	Date: 1 October 2026; retrieved: 23; potentially relevant: 7; duplicates: 2; included: 2; excluded: 3	Verifier: YYP-R. Exclusions: 1 agronomic survey without genetic or phenotypic resistance data; 1 conference abstract superseded by a peer-reviewed article; 1 duplicate of a SciELO record.
U3	SciELO	("Moniliophthora roreri" OR "Monilia roreri" OR "moniliasis del cacao" OR "frosty pod rot") AND (cacao OR cocoa OR "Theobroma cacao") AND (resistencia OR tolerancia OR mejoramiento OR QTL OR GWAS OR CRISPR OR "selección genómica")	Date: 1 October 2026; retrieved: 31; potentially relevant: 9; duplicates: 4; included: 3; excluded: 2	Verifier: YYP-R. Exclusions: 1 regional agronomic note without primary data; 1 Spanish-language duplicate of a CAB Abstracts record.
U4	Redalyc	("Moniliophthora roreri" OR "moniliasis" OR "frosty pod rot") AND (cacao OR "Theobroma cacao") AND (resistencia OR tolerancia OR mejoramiento OR incidencia OR severidad OR rendimiento)	Date: 1 October 2026; retrieved: 18; potentially relevant: 5; duplicates: 1; included: 1; excluded: 3	Verifier: YYP-R. Exclusions: 1 record on M. perniciosa only (WBD comparator, not direct FPR); 1 without primary phenotypic data; 1 duplicate of an AGRIS record.

ID	Search domain	Core search string / approach	Databases / use	Purpose and methodological note
U5	Reference-management / deduplication record	Zotero 7.0; dedicated collection: "Cacao Moni IJMS".	Revision documentation; complementary update search completed 1 October 2026.	Reference records from the complementary update search were managed in Zotero 7.0 and deduplicated against the original 30 August 2026 library using the dedicated collection "Cacao Moni IJMS". Duplicate records were reviewed before removal.

Methodological transparency. The original review used PubMed/MEDLINE, Web of Science, Scopus and Google Scholar and was designed as a structured critical review and evidence synthesis rather than a formal systematic review. No protocol was preregistered, no PRISMA-based screening workflow was implemented, and formal independent duplicate screening or duplicate evidence grading was not prospectively planned. Exact per-database retrieval counts, duplicate-removal counts and the complete screening history for the original search were not retained in a form that permits faithful retrospective reconstruction; consequently, no retrospective PRISMA flow diagram, screening counts or Cohen's κ are reported. The original literature search and evidence-verification process was completed on 30 August 2026, and the manuscript was consolidated and formatted on 31 August 2026. Following peer review, a complementary update search was conducted prospectively using expanded database coverage and a broadened breeding-oriented concept block; this update was completed on 1 October 2026. Its database-specific queries, record counts, screening disposition, exclusion reasons and verifier are reported directly from the prospective update-search log in the S2B records above. The original and complementary search stages are reported separately and should not be conflated.

Supplementary Table S3. Detailed candidate and mechanistic evidence matrix.

Candidate / evidence unit	Citation	Genome / direct pathosystem	Gene ID / locus	Annotation / pathway	Evidence type	Direct molecular support	Functional / perturbational support	Independent replication	FPR evidence level	Principal limitation / interpretive caution	DOI
S-locus lectin-kinase-family candidate	[16]	Host; FPR	Near S1_18354976 / S1_27025499, Chr1	Lectin-type receptor-kinase family; positional candidate	GWAS positional evidence	No direct expression evidence required for the E1 claim	None identified	None	E1	Nominal association did not survive FDR correction; no independent replication or functional test.	10.1534/g3.120.401153
Trithorax-like protein 2	[15]	Host; FPR	TCM_020057	Chromatin-modifying / transcriptional regulator	GWAS positional candidate	Expression differences in the same study were attributable primarily to cultivar rather than inoculation treatment	None identified	None	E1	Positional association remains unreplicated; should not be described as an infection-induced mechanism.	10.3389/fpls.2017.01905
Phosphate transporter 1;5	[15]	Host; FPR	TCM_020262	Phosphate homeostasis; nutrient-signalling/defence crosstalk	GWAS positional candidate	No direct infection-responsive support established for this individual gene in the current synthesis	None identified	None	E1	Positional candidate only; function in FPR remains untested.	10.3389/fpls.2017.01905
γ-Tocopherol methyltransferase-linked candidate(s)	[15]	Host; FPR	TCM_025566 / TCM_025986	Vitamin E / antioxidant metabolism	GWAS positional candidate	No direct infection-responsive support established for this individual candidate in the current synthesis	None identified	None	E1	Association is unreplicated and does not establish causal antioxidant function in FPR.	10.3389/fpls.2017.01905
Kinase superfamily candidate	[15]	Host; FPR	TCM_027463	Kinase-family annotation unresolved at finer functional level	GWAS positional candidate	No direct molecular support for an individual causal role	None identified	None	E1	Annotation is too coarse for a mechanistic claim; no independent replication.	10.3389/fpls.2017.01905
Uncharacterised candidate proteins	[15]	Host; FPR	TCM_045155-linked models	Uncharacterised	GWAS positional candidate	No direct molecular support for individual causal roles	None identified	None	E1	Functional annotation and causal role unresolved.	10.3389/fpls.2017.01905
Galactinol-sucrose galactosyltransferase models	[17]	Host; FPR	Tc03v2_g009640 / Thecc1EG014511	Sugar / raffinose-family metabolism	ML feature importance	Feature measured from genotype–phenotype re-analysis, not transcript perturbation	None identified	None; same dataset as [16]	E1	Variable importance is not a formal GWAS association; no kinship/Q-matrix/FDR framework and low 5-fold out-of-sample R^2.	10.1002/tpg2.70069
DEAD-box RNA helicase 37	[17]	Host; FPR	Tc09v2_g017680	RNA metabolism / stress-response-related annotation	ML feature importance	Feature measured from genotype–phenotype re-analysis	None identified	None; same dataset as [16]	E1	Same methodological limitations as other [17] ML-ranked features.	10.1002/tpg2.70069
Troponin-like coiled-coil domain protein	[17]	Host; FPR	Thecc1EG027776	Structural / unresolved annotation	ML feature importance	Feature measured from genotype–phenotype re-analysis	None identified	None; same dataset as [16]	E1	Same methodological limitations as other [17] ML-ranked features.	10.1002/tpg2.70069
FPR-ES/FPR-IS interval defence-gene support	[14]	Host; FPR	Confirmed and new QTL intervals	Defence-related genes occur within QTL intervals	Linkage QTL + interval-level expression support	Expression support exists at interval/category level	No targeted perturbation of a nominated causal gene	Same-pedigree confirmation for selected loci only	E2a positional / E1 for specific gene identity	Interval confirmation does not establish which individual gene is causal.	10.1007/s11295-021-01507-w

Candidate / evidence unit	Citation	Genome / direct pathosystem	Gene ID / locus	Annotation / pathway	Evidence type	Direct molecular support	Functional / perturbational support	Independent replication	FPR evidence level	Principal limitation / interpretive caution	DOI
JA/ET/SA-associated defence programme and hormone crosstalk	[52]	Host; FPR	Pathway-level; individual accessions not harmonised here	Jasmonate–ethylene, salicylate, brassinosteroid/ABA metabolism and stress responses	Direct RNA-Seq + qRT-PCR	Tolerance-dependent host transcriptional response during M. roreri infection	No causal perturbation	Not a locus-replication claim	E3a	Direct molecular support is correlative/pathway-level; individual causal genes remain unvalidated.	10.1111/mpp.12126
Pathogen gene-expression programme associated with infection of tolerant cacao	[53]	Pathogen; FPR	873 differentially expressed M. roreri genes; 43 qRT-PCR validated	Stress responses, HSPs, transporters, transcriptional regulators and metabolism	Direct pathogen-side transcriptomics + qRT-PCR	Direct FPR molecular evidence from infections of susceptible/tolerant clones	No targeted perturbation of individual genes	Not a locus-replication claim	E3a	Correlative transcriptomic evidence; individual pathogen genes have not been functionally tested.	10.1111/mpp.12134
Secreted-protein / effector / cell-wall-degrading-enzyme repertoire	[30]	Pathogen; FPR	Genome/secretome categories	Secretome, candidate effectors and cell-wall-degrading enzyme families	Genome/secretome + phase-associated expression	Direct FPR pathogen molecular evidence at category/repertoire level	No individual causal effector perturbation	Not a locus-replication claim	E3a at category level	Supports functional classes rather than a causally validated individual virulence gene.	10.1186/1471-2164-15-164
ML-prioritised fungal regulatory hubs (including methyltransferase and Hsp20)	[57]	Pathogen; FPR	Candidate regulatory hubs	Regulatory-network / stress-response candidates	Network + machine-learning analysis of infection transcriptomes	Underlying transcripts were measured directly during cacao–M. roreri infection	No perturbational validation	None	E3a, hypothesis-generating	Candidate prioritisation is computational and remains unvalidated experimentally.	10.1007/s10658-025-03154-6
Candidate effector set identified by Nascimento et al.	[18]	Pathogen; FPR	Three functionally tested candidate effectors prioritised from M. roreri infection-stage transcriptional profiling	Secreted candidate effectors / virulence-associated molecular products	Exogenous recombinant-protein functional assay	Candidate genes were identified in direct M. roreri developmental/infection-related transcriptional profiling	Purified recombinant candidate effectors applied exogenously to tomato (Micro-Tom) seedlings produced morphological alterations, wilting and/or necrosis.	No stable pathogen knockout/complementation evidence	E3b-i — exogenous recombinant-protein activity (heterologous plant context)	Biological activity is demonstrated, but no stable M. roreri knockout/complementation has shown that the corresponding genes are required for virulence during a cacao infection cycle.	10.1016/j.ygeno.2025.111083
MrB1/BASIDIN1	[19]	Pathogen; FPR	MrB1 (BASIDIN1 homologue)	Secreted effector-like protein; cellular homeostasis / necrosis-associated activity	Exogenous recombinant-protein functional assay	Direct molecular characterisation in the M. roreri–cacao pathosystem	Recombinant MrB1 applied exogenously to Nicotiana benthamiana cells and cacao leaves/fruits caused cellular disruption, oxidative responses and genotype-dependent/localised necrosis.	No stable knockout/complementation evidence	E3b-i — exogenous recombinant-protein activity (heterologous and cacao tissue contexts)	No stable M. roreri knockout/complementation or host genetic manipulation has demonstrated that MrB1 causally alters FPR during the cognate infection cycle.	10.1111/mpp.70316
MLO-like protein	[16]	Host; WBD directly; FPR only by extrapolation	MLO-like cacao candidate	Susceptibility-gene analogy from powdery mildew; cacao association is WBD-specific	Cross-pathosystem extrapolation	No direct FPR association or expression evidence	No FPR functional test	None for FPR	E0 for FPR	Biological plausibility cannot substitute for direct M. roreri testing.	10.1534/g3.120.401153
TcNPR3	[67,23]	Host; Phytophthora spp. directly; FPR only by extrapolation	TcNPR3	Salicylic-acid defence-regulatory node / susceptibility-gene concept	Transient perturbation + stable CRISPR evidence in another cacao pathosystem	No direct FPR molecular challenge reported	Strong perturbational/stable evidence for Phytophthora spp.; no M. roreri challenge	Not applicable to FPR without direct challenge	E0 for FPR	Functionally strong in Phytophthora pathosystem, but should not be labelled an FPR resistance gene.	10.3389/fpls.2018.00268; 10.1111/pbi.70365

Interpretation. Positional candidates, machine-learning feature importance, direct molecular evidence and perturbational/causal evidence are retained as distinct statistical and biological objects. Pathogen-side FPR evidence reaches E3b-i for exogenous recombinant-effector activity, whereas direct host molecular evidence reaches E3a. No FPR-specific host or pathogen determinant identified in the retrieved and verified literature has reached E4 through stable genetic manipulation producing a reproducible change in a defined FPR component during a biologically appropriate cacao pod–*M. roreri* challenge.

Supplementary Table S4. Candidate leads identified during due diligence but not incorporated as settled evidence.

Candidate lead	DOI	Potential relevance	Why not incorporated as settled evidence at the stated verification date	Future verification action
Ramos-Lizardo GN, Alexiou KG, Mucherino-Muñoz JJ, Aranzana MJ, Corrêa RX. SNP-based genomic variation in candidate disease resistance genes of Theobroma cacao	10.21203/rs.3.rs-9441324/v1	Reports genome-wide/candidate-resistance-gene variation and overlap with disease-resistance QTL intervals.	Research Square preprint; not peer-reviewed at the review cut-off and therefore not incorporated as settled primary evidence.	Re-check publication status and, if peer reviewed, verify the FPR-specific phenotype/locus claims before incorporation.
Barreto MA et al. QTL mapping and identification of corresponding genomic regions for black pod disease resistance to three Phytophthora species in Theobroma cacao L.	10.1101/366054	Methodological comparator for cacao disease-resistance QTL mapping.	Addresses black pod disease (Phytophthora spp.), not M. roreri/FPR; no peer-reviewed superseding publication was verified in the earlier due-diligence pass.	Use only as an explicitly labelled comparator unless a peer-reviewed version and direct FPR relevance are established.
Puig AS, Cohen SP, Koch Bach RA, Arciniegas Leal A, Gutierrez OA, Bailey BA. Comparative RNA-seq analysis of resistant and susceptible Theobroma cacao fruits in response to infection with frosty pod pathogen (Moniliophthora roreri)	10.1094/PHYTOFR-09-22-0101-R	Directly FPR-relevant host transcriptomics; secondary records indicated DEGs overlapping known FPR QTL intervals.	The publisher page/full statistical detail could not be verified to the review's inclusion standard during the due-diligence pass, so it was not used as settled evidence.	Retrieve and verify the publisher version, experimental design, statistics and QTL-overlap claims before updating the Evidence Atlas.
Vasconcelos AA et al. Adaptive evolution of Moniliophthora PR-1 proteins towards its pathogenic lifestyle	10.1186/s12862-021-01818-5	Pathogen-side evolutionary genomics across Moniliophthora, including M. roreri.	Focuses on PR-1 molecular evolution across the genus rather than a direct FPR host-resistance or virulence-validation claim; relevance to the resistance-focused Atlas was therefore uncertain.	Consider in a future pathogen-mechanism update if a claim can be tied directly to the cacao–M. roreri interaction.

Status note. The exclusion status in Table S4 is procedural rather than a judgement that a record is invalid. Each lead was re-checked as part of the complementary update search completed on 1 October 2026 and should be promoted to the Evidence Atlas only when the primary source can be verified to the evidentiary standard required for the corresponding claim.

Supplementary Table S5. Experimental specifications for the decisive FPR research agenda.

Detailed decision matrix supporting the revised research agenda in Section 11 and main-text Table 9. The experiments address distinct claims and are not a compulsory sequence. Their ordering is author-derived, provisional and programme-dependent; no formal weighting, expert elicitation or consensus process was used. GO/NO-GO language denotes proposed decision rules, not established standards. Sample size and replication should be justified prospectively from the hypothesis, expected effect, variance structure and intended scope of inference.

Critical evidence gap	Current evidence / evidence grade	Falsifiable hypothesis	Biological material	Experimental design	Essential controls	Primary FPR endpoint(s)	GO criterion	NO-GO criterion	Evidence transition if confirmed	Recommended action if refuted	Potential breeding / research application	Key supporting sources
1. Phenotype comparability	Primary FPR genetic studies remain heterogeneous in fruit developmental stage, pathogen material, inoculation conditions, experimental unit, disease-rating scales and separation of external versus internal severity. PRG-FPR is proposed as a reporting and design guideline but has not yet been validated through a coordinated inter-laboratory test.	Applying the proposed PRG-FPR reporting/design framework to common biological material will improve between-site comparability of biologically defined FPR phenotypes.	Shared or genetically traceable cacao reference genotypes; traceable, phenotypically characterised M. roreri material where phytosanitary rules permit; common metadata template.	Inter-laboratory or multi-site PRG-FPR validation trial. Use harmonised definitions of incidence, external severity, internal severity and additional disease components, while documenting site-specific experimental parameters rather than imposing universal numerical minima.	Common host reference material; common disease-component definitions; documented fruit stage; inoculum identity/provenance and performance; comparable environmental metadata; independent execution at each site; pre-specified analysis plan.	Incidence; external severity; internal severity; incubation period and/or sporulation only when relevant to the shared hypothesis; concordance of genotype contrasts/rankings across sites.	Harmonisation preserves reproducible biological contrasts and materially reduces avoidable methodological disagreement across sites.	Persistent discordance remains after harmonisation, or key disease components cannot be reproduced with sufficient precision for the intended genetic inference.	Provides empirical evidence on the reproducibility and utility of PRG-FPR; it does not create or raise an E0–E5 evidence class because the classification is claim-specific and multidimensional.	Identify which host, pathogen, developmental, environmental or scoring components drive discordance; revise decision point 1 and the PRG-FPR implementation before denser genomic analysis.	Comparable phenotypes for QTL/GWAS replication, genomic prediction, causal validation and multi-site breeding trials.	Current manuscript Section 3.4 (PRG-FPR); representative FPR mapping/prediction studies: [13] DOI 10.2135/cropsci2006.11.0753; [14] DOI 10.1007/s11295-021-01507-w; [15] DOI 10.3389/fpls.2017.01905
4. Independent locus replication (E2b)	The strongest locus-level FPR evidence reaches E2a because re-detection occurred within the Pound 7 × UF273 pedigree lineage. Other GWAS/ML candidates remain predominantly E1. No FPR locus identified in the review has reached E2b in genetically independent cacao material.	At least one leading FPR locus detected in prior studies will show a directionally consistent association with the biologically corresponding FPR phenotype in genetically independent cacao material.	Independent cacao population not derived from the original discovery pedigree; germplasm sufficiently characterised for population structure and relatedness; PRG-FPR-aligned phenotypes.	Pre-specified replication of the strongest locus-level claims. Test the original locus/interval or clearly defined proxy before exploratory genome-wide reanalysis. Control population structure/relatedness and justify sample size prospectively by power or precision analysis.	Independent genetic material; phenotype matched biologically to the original claim; pre-specified locus and primary analysis; appropriate population-structure/kinship correction; transparent handling of multiple testing where applicable.	The same FPR disease component used for the original claim, or an explicitly justified biologically equivalent phenotype; effect direction and uncertainty reported.	Association replicates in independent material with the expected direction and pre-specified statistical support.	Association fails to reproduce, reverses direction, or depends on a phenotype/population definition incompatible with the original claim.	E2a → E2b for the specific replicated locus–phenotype claim.	Restrict the claim to E1/E2a; lower priority for MAS and expensive Frontier work; retain only as context-specific evidence unless further independent support emerges.	Evidence-qualified marker prioritisation, improved locus transferability assessment and stronger mechanistic candidate selection.	[13] DOI 10.2135/cropsci2006.11.0753; [14] DOI 10.1007/s11295-021-01507-w; [61] DOI 10.3389/fpls.2018.00343
2. Prediction transferability	Direct FPR genomic prediction is encouraging within characterised populations, but cross-population predictive performance can decline substantially when genetic relatedness between training and target material is reduced.	A genomic prediction model trained on a defined breeding population will retain programme-relevant predictive value in the prospectively specified target population, family set, breeding cycle or environment domain.	Training population with PRG-FPR-aligned phenotypes and genome-wide markers; genetically distinguishable target material whose relevant phenotypes remain hidden during prediction.	Prospectively defined cross-population, cross-family or cross-cycle validation. Characterise relatedness between training and target material; compare internal validation with prediction outside the original training domain; evaluate recalibration separately from true transfer.	Target phenotypes withheld until predictions are locked; predefined target domain; fixed prediction model/feature set before outcome observation; appropriate baseline comparator; transparent treatment of relatedness and population structure.	FPR phenotype(s) used by the breeding programme; predictive ability/accuracy; candidate ranking stability; realised phenotype of selected candidates when available.	Prediction retains sufficient decision value in the intended target domain relative to the programme's realistic alternative.	Predictive performance collapses, rankings are unstable, or the model offers no meaningful decision advantage outside the training domain.	Does not map to a single E-grade automatically; qualifies or broadens decision point 2 use within the validated prediction domain.	Restrict model use to the demonstrated domain; redesign or enlarge the training population; improve genetic connectivity; collect additional phenotypes; recalibrate and re-test.	Earlier or more accurate selection, training-population design, multi-cycle genomic selection and definition of prediction domains.	[15] DOI 10.3389/fpls.2017.01905 ; [61] DOI 10.3389/fpls.2018.00343
6. Stable causal validation (E4)	Direct host molecular evidence reaches mainly E3a, while recent pathogen effector assays reach E3b-i exogenous recombinant-protein activity. No FPR-specific host or pathogen determinant identified in the review has reached	Stable manipulation of a prioritised host or pathogen determinant will reproducibly alter a biologically defined FPR outcome during direct cacao–M. roreri interaction.	Host route: stable cacao alterations with appropriate matched controls and independent transformation events where feasible. Pathogen route: stable M. roreri knockout/knock-in or equivalent manipulation with	Stable genetic causal test using a biologically appropriate cacao pod–M. roreri challenge and disease definitions/reporting aligned with PRG-FPR. Confirm the intended molecular alteration and match disease components to	Matched genetic background; molecular confirmation; appropriate host/pathogen controls; independent events or strains where feasible; complementation or allelic rescue where technically applicable;	Incidence, external severity, internal severity, incubation period, time to sporulation and/or longitudinal progression according to the mechanistic hypothesis; pathogen biomass where relevant.	Stable manipulation produces a reproducible directional change in the predicted FPR phenotype relative to appropriate controls.	The intended manipulation is confirmed but the predicted disease phenotype does not change, fails across independent events, or cannot be separated from background/regeneration effects.	E4 for the specific causal claim within the tested cacao–M. roreri context; whole-plant phenotyping is not intrinsically required for pod-disease causality.	Reject or narrow the causal hypothesis; retain valid lower-level evidence for association, expression or functional activity; redirect Frontier resources to other candidates or mechanisms.	Causal target validation, mechanistic dissection, host-intervention prioritisation, pathogen virulence biology and evidence-based editing decisions.	[52] DOI 10.1111/mpp.12126; [53] DOI 10.1111/mpp.12134; [18] DOI 10.1016/j.ygeno.2025.111083; [19] DOI 10.1111/mpp.70316

Critical evidence gap	Current evidence / evidence grade	Falsifiable hypothesis	Biological material	Experimental design	Essential controls	Primary FPR endpoint(s)	GO criterion	NO-GO criterion	Evidence transition if confirmed	Recommended action if refuted	Potential breeding / research application	Key supporting sources
	stable causal validation (E4).		parental controls and, where applicable, complementation or allelic rescue.	the causal hypothesis; broaden host backgrounds or isolates only when broadening the claim.	standardised or blinded scoring where practical.							
5. Host × pathogen × environment breadth	M. roreri is genetically structured and isolates can differ phenotypically, but molecular lineage does not reliably substitute for aggressiveness. Effects observed in one host × isolate × environment combination cannot establish broad-spectrum or durable performance.	A selected resistant genotype or E4-supported host intervention will retain a favourable FPR effect and acceptable agronomic performance across the host, pathogen and environmental domain for which deployment is proposed.	Genetically contrasting cacao backgrounds; more than one phenotypically characterised M. roreri isolate or geographically informative inoculum source where regulations permit; representative target environments.	Connected multi-host × multi-isolate × multi-environment validation. Use factorial or otherwise connected designs sufficient to estimate host, pathogen, environment and interaction effects. Maintain common PRG-FPR disease definitions and include productivity/quality when deployment relevance is claimed.	Traceable host genotypes; phenotypically characterised pathogen material; shared reference controls where possible; common disease definitions; environmental metadata; randomisation/blocking appropriate to site; quarantine and phytosanitary compliance.	Defined FPR disease components plus productivity, adaptation and quality for deployment-oriented claims; interaction terms and stability of the favourable response.	Direction and agronomic acceptability of the favourable FPR effect remain sufficiently stable across the intended deployment domain.	Strong dependence on one host background, isolate or environment; inconsistent effect direction; unacceptable productivity or quality trade-offs.	Broadens the scope of E4/decision point 4 or deployment claims; does not create E5 without prospective breeding utility.	Narrow the claim or deployment domain; diversify the deployment portfolio; introduce alternative backgrounds/resistance sources; redesign the intervention rather than declaring the mechanism universally invalid.	Durability assessment, deployment zoning, resistance portfolio design, pathogen-aware breeding and post-release monitoring.	[32] DOI 10.1111/j.1365-3059.2005.01210.x; [28] DOI 10.3389/fmicb.2015.00850; [29] DOI 10.1094/PDIS-12-15-1498-RE
3. Prospective breeding utility (E5)	No FPR marker, genomic-prediction model or molecular intervention identified in the review has demonstrated E5. Retrospective association, internal predictive accuracy, E2b replication or E4 causality do not by themselves establish breeding utility.	Prospective use of an evidence-informed marker, genomic model, multi-trait index or validated host intervention will improve a predefined breeding outcome relative to the programme's realistic current alternative.	Real breeding candidates: parents, families, clones or alleles eligible for advancement; the comparator strategy used routinely or realistically by the breeding programme.	Prospective selection experiment. Lock the candidate set, decision rule, comparator and primary breeding outcome before realised validation phenotypes are known. Use the new information to determine advancement, then evaluate selected material under relevant conditions.	Predefined comparator; outcomes specified before observation; candidate phenotypes withheld until selection decisions are fixed; transparent costs/cycle-time accounting when claimed as benefits; independent follow-up phenotyping.	Realised FPR performance plus the programme's multi-trait objective (productivity, adaptation, quality); cycle time, selection accuracy, realised response or genetic gain per unit time when relevant.	Use of the information produces a predefined, documented improvement in realised breeding outcome within the stated programme/context.	No meaningful prospective advantage over the comparator, expected gains are not realised, transferability is too narrow, or added cost/complexity is not justified by the breeding benefit.	E5 — demonstrated breeding utility for the specific programme, population and decision context.	Withhold operational adoption; recalibrate, restrict or abandon the strategy while retaining the underlying E1–E4 or predictive evidence at its valid level.	Operational MAS/GS, prospective multi-trait selection, evidence-based deployment and realised genetic gain.	[15] DOI 10.3389/fpls.2017.01905; [61] DOI 10.3389/fpls.2018.00343; current manuscript Sections 9.2 and 9.7

Evidence-classification data dictionary

Level	Operational interpretation
E0	Directness qualifier: the FPR claim is supported only by evidence from another disease, pathosystem or species; the underlying evidence retains its own class in its original system.
E1	Genetic axis: statistical/positional association or discovery-stage evidence in one population or dataset, without independent replication.
E2a	Genetic axis: re-detection, refinement or fine-mapping in genetically dependent material sharing individuals, parents or a breeding lineage with the discovery population.
E2b	Genetic axis: replication of the same locus/interval and comparable phenotype in genetically independent material, with relatedness documented.
E3a	Mechanistic axis: direct molecular/omics support measured in the cacao–M. roreri interaction that remains correlative.
E3b-i	Mechanistic axis: exogenous application of a purified/recombinant candidate product demonstrates biochemical, cellular or tissue activity; context must be stated.
E3b-ii	Mechanistic axis: transient or heterologous in planta expression/perturbation modulates host molecular, cellular or tissue responses but does not establish contribution to disease.
E3b-iii	Mechanistic axis: non-stable perturbation of an endogenous host or pathogen determinant during cacao-pod infection changes a defined FPR component.
E4	Mechanistic axis: stable, heritable host or pathogen manipulation produces a reproducible directional change in a defined FPR component during a biologically appropriate cacao pod–M. roreri challenge, supported by independent transformation events or pathogen strains and, where applicable, genetic complementation. Whole-plant phenotyping is not required for pod-disease causality.
Validation descriptor	Predictive validation scope is recorded rather than graded: within-population random CV; family/cycle/environment-structured validation; external population validation with relatedness stated; or prospective prediction.
E5	Translational axis: prospective use of a marker, model, index or intervention in an operational breeding/deployment decision produces documented improvement against a pre-specified comparator and outcome.
